# XGBoost (eXtreme Gradient Boosting) Can Predict Organisms Growing in Urine Culture from the Emergency Department

**DOI:** 10.5811/westjem.48715

**Published:** 2026-04-08

**Authors:** Johnathan M. Sheele, Ronna L. Campbell, Derick D. Jones

**Affiliations:** * Mayo Clinic, Department of Emergency Medicine, Jacksonville, Florida; † Mayo Clinic, Department of Emergency Medicine, Rochester, Minnesota

## Abstract

**Introduction:**

Urinary tract infections are common in the emergency department (ED) but are frequently misdiagnosed and mismanaged. We sought to determine whether eXtreme Gradient Boosting (XGBoost), an open-source machine-learning library, could predict the organisms growing in urine cultures ordered from the ED.

**Methods:**

We developed XGBoost algorithms to retrospectively examine 62,963 Mayo Clinic ED encounters between January 1, 2017–December 31, 2021, during which a urinalysis and urine culture were performed. The model used 1,303 patient variables. All patient ages were included. Data were from the electronic health record and available to the clinician during the patient encounter.

**Results:**

For the most common bacteria growing in urine culture, XGBoost was able to predict the presence of a member of the *Enterobacteriaceae* family with an area under the receiver operating curve (AUC) of 0.90 and an accuracy of 0.79. The model predicted the presence of 10 different bacterial genera with an AUC of 0.70–0.88 and an accuracy of 0.87–0.99. Furthermore, XGBoost was able to predict whether the urine culture would report Gram-positive or Gram-negative bacteria with an AUC of 0.81 and 0.90, respectively, and an accuracy of 0.85 and 0.86, respectively. The model predicted whether yeast would be reported with an AUC of 0.84 and an accuracy of 1.00.

**Discussion:**

XGBoost can predict the bacterial genus and Gram-staining results of the bacteria growing in urine cultures.

## INTRODUCTION

Hospital admission costs for diagnosing and treating urinary tract infection (UTI) in the U.S. exceed $2 billion annually and increased 52% between 2008–2011.^1^ Urinary tract infections are among the most common reasons for administering antibiotics in the emergency department (ED), yet urine cultures of patients diagnosed with UTIs in the ED may be negative.^2^–^6^ Having a false-positive UTI diagnosis can lead to unnecessary antibiotic adverse effects and drug-resistant bacterial infections. If an emergency clinician could have knowledge about a uropathogen growing in urine culture during the clinical encounter, it would improve the number of patients correctly diagnosed with a UTI and facilitate the use of appropriate antibiotics, which have been shown to reduce patient morbidity, mortality, and overall healthcare costs.^7^

More than 600 bacterial species have been cultured from urine.^8^ An analysis of 16S ribosomal RNA found a median of 41 bacterial genera per urine sample.^9^ *Escherichia coli* causes most uncomplicated, community-based UTIs. Several other organisms are also known to cause UTIs, including *Pseudomonas aeruginosa*, *Proteus* species, *Klebsiella* species, *Acinetobacter* species, *Enterobacter* species, *Citrobacter* species, *Staphylococcus saprophyticus*, coagulase-negative *Staphylococcus*, and *Enterococcus* species.^10^ A patient’s age, comorbid conditions, existing genitourinary pathology, indwelling urethral catheters, and immunodeficiencies could all influence whether a particular uropathogen is growing in the urine.^11^

Traditional urine cultures do not report all bacterial species growing in the urine; instead, they are designed to report the most prevalent uropathogens.^9^ Not all bacteriuria indicates a UTI; some bacteriuria may be asymptomatic or represent contamination during urine sample collection.^12^ Molecular diagnostic tests identify more bacteria than traditional culture techniques; however, it is unclear whether this additional information improves clinical care.^13^ Neither is it clear whether patients who have UTI symptoms but a negative urine culture and negative workup for other genitourinary pathology may benefit from antibiotics.

While machine learning has been used previously to predict bacterial antibiotic resistance in patients with UTIs,^14^–^18^ we found no reports of it being used to identify the infecting organism reported on the urine culture. In this study we aimed to determine whether eXtreme Gradient Boosting (XGBoost) could predict the bacterial organism from urine cultures obtained in the ED. XGBoost is an open-source, machine-learning library that incorporates gradient-boosted decision trees. Secondly, we examined whether XGBoost could predict the Gram stain result of the bacteriuria and the presence of yeast. If accurate models can be developed they could theoretically be incorporated into the electronic health record (EHR) to aid in real-time clinical decision-making.

## METHODS

### Study Design and Setting

Mayo Clinic’s data retrieval team created a dataset from the EHRs of all Mayo Clinic EDs (Florida, Minnesota, Arizona, Iowa, and Wisconsin) between January 1, 2017–December 31, 2021, with no age restrictions. The research was conducted in accordance with TRIPOD+AI (Transparent Reporting of a Multivariable Prediction Model for Individual Prognosis or Diagnosis + Artificial Intelligence) and followed guidelines on retrospective research (with the exception that no data abstractors were used).^19^,^20^ Included patients provided research authorization and had a urinalysis or urine culture. For our analysis, data had to meet the following additional criteria: complete urinalysis values (ie, no missing urinalysis values); both a urinalysis and urine culture ordered from 4 hours before to 8 hours after the ED arrival date and time; urine culture results obtained < 10 days after the culture was collected; diagnosis of UTI during the ED encounter; and documented encounter admission and discharge dates and times, event start and end dates and times; and diagnosis dates. The Mayo Clinic Institutional Review Board approved the study.

### Variables and Outcomes

Data in our analysis included the following: patient demographics and social history; diagnoses made before urine culture results; medications; past medical histories; temperature; radiology studies performed and their results; admission and disposition information; medications ordered before and during the ED encounter; current and prior laboratory data; reproductive health information; genitourinary nursing procedures and flowsheets; fall-risk assessments done by nurses; allergies; and prior microbiology results. Each patient encounter was tracked with unique identifiers. We included formulas of inflammation (eg, neutrophil-lymphocyte ratio, urinary inflammatory index, systemic immune-inflammation index, and platelet-lymphocyte ratio), ratios, and mathematical formulas we invented using laboratory values [available upon request].

**Population Health Research Capsule**

What do we already know about this issue?

*The bacterial genera or species causing a urinary tract infections (UTI) are most commonly unknown during the clinical encounter*.

What was the research question?

*To determine if a machine learning algorithm could predict the bacterial genera or species growing in urine culture*.

What was the major finding of the study?

*Our XGBoost model predicted the presence of 10 different bacterial genera growing in urine culture with an AUC of 0.70–0.88 and an accuracy of 0.87–0.99*.

How does this improve population health?

*Predicting the bacteria growing in urine culture could potentially assist with antimicrobial stewardship and improve the ability to accurately diagnose UTI*.

We included encounter diagnoses in our model if the ED discharge departure, event encounter, and diagnosis occurred before the urine culture result was available; otherwise, it was coded as “no.” Encounter diagnoses were identified using text searches and *International Classification of Diseases, 10^th^Ed*,, codes (Supplement 1). The presence of yeast, the bacterial family, genus, or species, and whether the bacteria were Gram-positive or Gram-negative was determined using text searches in Excel (Microsoft Corporation, Redmond, WA) (available upon request). We included in our analysis only those bacterial genera present in more than 500 cultures and > 0.5% frequency.

### Statistical Analysis

Variables were modeled as continuous (numerical) or categories, or sometimes both. If information was missing we marked it as “not available” for categories, and for numbers we filled in the missing spots with the group’s median value. All outcomes had binary outcomes (ie, positive or negative for the outcome). The data were then split randomly: 80% for training the model; and 20% for testing it. For categories, we did one-hot encoding to convert them into a format the computer could read. Because some outcomes were much less common than others, we used a method called Synthetic Minority Over-sampling Technique (SMOTE) to create extra examples so that the model could learn more evenly. We also adjusted the scale of the numbers so that everything was on the same level. The XGBoost model was run with its default settings, using an early stopping rule and a “log loss” measure to track accuracy. XGBoost works by building a series of decision trees, each one improving on the mistakes of the last, while adding checks to avoid overfitting and to keep predictions accurate. We applied default hyperparameters to the XGBoost model, which was configured with default settings and early stopping rounds with a log-loss evaluation metric.

We report the F1, precision, recall, score, and accuracy of our XGBoost models. The F1 score is the mean of precision and recall. Precision is also known as the positive predictive value (ie, the number of true positives divided by the number of true positives plus false positives). Recall is also known as sensitivity (ie, the number of true positives divided by the number of true positives plus false negatives). Score is a performance metric for the model. Accuracy is the percentage of predictions that the model correctly predicted.

## RESULTS

We included 62,963 encounters in our analysis, of which 48,069 (76.3%) were from unique patients. The median (IQR) age was 64 (39). There were 3,942 (6.3%) encounters in patients < 18 years of age, and 40,582 patients (64.5%) were female. A total of 56,286 (89.4%) encounters were with White patients; 2,919 (4.6%), Black; 1,241 (2.0%) Asian; and 2,517 (4.0%) encounters were with patients of unknown or “other” race. Testing for gonorrhea, chlamydia, or trichomonas occurred in 1,926 encounters. A summary of patient demographics is provided in Supplement 2.

Of 62,963. urine cultures tested, 18,128 (28.8%) had no microbial growth, 26,999 (42.9%) had ≥ 10,000 colony-forming units per milliliter (CFU/mL) bacterial growth of ≥ 1 organisms, and 16,703 (26.5%) had ≥ 100,000 CFU/mL bacterial growth of ≥ 1 organisms. XGBoost predicted the presence of a member of the E*nterobacteriaceae* family in the urine culture with an area under the receiver operating curve (AUC) of 0.90 (Table 1) (Supplement 3). The model predicted the bacterial genus growing in urine culture with an AUC of 0.70 for *Streptococcus* species and up to 0.88 for *Escherichia* species (Table 1) (Supplement 3). It was further able to predict whether bacteria would be Gram-positive or Gram-negative with AUCs of 0.81 and 0.90, respectively (Table 1) (Supplement 3). XGBoost also predicted whether yeast would be reported (AUC, 0.84) and whether 1 or 2 vs > 2 bacterial genera would be reported (AUC, 0.89) (Table 1) (Supplement 3).

We also separately analyzed encounters from patients who likely had a true UTI (≥ 10,000 CFU/mL of ≤ 2 bacterial genera or species on urine culture) and received a diagnosed of a UTI in the ED.^21^–^28^ Our XGBoost model was able to predict the following bacteria (AUC): *Escherichia* species (0.72); *Klebsiella* species (0.68); *Enterococcus* species (0.74) *Proteus* species (0.75); *Pseudomonas* species (0.73); Gram-positive bacteria (0.74), and Gram-negative bacteria (0.76) (Table 2).

## DISCUSSION

Our results show that XGBoost can predict both the organism growing in culture and the Gram stain results for bacteriuria. Our model could predict whether a member of the *Enterobacteriaceae* family, which represents the majority of uropathogens causing UTI (eg, *Klebsiella*, *Enterobacter*, *Citrobacter*, *Escherichia coli*, *Proteus*, and *Serratia*), is growing in culture with excellent accuracy (AUC, 0.90). The model was also able to predict whether yeast would be reported on urine culture and whether 1 or 2 bacterial genera vs > 2 bacterial genera would be reported. Notably, our model performed well in the subset of patients most likely to have a UTI.

Our work builds on that of others who have shown that machine learning can aid clinicians in diagnosing UTIs. We found no studies using XGBoost to predict the organisms growing in the urine culture, but others have used machine learning to predict antibiotic resistance.^14^–^18^ Factors that predict antibiotic sensitivity include past antibiotic use, prior urine culture results, age, ethnicity, and comorbid conditions such as diabetes, genitourinary tract pathology, kidney stones, travel history, and place of residency; these are also likely to be important in predicting the specific bacteria growing in the culture.^7^,^29^–^31^ For instance, children with spina bifida were more likely to have non-*E coli* UTIs.^32^ Additionally, the following were associated with Gram-positive UTIs in children: age; serum white blood cells; no prior antibiotic use; C-reactive protein; hemoglobin; negative urine leukocyte esterase; negative urine nitrite; and white blood cells in urine.^33^ Urinalysis-specific findings may help predict bacteriuria. For instance, *Proteus mirabilis* UTIs are associated with more alkaline urine.^34^

A previous study reported that UTIs are frequently misdiagnosed and mismanaged in the ED.^5^ Machine-learning prediction models may be a strategy for improving UTI management by correctly predicting which organisms will eventually grow in the urine culture and providing this information to the clinician during the clinical encounter. This knowledge could help clinicians make more informed decisions about whether to initiate antibiotics as well as facilitate use of the most appropriate antibiotic. We envision that our XGBoost algorithms would continuously run in the background of the health system’s EHR on all patients who receive a urinalysis. The algorithm continuously updates for each patient as new data is generated during their clinical encounter (eg, new lab results). The emergency clinician could access the algorithm results at any point during the patient’s clinical encounter, but the most accurate predictions would occur at the end of the ED encounter when most of the variable data has been incorporated into the models. The model is not yet ready for clinical practice and requires additional validation.

## LIMITATIONS

Patients in our dataset were likely to be more chronically ill, elderly, immunocompromised, and have existing genitourinary pathology than a typical community ED. Our data were also somewhat limited by geographic location, as was it limited by insufficient racial and ethnic diversity. Our models are only applicable for patients who get both a urinalysis and a urine culture. Not all patients diagnosed with a UTI get a urine culture; thus, our models may not be applicable to all patients with a UTI. However, studies have shown that ED patients meeting a clinical diagnosis of UTI have high rates of negative urine cultures suggesting they likely do not have a bacterial UTI.^2^,^35^,^36^ Conversely, not all patients with a positive urine culture have a UTI (eg, asymptomatic bacteriuria), and our models do not predict UTI. It is unclear whether the rates of our urine culture being positive would affect the model’s performances if the study were replicated elsewhere.

Our analysis only included patients presenting to the ED; therefore, our results may only be generalizable to some patient populations. Our dataset did not include patient history or physical exam findings taken in the ED, and it is unclear whether adding this information would improve our model. Gram staining was not performed on our urine culture results; instead we used known Gram-staining characteristics for the bacteria reported in the culture. Our XGBoost model included more than 1,300 variables; future research will be essential to see how the model performs with fewer variables. Lastly, it was not practical to report out all our XGBoost algorithms decision trees because of the thousands of branches for each model.

## CONCLUSION

XGBoost can predict the organisms growing in ED urine cultures using data available to the emergency clinician during the clinical encounter. Correctly predicting uropathogens growing in urine cultures during the clinical examination may improve accuracy in diagnosing and treating UTIs. Our XGboost models could potentially be designed to supplement clinical judgment for diagnosing UTIs during the ED visit but will not advise the clinician whether the patient should be treated with antibiotics.

## Supplementary Information







## Figures and Tables

**Table 1:** Performance characteristics of XGBoost models used to predict the organism reported in a urine culture.

Outcome	Urine cultures % (N = 62,963)*	Precision (PPV)	Recall (Sensitivity)	F1 Score (mean of PPV and Sensitivity)	Accuracy	AUC
Gram-positive bacteria (n = 10,601)	16.8	0.74	0.67	0.69	0.85	0.81
Gram-negative bacteria (n = 17,888)	28.4	0.83	0.81	0.82	0.86	0.90
*Aerococcus* sp (n =. 534)	0.8	0.66	0.51	0.51	0.99	0.87
*Citrobacter* sp (n = 521)	0.8	0.50	0.50	0.50	0.99	0.78
*Enterobacteriaceae* family (n = 15,171)	24.1	0.81	0.80	0.81	0.86	0.90
*Enterobacter* sp (n = 860)	1.4	0.58	0.50	0.50	0.99	0.81
*Enterobacter cloacae* (n = 545)	0.9	0.50	0.50	**0.50**	**0.99**	**0.76**
*Enterococcus* sp (n = 2,060)	**3.3**	**0.59**	**0.51**	**0.51**	**0.97**	**0.75**
*Enterococcus faecalis* (n = 1,826)	2.9	0.58	0.51	0.51	0.97	0.74
*Escherichia* sp (n = 10,456)	16.6	0.77	0.73	0.74	0.87	0.88
*Klebsiella* sp (n = 3,034)	4.8	0.71	0.53	0.55	0.95	0.84
*Proteeae* group (n = 1,207)	1.9	0.71	0.55	0.57	0.98	0.82
*Proteus* sp (n = 971)	1.5	0.71	0.54	0.56	0.98	0.82
*Pseudomonas* sp (n = 1,214)	1.9	0.63	0.53	0.55	0.98	0.84
*Staphylococcus* sp (n = 1,427)	2.3	0.77	0.51	0.51	0.98	0.72
*Staphylococcus aureus* (n=455)	0.7	0.50	0.50	0.50	0.99	0.74
Coagulase-negative *Staphylococcus* (n = 979)	1.6	0.74	0.51	0.52	0.98	0.74
*Streptococcus* sp (n = 1,209)	1.9	0.49	0.50	0.50	0.98	0.70
*Streptococcus agalactiae* and *Streptococcu*s group B (n = 791)	1.3	0.49	0.50	0.50	0.99	0.72
Yeast (n = 204)	0.3	0.50	0.50	0.50	1.00	0.84
1–2 (n = 19,345) vs > 2 (n = 7,654) bacterial genera or species in culture^b^	NA	0.82	0.81	0.81	0.84	0.89

* Cultures can report ≥ 1 organisms. Note: Urine cultures with no microbial growth were ignored in the model.

*AUC*, area under the receiver operating curve; *NA*, not applicable; *PPV*, positive predictive value; *sp*, species; *XGBoost*, eXtreme Gradient Boosting.

**Table 2:** Performance characteristics of XGBoost models used to predict the organism reported in urine cultures with ≥ 10,000 CFU/mL of ≤ 2 bacterial genera/species from patients diagnosed with urinary tract infection before culture results.

Outcome	Urine cultures % (N = 9,241)*	Precision	Recall	F1 Score	Accuracy	AUC
Gram-positive bacteria (n = 2,126)	23.0	0.68	0.62	0.64	0.78	0.74
Gram-negative bacteria (n = 7,886)	85.3	0.64	0.58	0.59	0.84	0.76
*Aerococcus* sp (n = 216)	2.3	0.85	0.57	0.62	0.98	0.81
*Citrobacter* sp (n = 205)	2.2	0.61	0.51	0.51	0.98	0.64
*Enterobacteriaceae* family (n = 7,484)	81.0	0.62	0.58	0.59	0.79	0.74
*Enterobacter* sp (n = 336)	3.6	0.48	0.50	0.49	0.96	0.64
*Enterobacter cloacae* (n = 221)	2.4	0.49	0.50	0.49	0.97	0.63
*Enterococcus* sp (n = 643)	7.0	0.62	0.54	0.55	0.92	0.74
*Enterococcus faecalis* (n = 582)	6.3	0.59	0.52	0.53	0.93	0.75
*Escherichia* sp (n = 5,531)	59.9	0.65	0.65	0.65	0.67	0.72
*Klebsiella* sp (n = 1,211)	13.1	0.64	0.54	0.55	0.87	0.68
*Proteeae* group (n = 479)	5.2	0.64	0.56	0.58	0.95	0.75
*Proteus* sp (n = 387)	4.2	0.71	0.53	0.55	0.96	0.75
*Pseudomonas* sp (n = 413)	4.5	0.62	0.55	0.56	0.95	0.73
*Staphylococcus* sp (n = 605)	6.5	0.61	0.52	0.51	0.93	0.73
*Staphylococcus aureus* (n = 160)	1.7	0.49	0.50	0.49	0.98	0.64
Coagulase-negative *Staphylococcus* (n = 446)	4.8	0.65	0.53	0.54	0.95	0.79
*Streptococcus* sp (n = 295)	3.2	0.48	0.50	0.49	0.96	0.68
*Streptococcus agalactiae* and *Streptococcus* group B (n = 168)	1.8	0.74	0.54	0.57	0.98	0.82

* Cultures can report ≥ 1 organism. Note: The model ignored urine cultures with no microbial growth .

*AUC*, area under the receiver operating curve; CFU, colony forming units; *NA*, not applicable; *sp*, species; *XGBoost*, eXtreme Gradient Boosting.
