# Supplementary material for: XGBoost (eXtreme Gradient Boosting) Can Predict Organisms Growing in Urine Culture from the Emergency Department
Source: West J Emerg Med. 2026 Apr 8;27(3):759–65. doi: 10.5811/westjem.48715 (PMC13246185; doi:10.5811/westjem.48715)
Supplement: Supplementary file 1 [file wjem-27-759-s001.docx]

Supplement 5.

ICD-10 codes used to define a UTI:

N39.0, O08.83, O04.88, O86.20, O23.40, P39.3, N30.90, N30.00, N30.80, N30.91, N30.01, N30.81, O86.22, O23.11, O23.12, O23.13, O23.10, O03.88, O07.38, O03.38, O04.88, O23.41, O23.42, O23.43, N10, N13.6, O86.21, O23.0, N15.1, N28.86
