# Supplementary material for: XGBoost (eXtreme Gradient Boosting) Can Predict Organisms Growing in Urine Culture from the Emergency Department
Source: West J Emerg Med. 2026 Apr 8;27(3):759–65. doi: 10.5811/westjem.48715 (PMC13246185; doi:10.5811/westjem.48715)
Supplement: Supplementary file 2 [file wjem-27-759-s002.docx]

Supplement 2. Demographics of our cohort (previously published)

| Variable | Whole Cohort |
| --- | --- |
| Age in years | 60.1-80.0: 21194 (33.66%), >=80.1: 13908 (22.09%), 40.1-60.0: 11792 (18.73%), 14.1-30.0: 7651 (12.15%), 30.1-40.0: 5202 (8.26%), Other: 3216 (5.11%) |
| Female gender | F: 40582 (64.45%), not female: 22381 (35.55%) |
| Marital status | Married: 29146 (46.29%), Single: 17304 (27.48%), Widowed: 9726 (15.45%), Divorced: 5519 (8.77%), Separated: 578 (0.92%), Other: 690 (1.10%) |
| Race | White: 56286 (89.40%), Black_African_African_American: 2919 (4.64%), Unknown, other, not native american, pacific islander: 2517 (4.00%), Asian: 1241 (1.97%) |
| Ethnicity | Not Hispanic or Latino: 58107 (92.29%), Hispanic or Latino_spanish_speaking: 3887 (6.17%), unknown: 969 (1.54%) |
| OB/Gyn status | postmenopause/no uterus/amenorrhea/hysterectomy: 24913 (39.57%), male: 22386 (35.55%), having periods/irregular periods/premenarcheal: 6324 (10.04%), birth control present: 4081 (6.48%), unknown: 3857 (6.13%), Other: 1402 (2.23%) |
