# Supplementary material for: XGBoost (eXtreme Gradient Boosting) Can Predict Organisms Growing in Urine Culture from the Emergency Department
Source: West J Emerg Med. 2026 Apr 8;27(3):759–65. doi: 10.5811/westjem.48715 (PMC13246185; doi:10.5811/westjem.48715)
Supplement: Supplementary file 3 [file wjem-27-759-s003.pdf]

ROC Curves by Endpoint

Generated from XGBoost models with scaling and SMOTE (when feasible).

- Binary endpoints: standard ROC/AUC
- Multiclass endpoints: macro-average ROC/AUC (OvR)

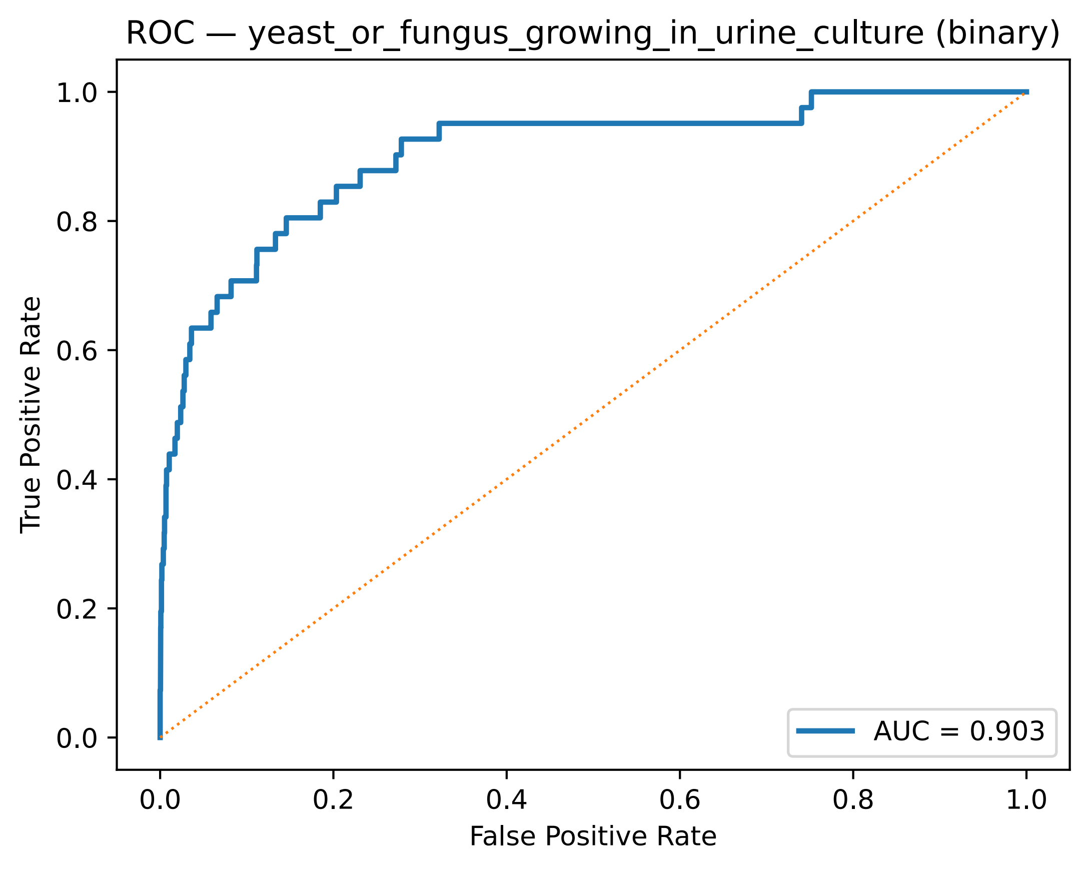
ROC — yeast_or_fungus_growing_in_urine_culture (binary)
True Positive Rate
False Positive Rate
0.0
0.2
0.4
0.6
0.8
1.0
AUC = 0.903

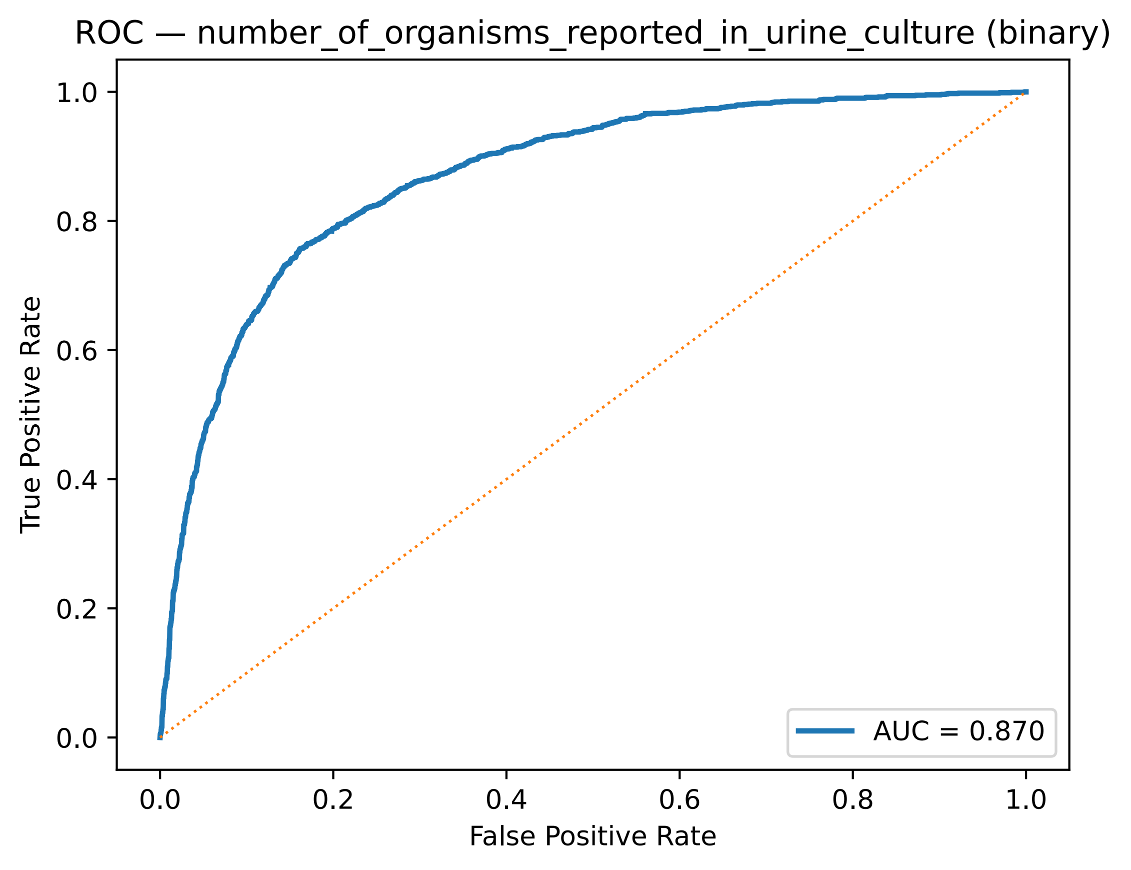
ROC — number_of_organisms_reported_in_urine_culture (binary)
1.0
0.8
0.6
0.4
0.2
0.0
True Positive Rate
0.0
0.2
0.4
0.6
0.8
1.0
False Positive Rate
AUC = 0.870

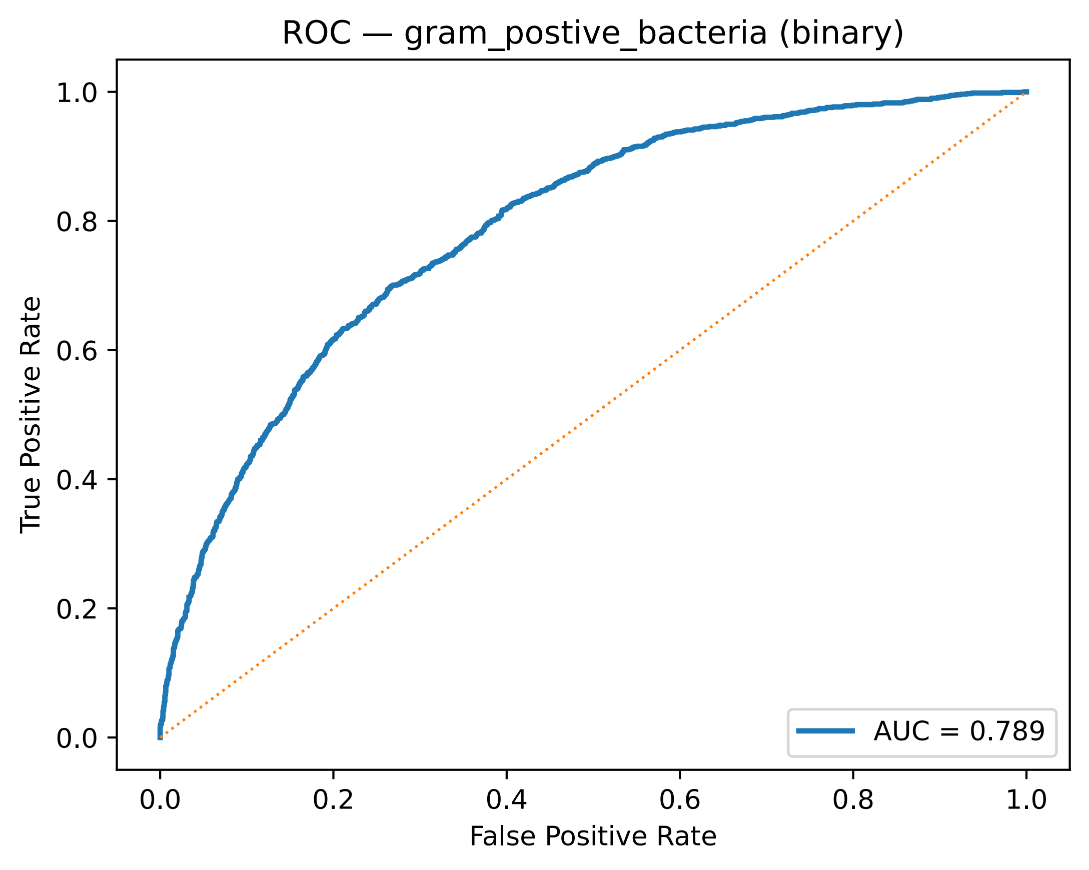
ROC — gram_postive_bacteria (binary)
True Positive Rate
False Positive Rate
0.0
0.2
0.4
0.6
0.8
1.0
AUC = 0.789

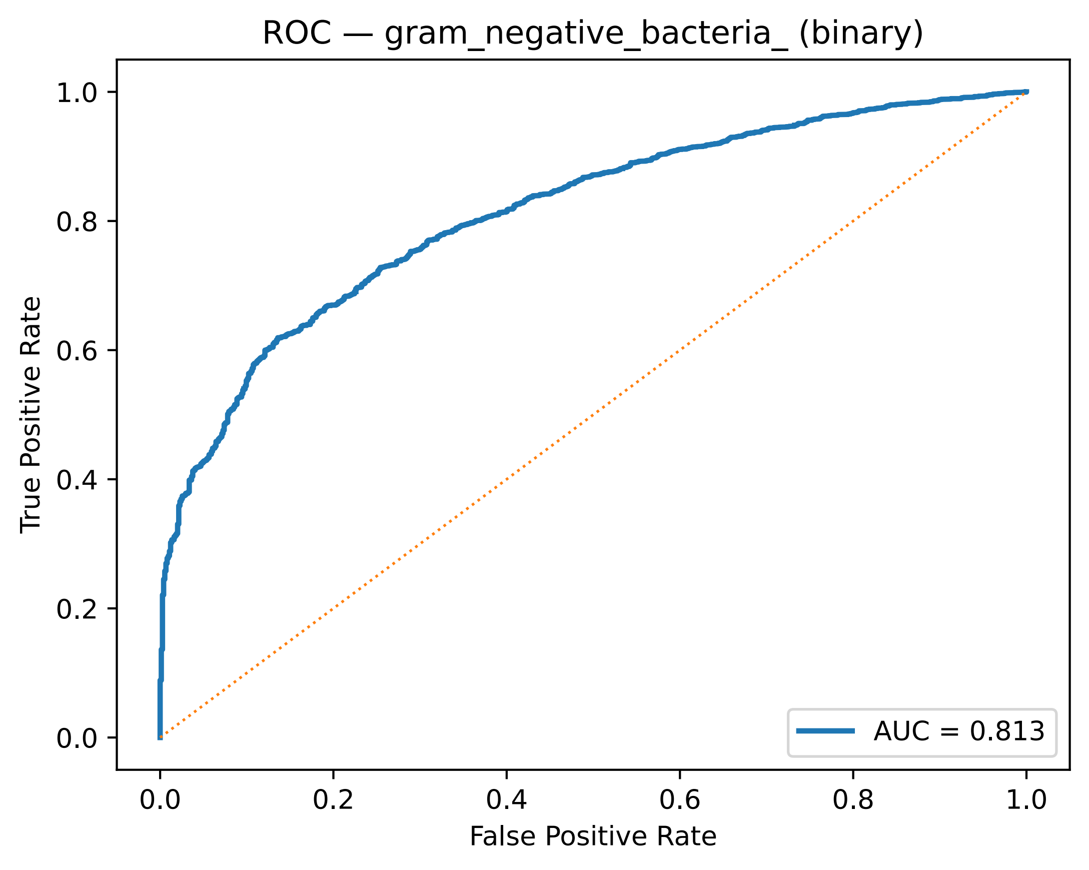

ROC — gram_negative_bacteria_ (binary)
True Positive Rate
False Positive Rate
0.0
0.2
0.4
0.6
0.8
1.0
AUC = 0.813

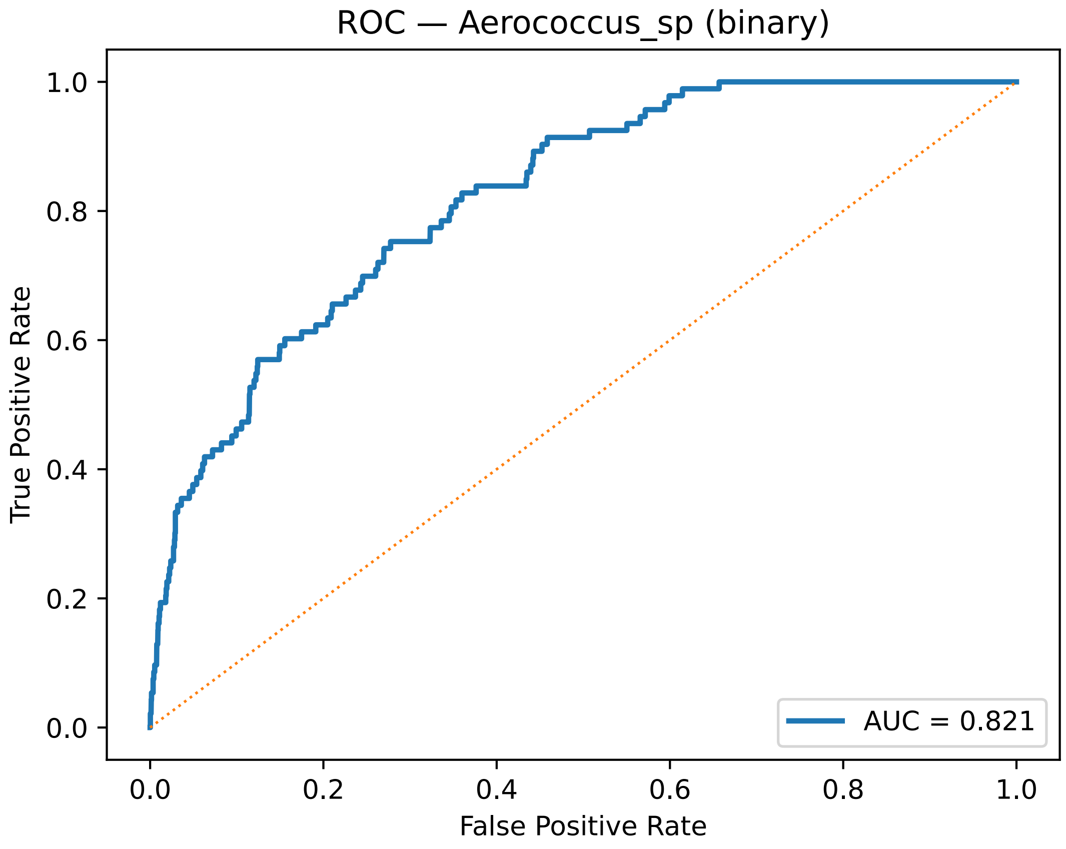
ROC — Aerococcus_sp (binary)
1.0
0.8
0.6
0.4
0.2
0.0
True Positive Rate
0.0
0.2
0.4
0.6
0.8
1.0
False Positive Rate
AUC = 0.821

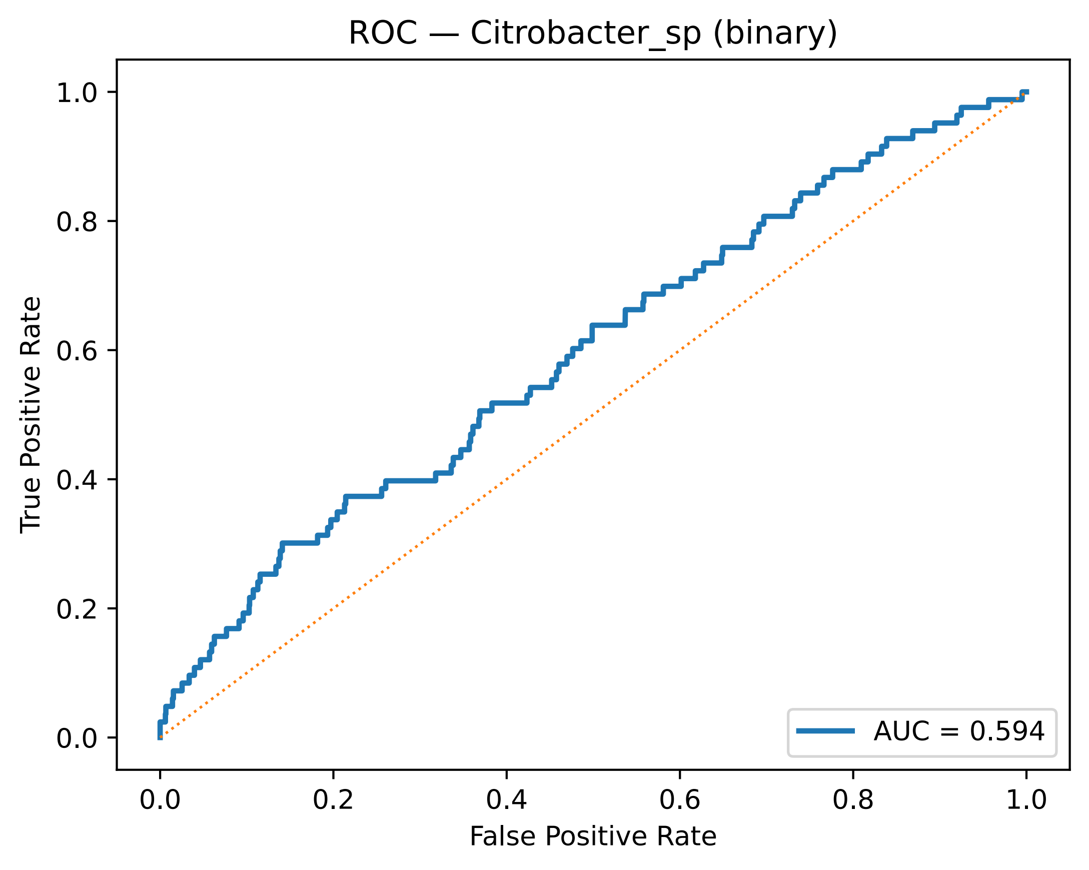
ROC — Citrobacter_sp (binary)
True Positive Rate
False Positive Rate
0.0
0.2
0.4
0.6
0.8
1.0
AUC = 0.594

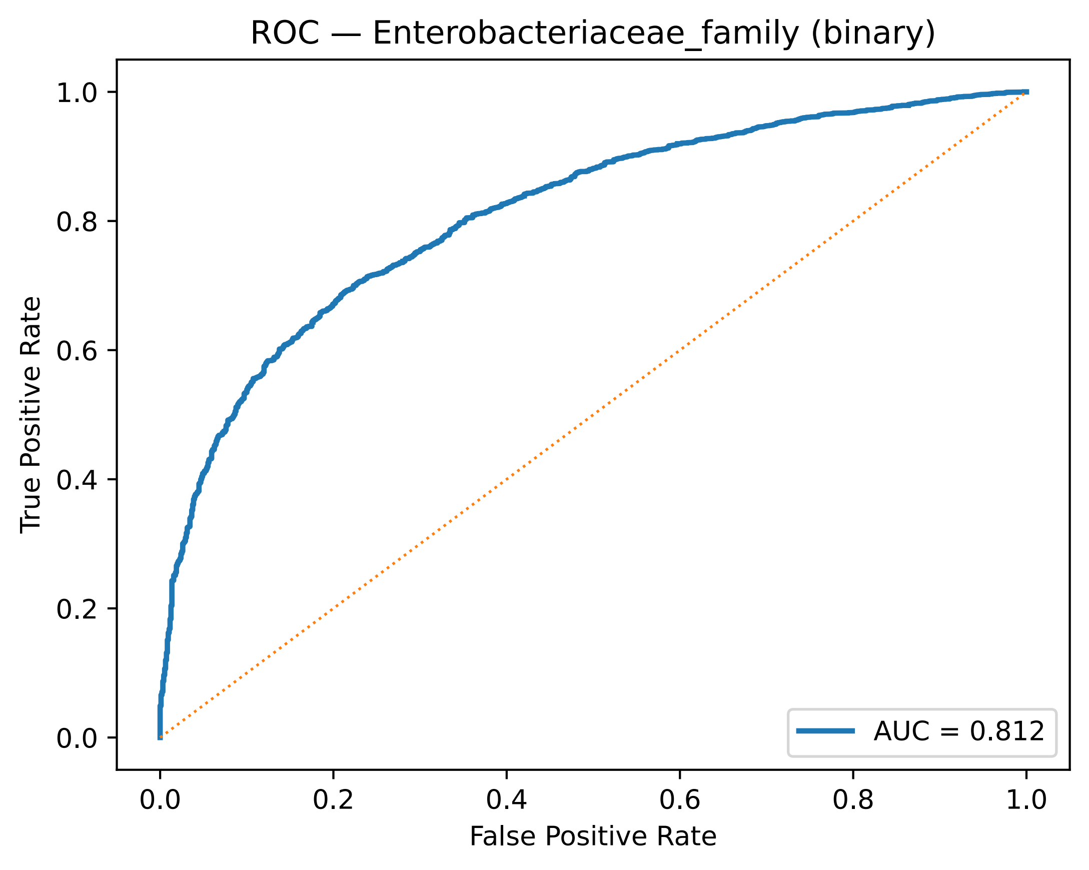
ROC — Enterobacteriaceae_family (binary)
True Positive Rate
False Positive Rate
AUC = 0.812
0.0
0.2
0.4
0.6
0.8
1.0

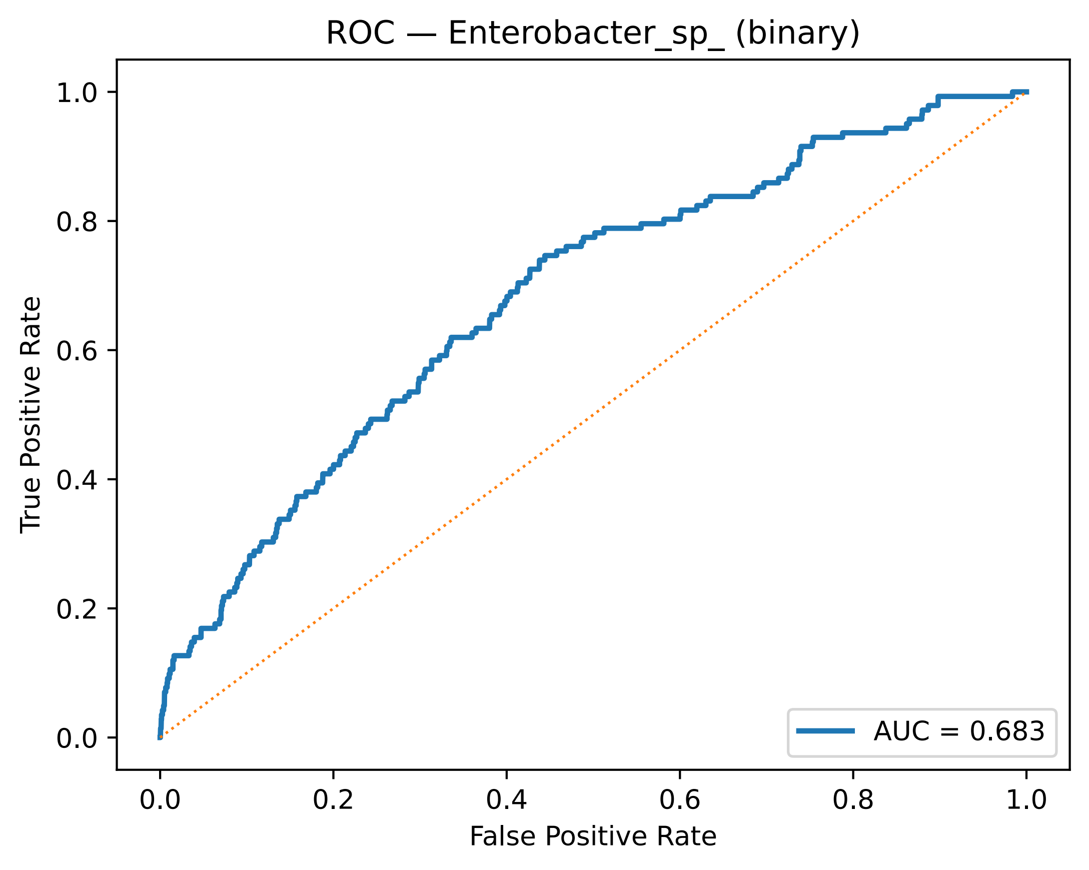
ROC — Enterobacter_sp_ (binary)
1.0
0.8
0.6
0.4
0.2
0.0
True Positive Rate
0.0
0.2
0.4
0.6
0.8
1.0
False Positive Rate
AUC = 0.683

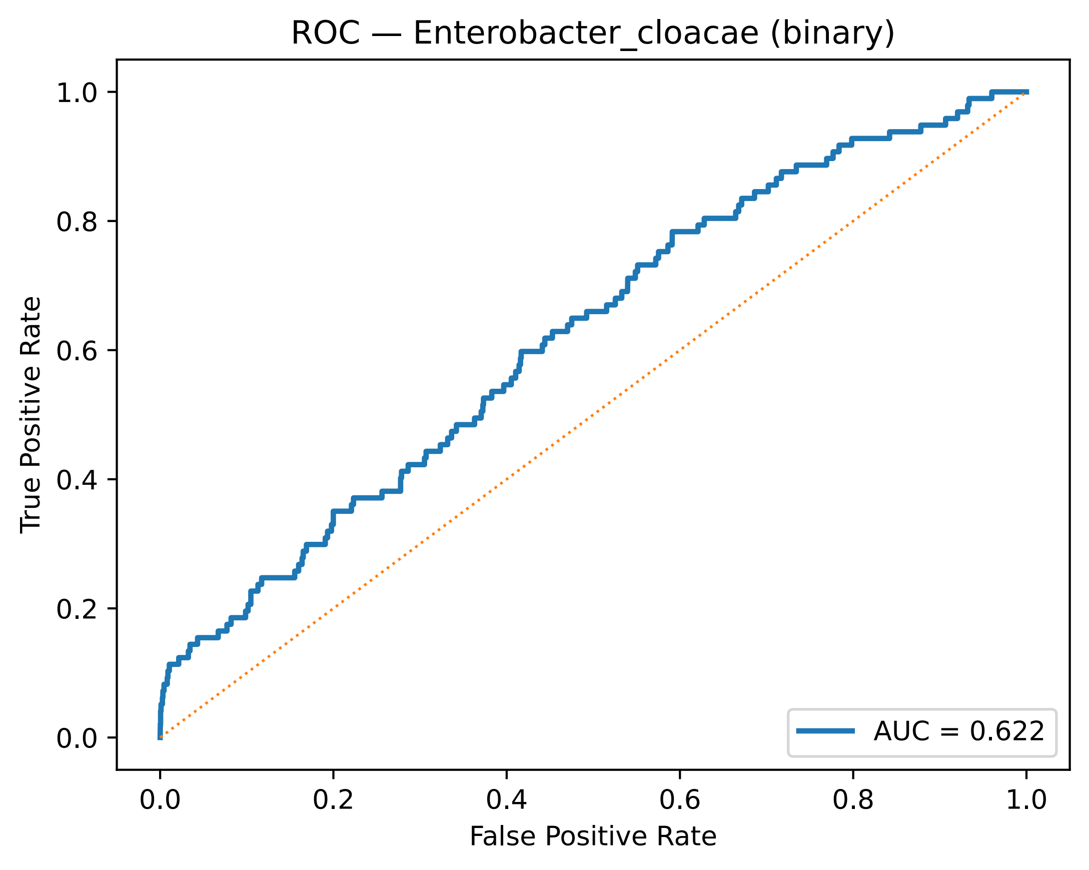
ROC — Enterobacter_cloacae (binary)
True Positive Rate
False Positive Rate
0.0
0.2
0.4
0.6
0.8
1.0
AUC = 0.622

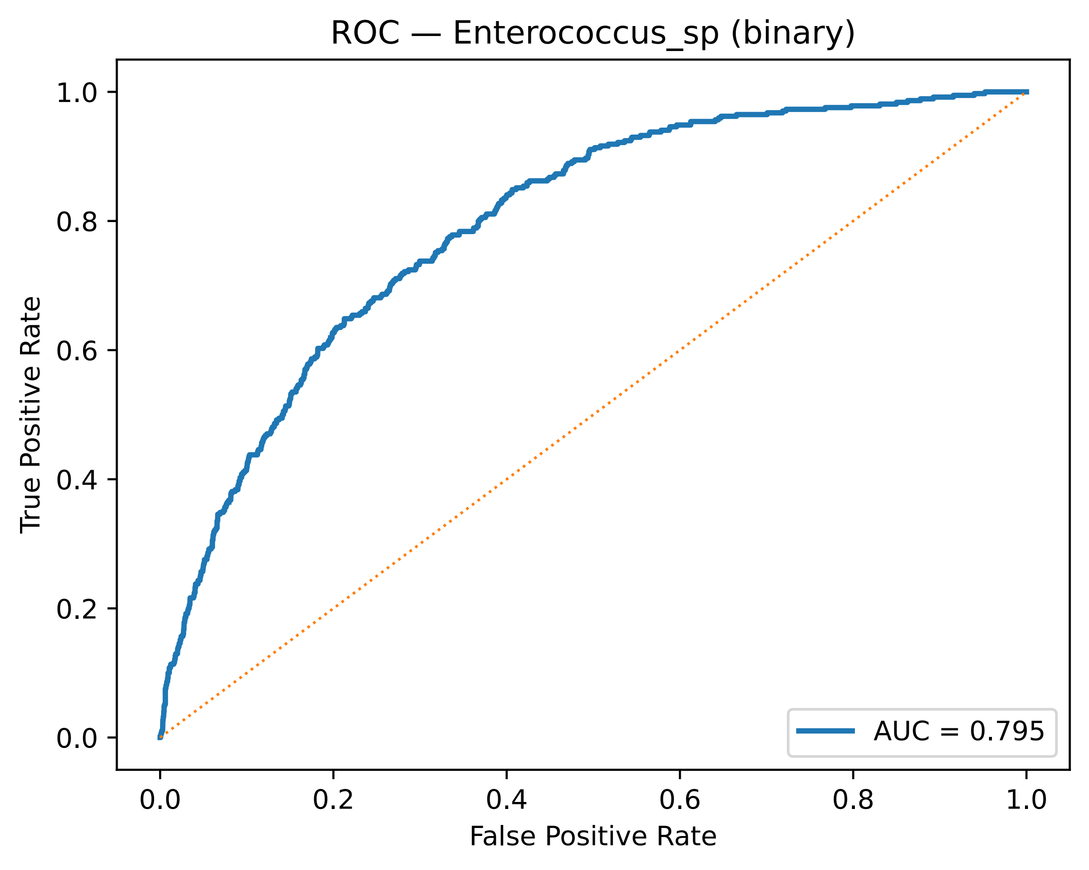

ROC — Enterococcus_sp (binary)
True Positive Rate
False Positive Rate
0.0
0.2
0.4
0.6
0.8
1.0
AUC = 0.795

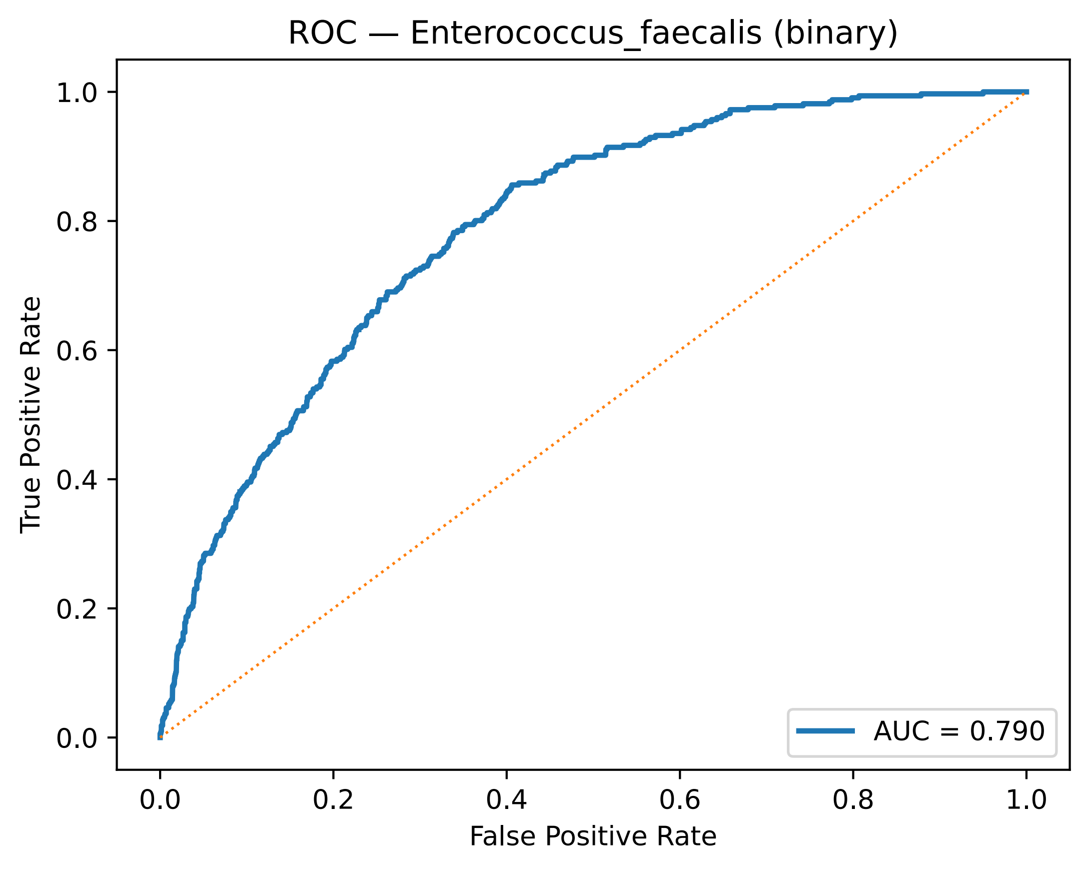
ROC — Enterococcus_faecalis (binary)
True Positive Rate
False Positive Rate
0.0
0.2
0.4
0.6
0.8
1.0
AUC = 0.790

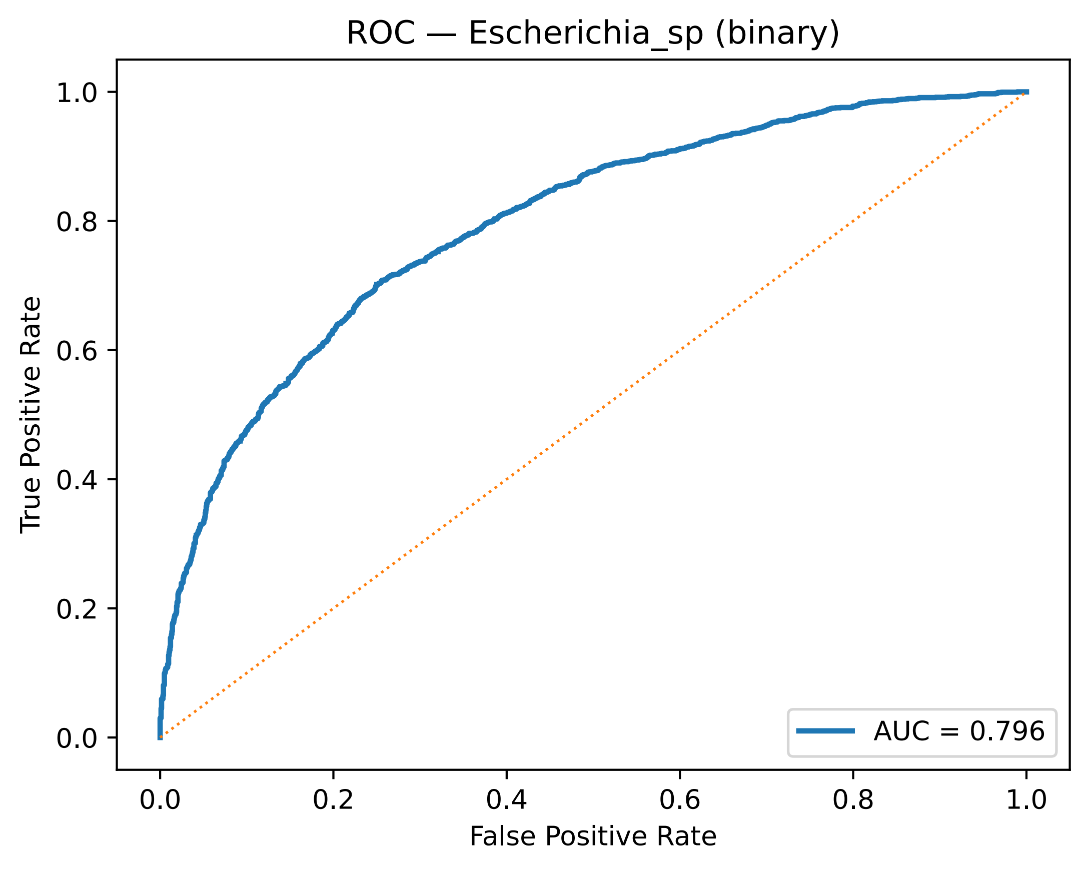
ROC — Escherichia_sp (binary)
True Positive Rate
False Positive Rate
0.0
0.2
0.4
0.6
0.8
1.0
AUC = 0.796

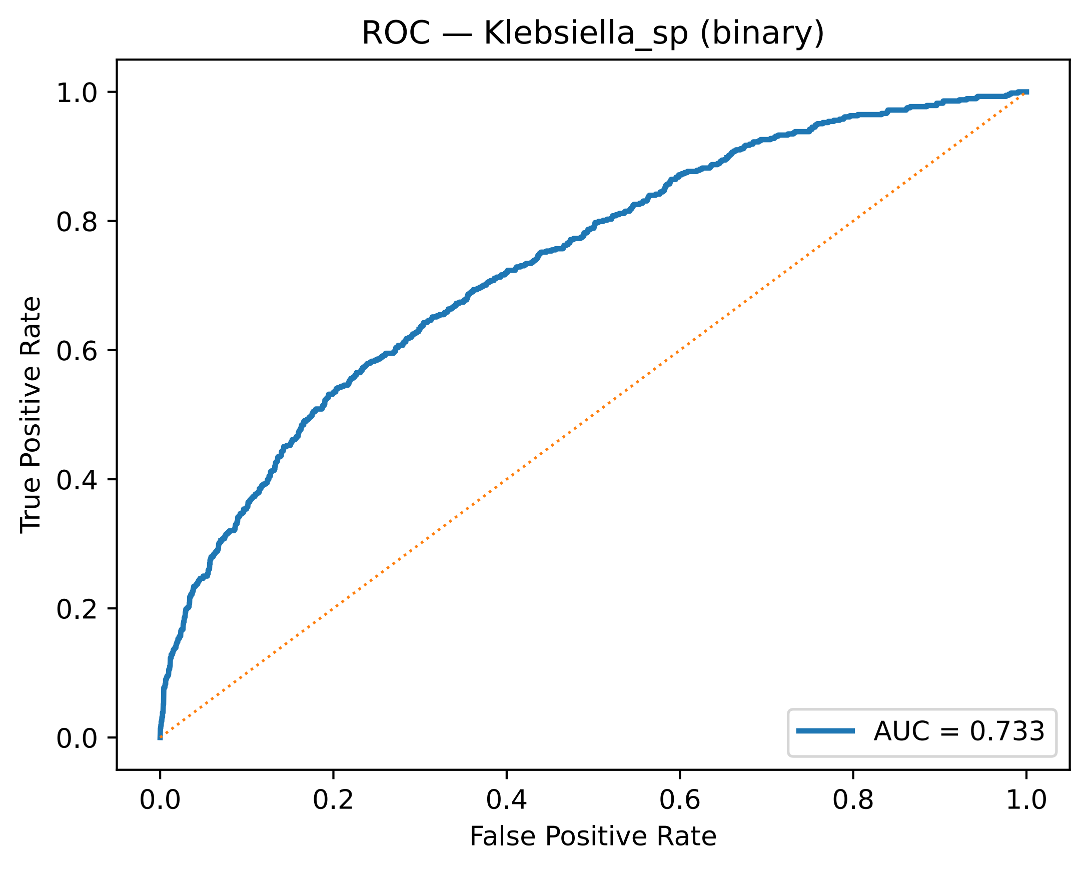

ROC — Klebsiella_sp (binary)
True Positive Rate
False Positive Rate
0.0
0.2
0.4
0.6
0.8
1.0
AUC = 0.733

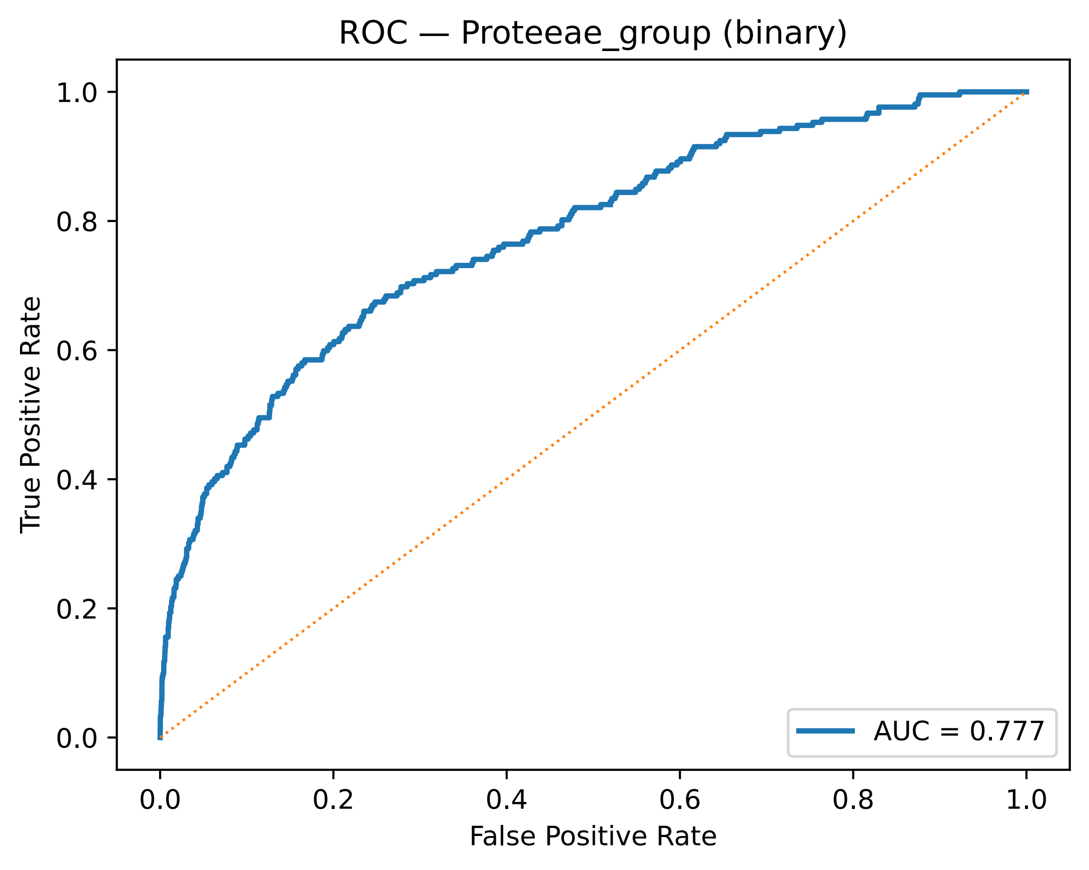
ROC — Proteeae_group (binary)
True Positive Rate
False Positive Rate
AUC = 0.777
0.0
0.2
0.4
0.6
0.8
1.0

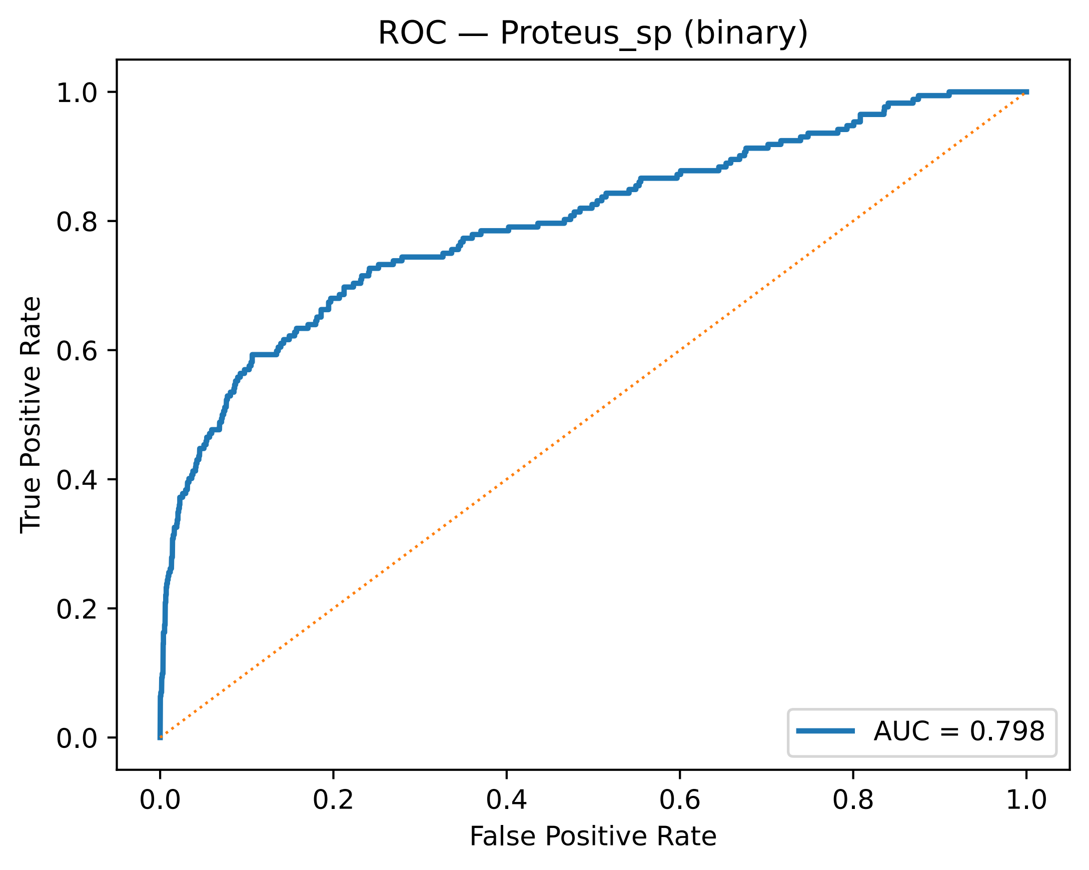
ROC — Proteus_sp (binary)
True Positive Rate
False Positive Rate
0.0
0.2
0.4
0.6
0.8
1.0
AUC = 0.798

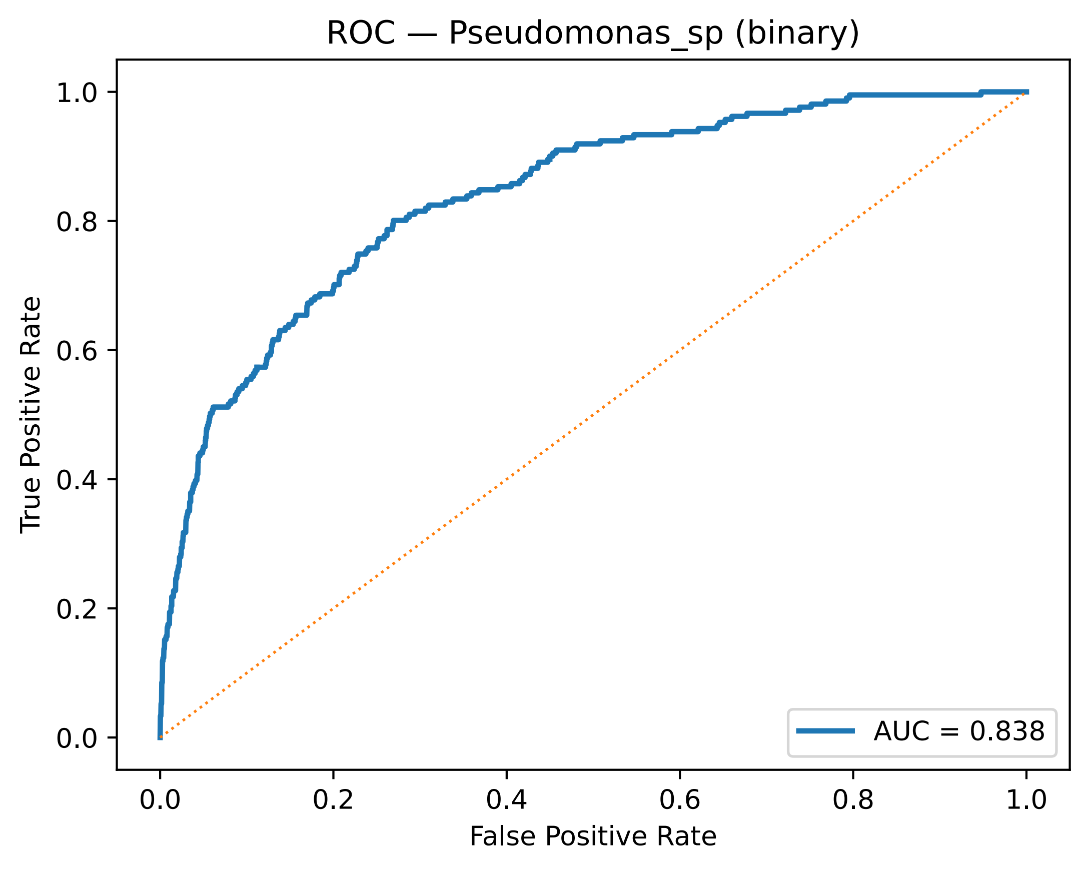

ROC — Pseudomonas_sp (binary)
True Positive Rate
False Positive Rate
0.0
0.2
0.4
0.6
0.8
1.0
AUC = 0.838

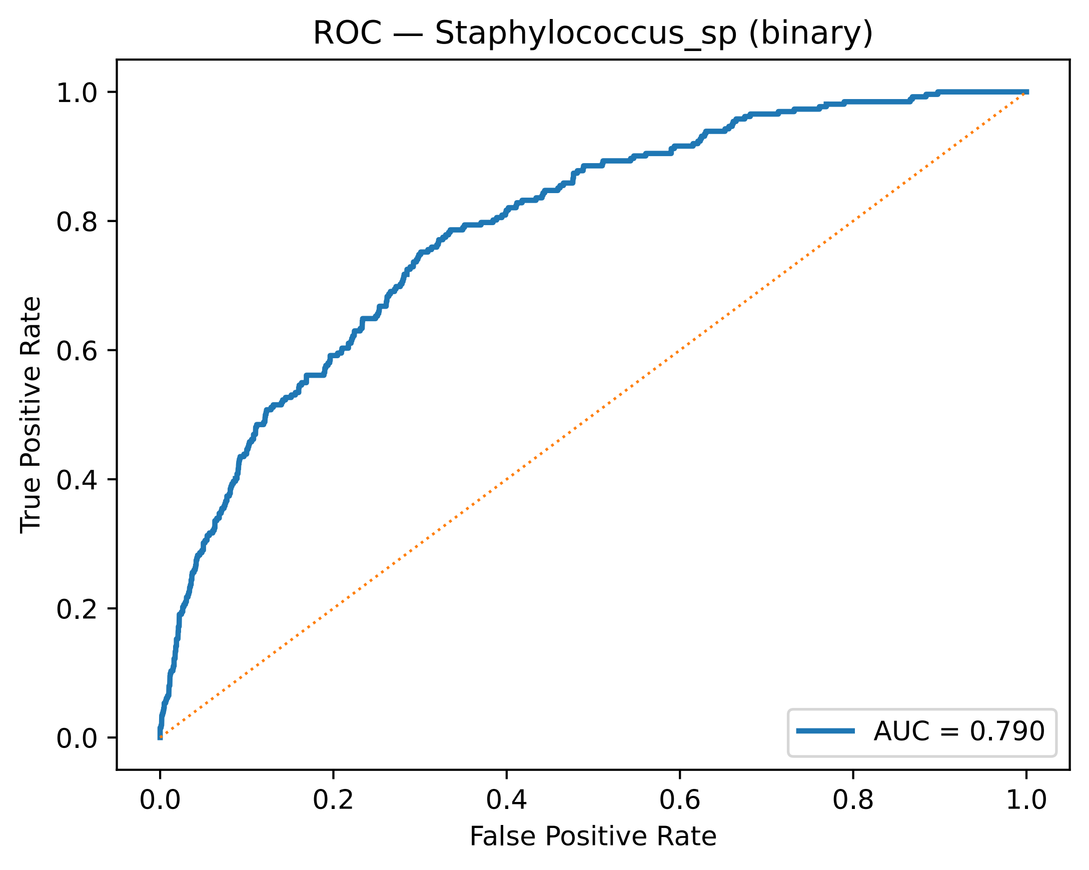
ROC — Staphylococcus_sp (binary)
True Positive Rate
False Positive Rate
0.0
0.2
0.4
0.6
0.8
1.0
AUC = 0.790

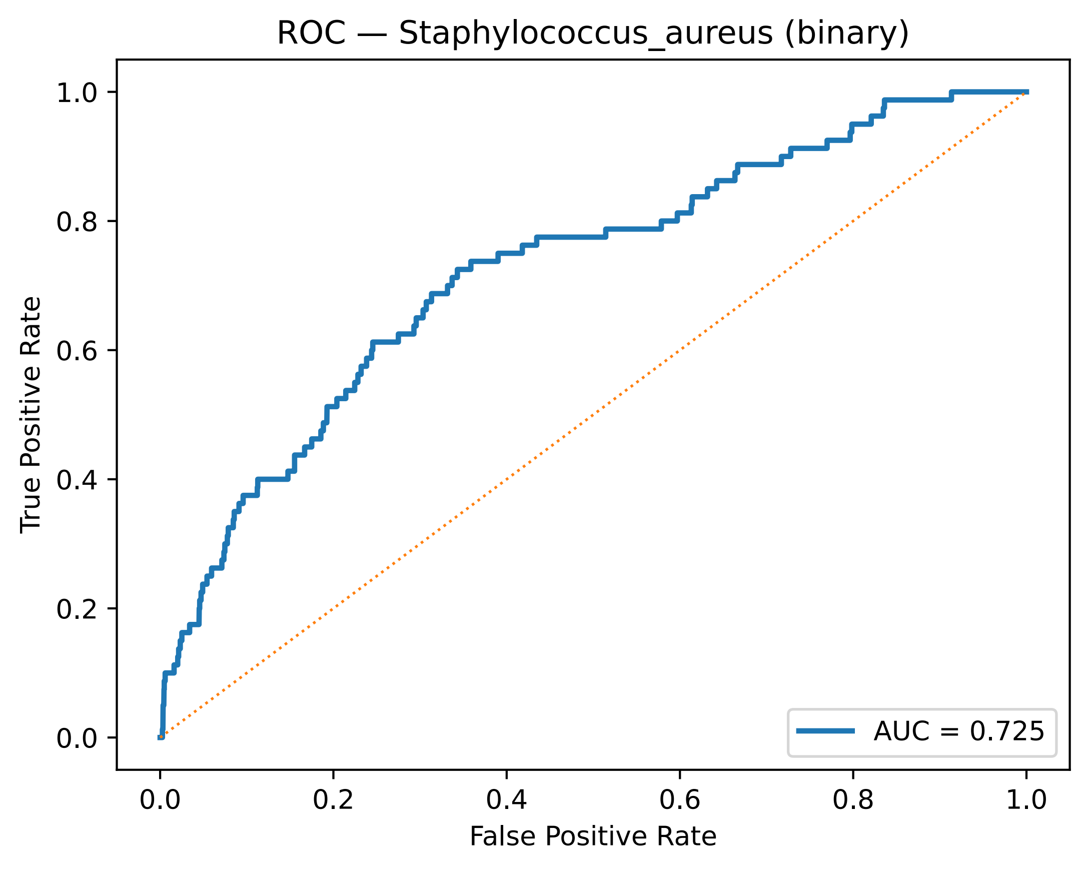
ROC — Staphylococcus_aureus (binary)
1.0
0.8
0.6
0.4
0.2
0.0
True Positive Rate
0.0
0.2
0.4
0.6
0.8
1.0
False Positive Rate
AUC = 0.725

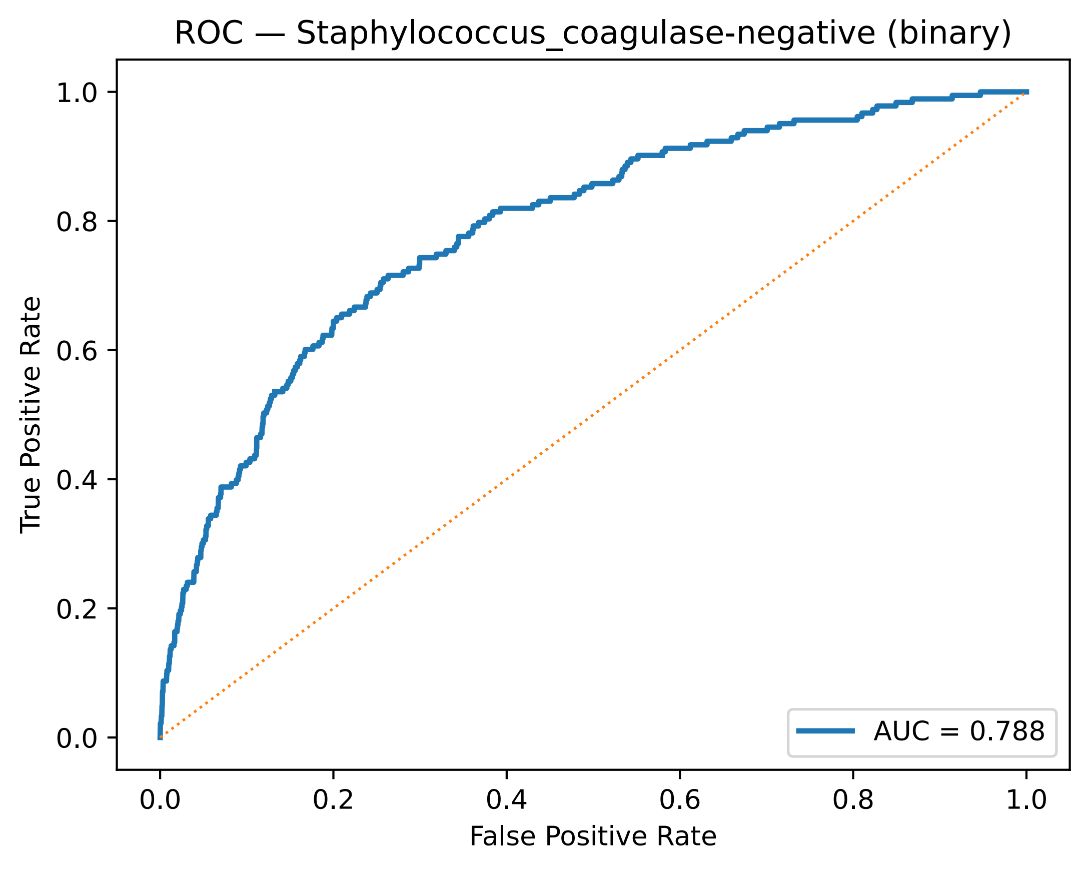
ROC — Staphylococcus_coagulase-negative (binary)
True Positive Rate
False Positive Rate
0.0
0.2
0.4
0.6
0.8
1.0
AUC = 0.788

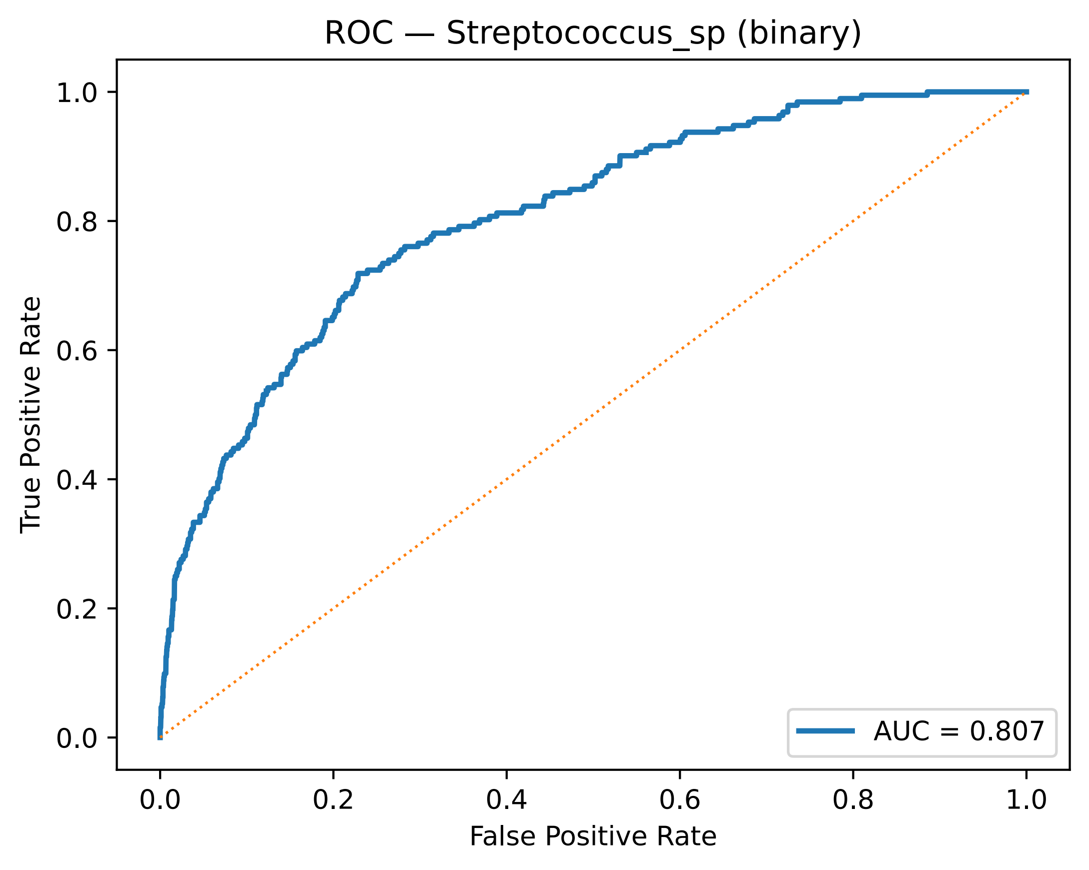
ROC — Streptococcus_sp (binary)
True Positive Rate
False Positive Rate
0.0
0.2
0.4
0.6
0.8
1.0
AUC = 0.807

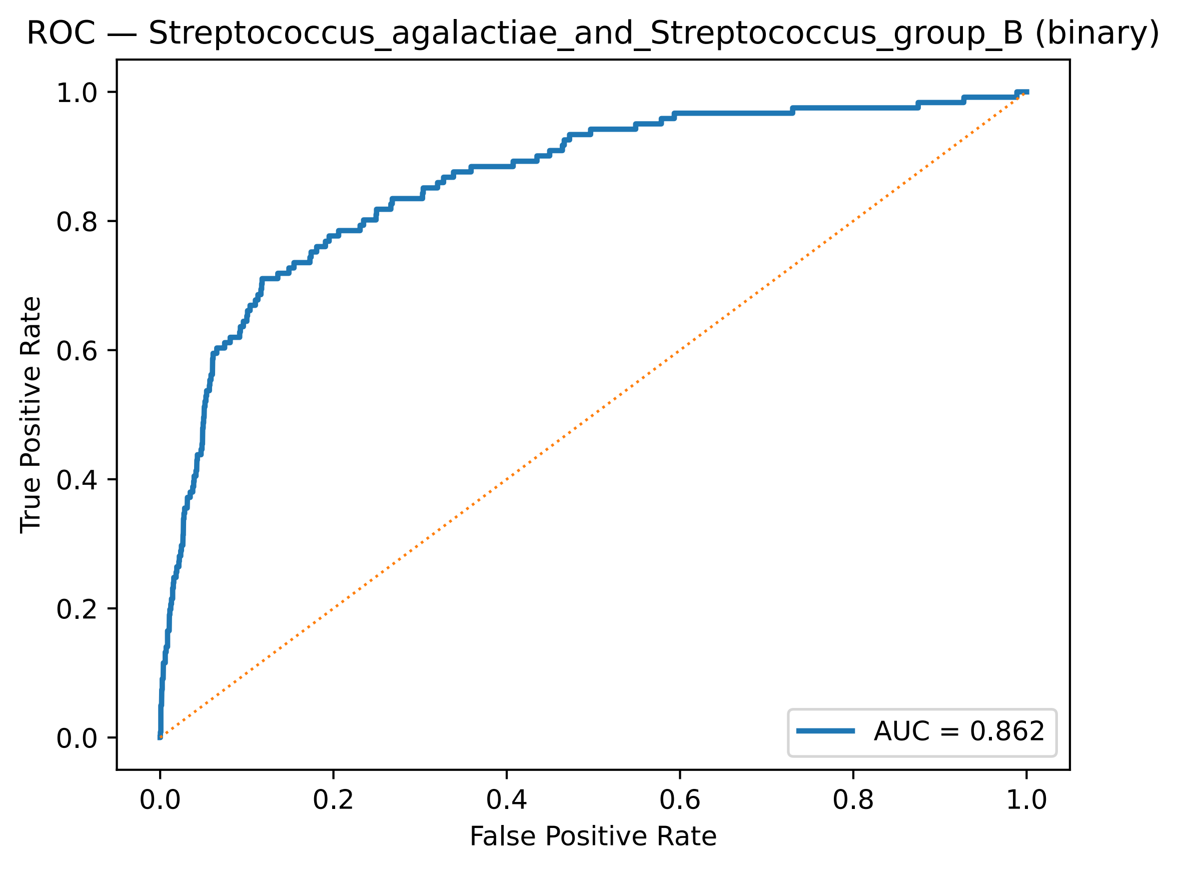

ROC — Streptococcus_agalactiae_and_Streptococcus_group_B (binary)
True Positive Rate
False Positive Rate
0.0
0.2
0.4
0.6
0.8
1.0
AUC = 0.862

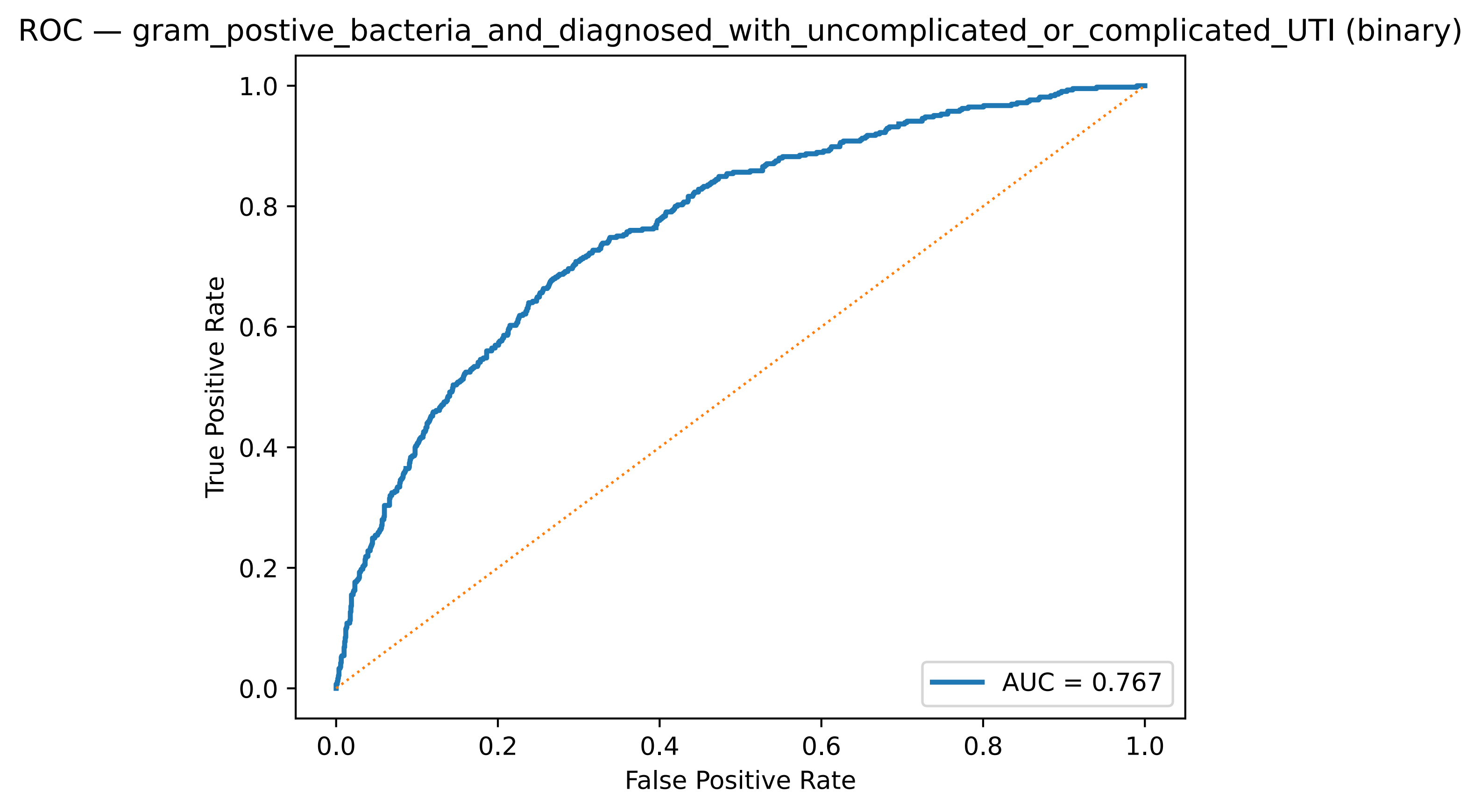
ROC — gram_postive_bacteria_and_diagnosed_with_uncomplicated_or_complicated_UTI (binary)
True Positive Rate
False Positive Rate
0.0
0.2
0.4
0.6
0.8
1.0
AUC = 0.767

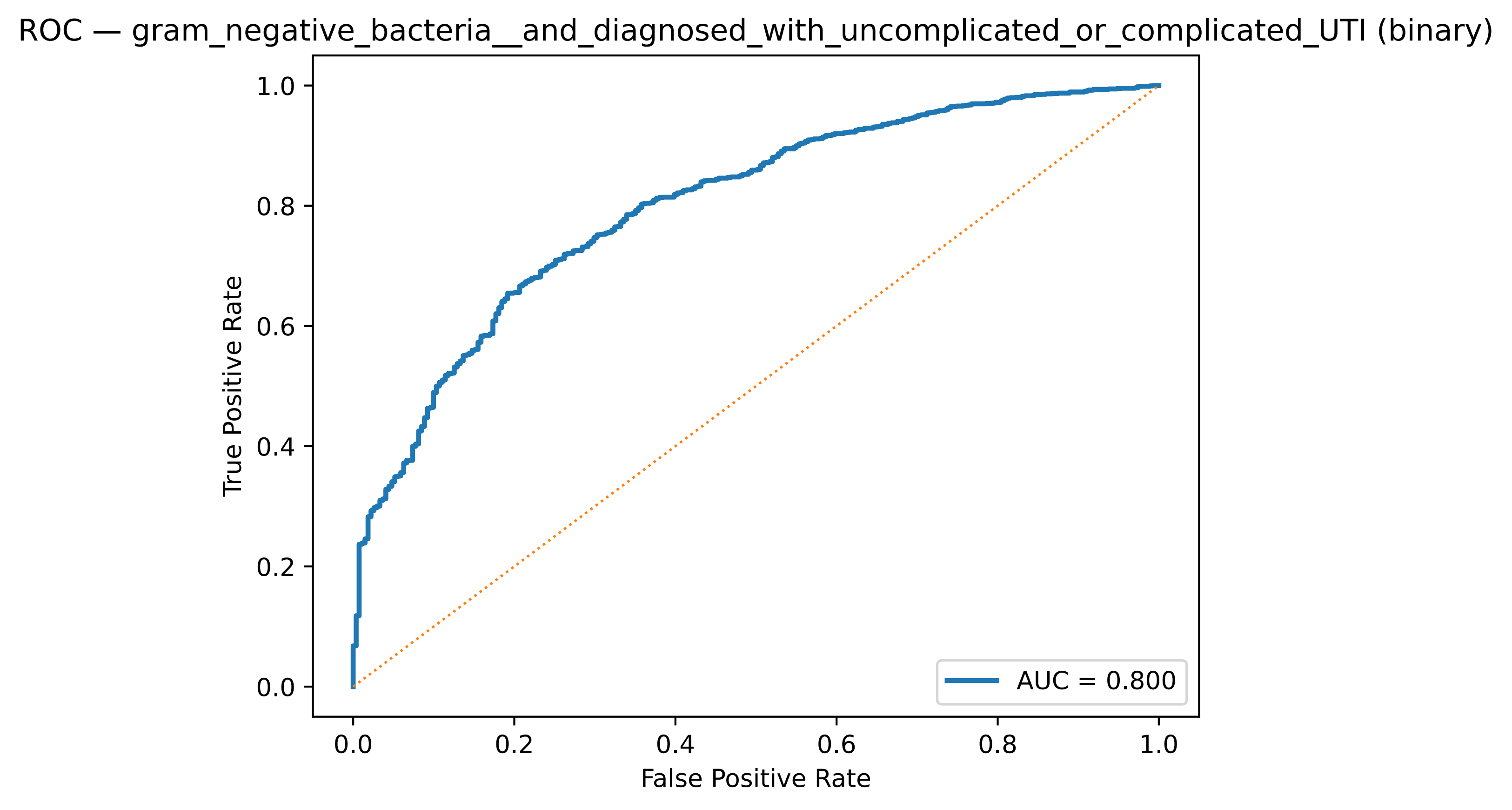

ROC — gram_negative_bacteria__and_diagnosed_with_uncomplicated_or_complicated_UTI (binary)
True Positive Rate
False Positive Rate
AUC = 0.800
0.0
0.2
0.4
0.6
0.8
1.0

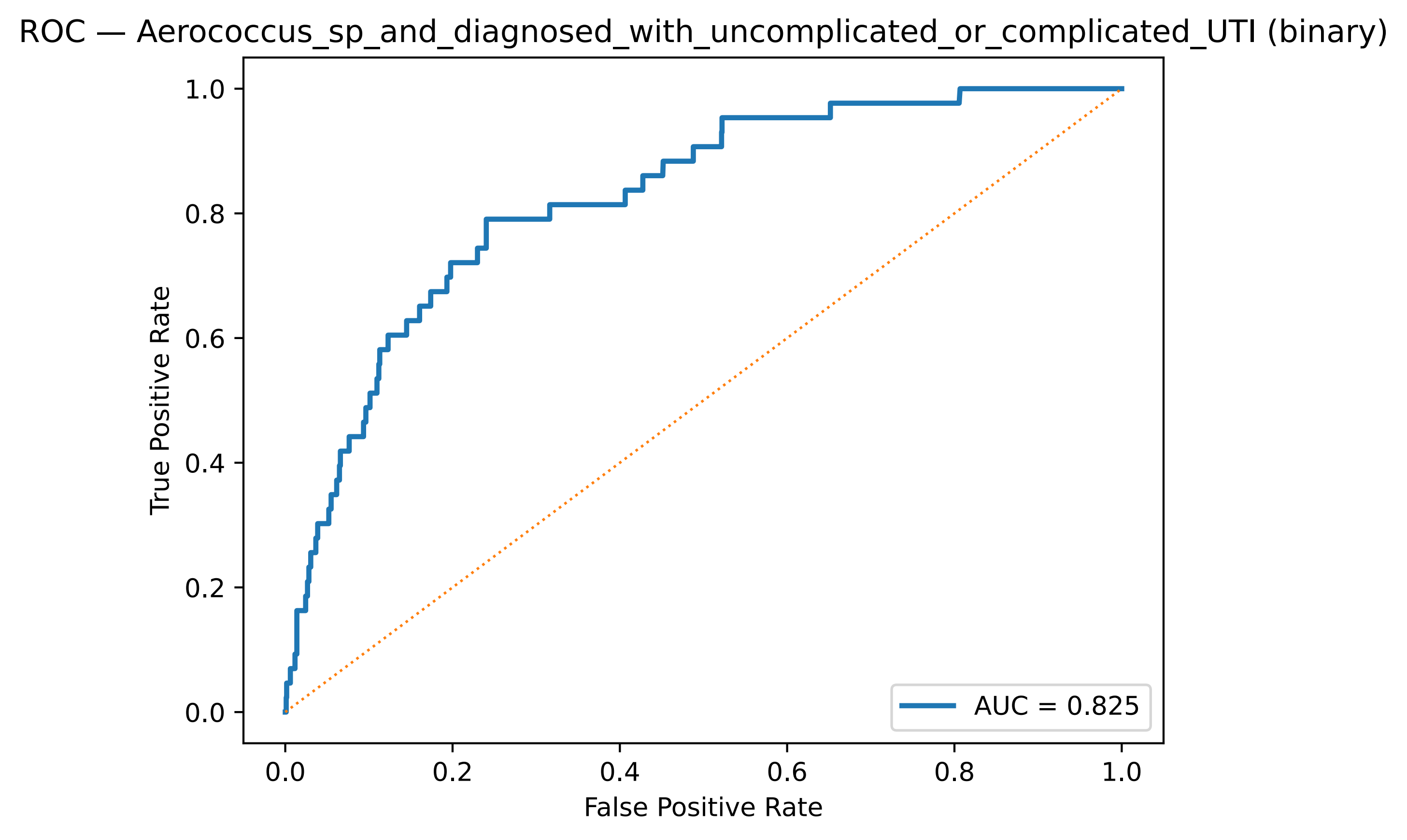

ROC — Aerococcus_sp_and_diagnosed_with_uncomplicated_or_complicated_UTI (binary)
True Positive Rate
False Positive Rate
0.0
0.2
0.4
0.6
0.8
1.0
AUC = 0.825

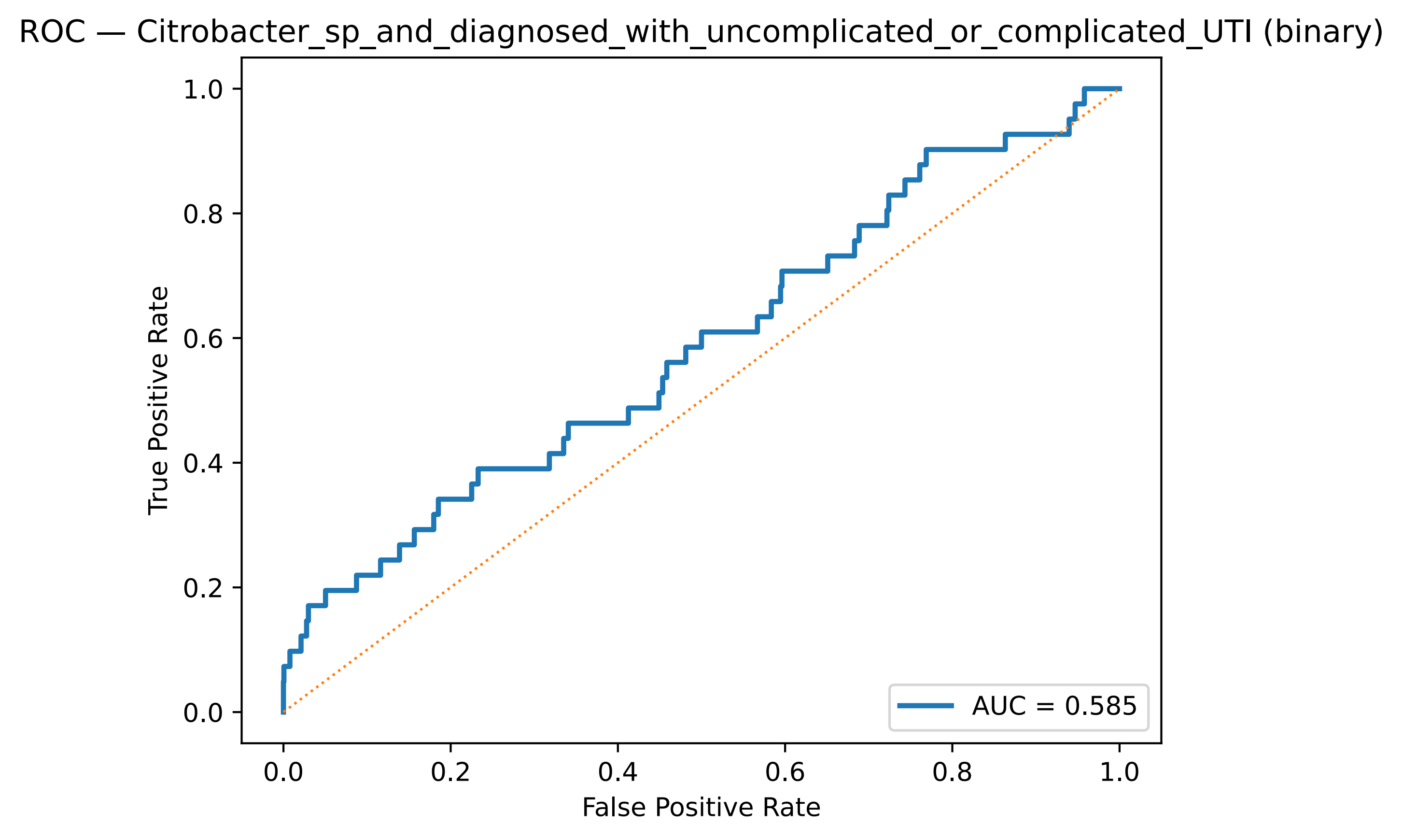

ROC — Citrobacter_sp_and_diagnosed_with_uncomplicated_or_complicated_UTI (binary)
True Positive Rate
False Positive Rate
0.0
0.2
0.4
0.6
0.8
1.0
AUC = 0.585

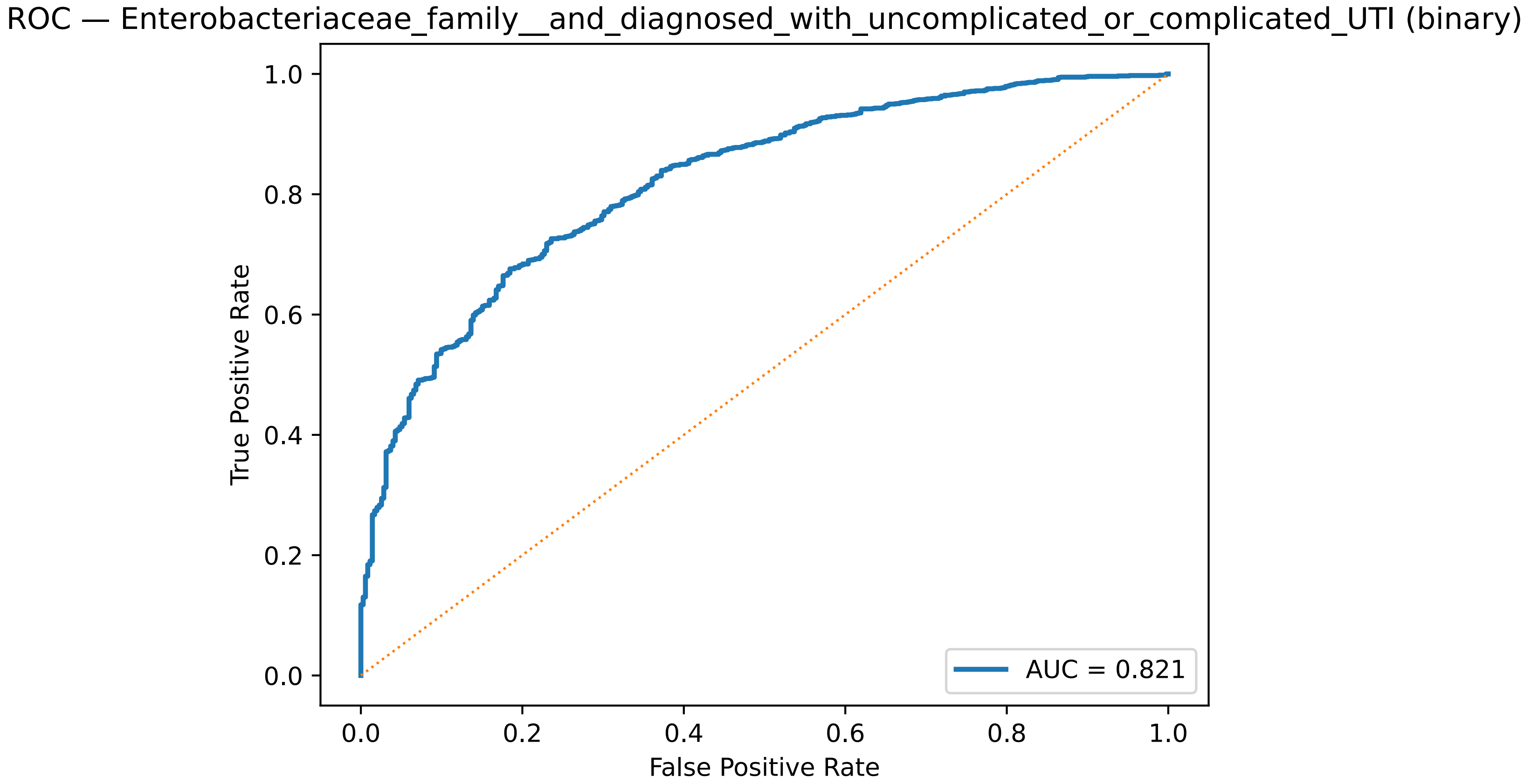

ROC — Enterobacteriaceae_family__and_diagnosed_with_uncomplicated_or_complicated_UTI (binary)
True Positive Rate
False Positive Rate
0.0
0.2
0.4
0.6
0.8
1.0
AUC = 0.821

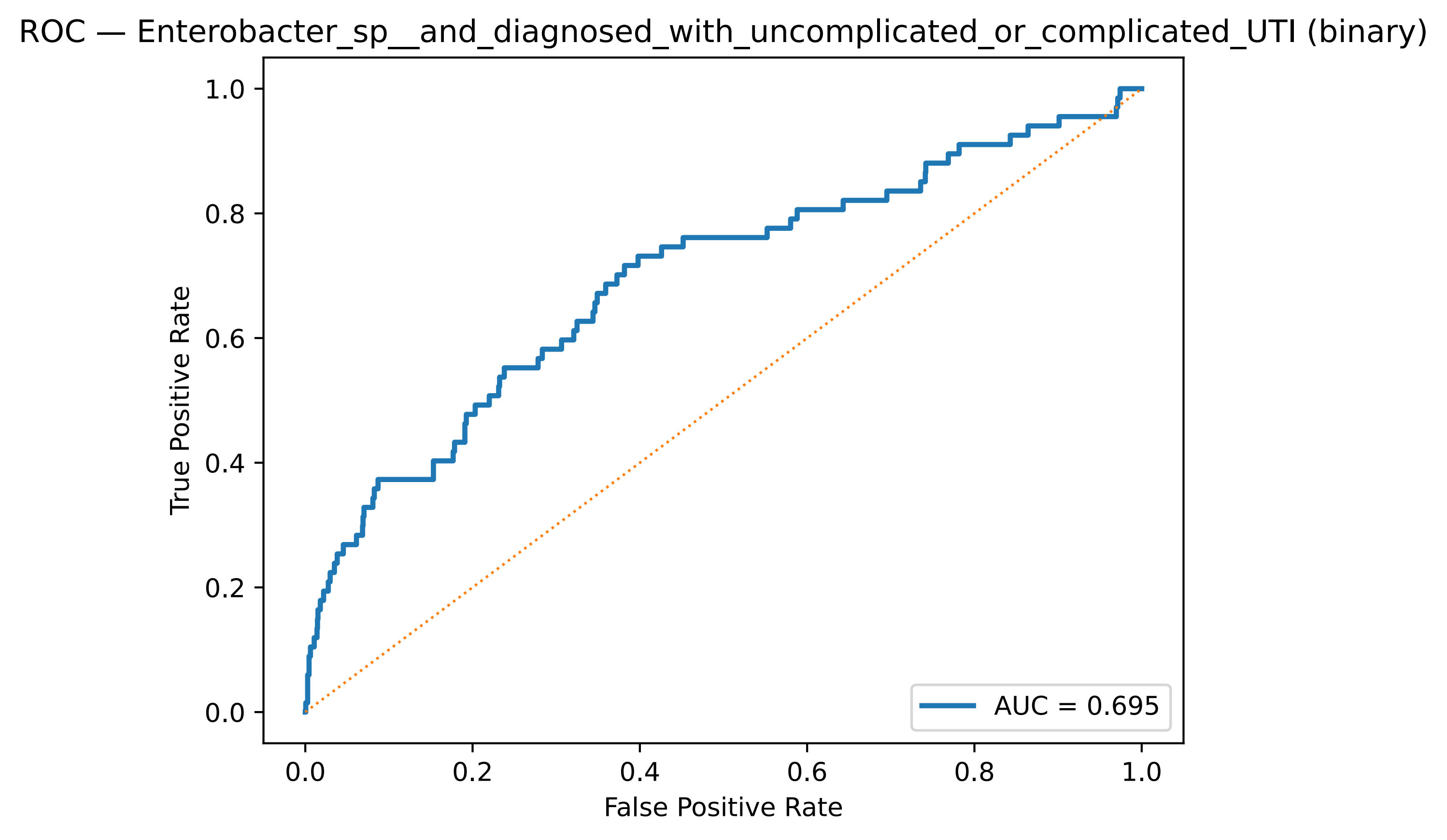

ROC — Enterobacter_sp__and_diagnosed_with_uncomplicated_or_complicated_UTI (binary)
True Positive Rate
False Positive Rate
0.0
0.2
0.4
0.6
0.8
1.0
AUC = 0.695

ROC — Enterobacter_cloacae_and_diagnosed_with_uncomplicated_or_complicated_UTI (binary)

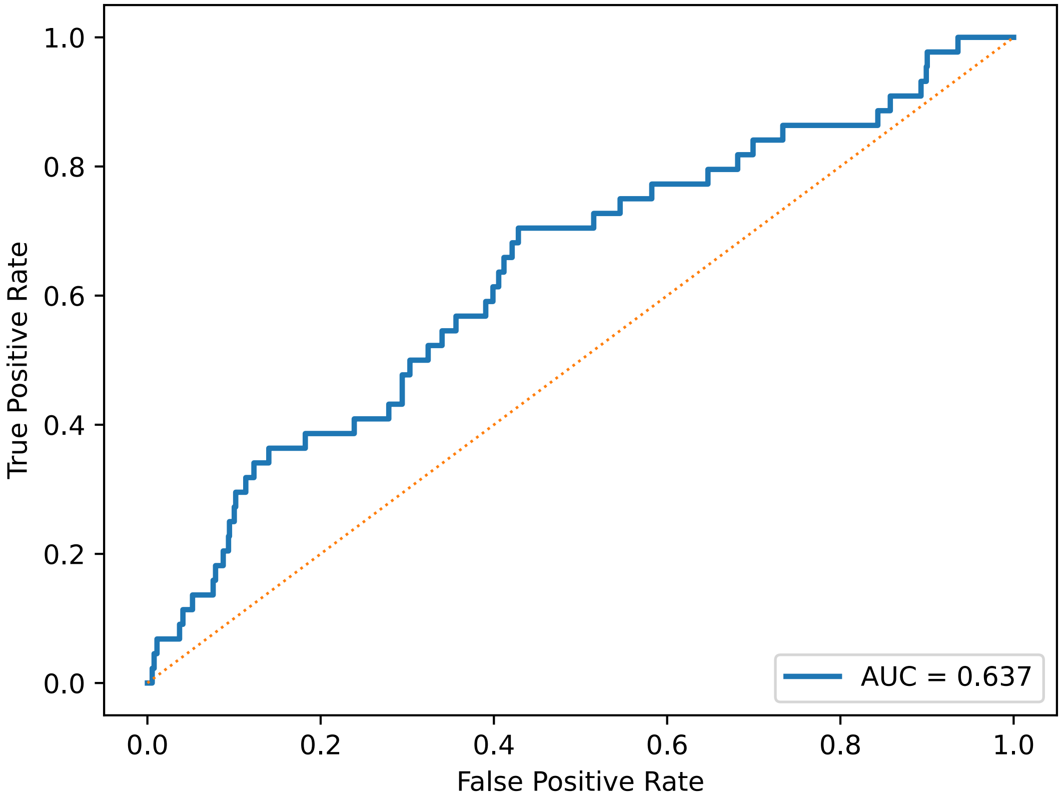

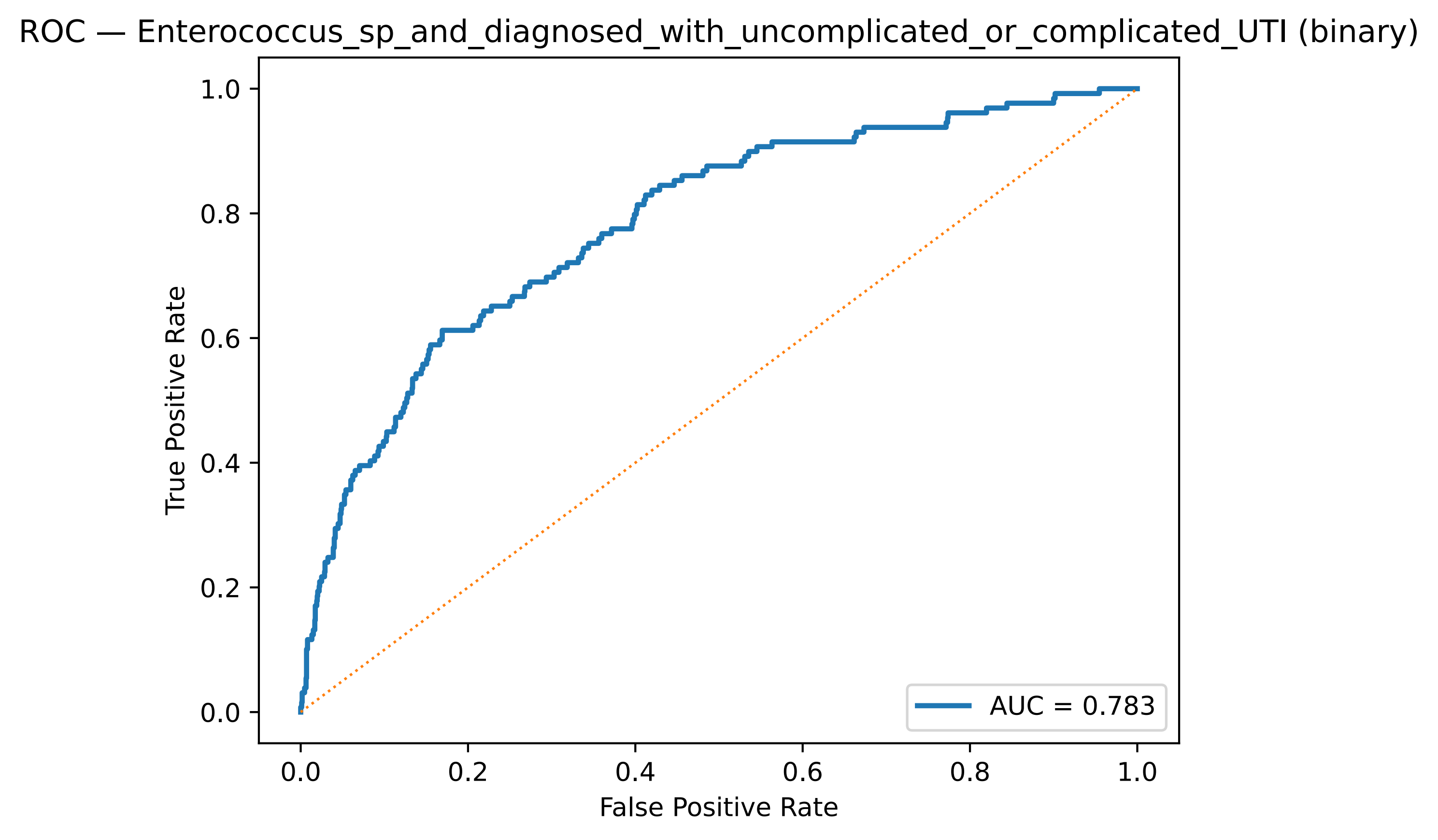
ROC — Enterococcus_sp_and_diagnosed_with_uncomplicated_or_complicated_UTI (binary)
True Positive Rate
False Positive Rate
0.0
0.2
0.4
0.6
0.8
1.0
AUC = 0.783

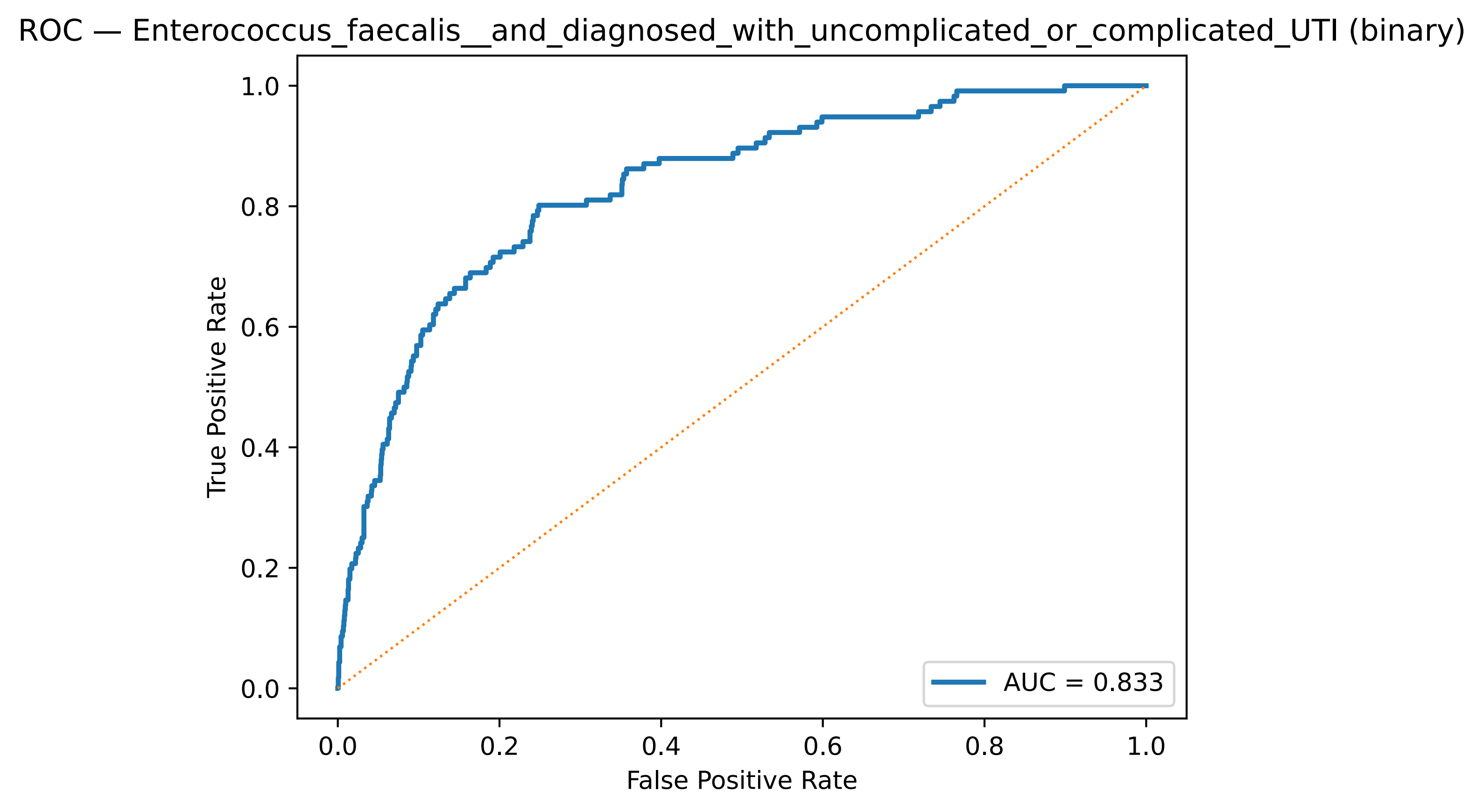
ROC — Enterococcus_faecalis__and_diagnosed_with_uncomplicated_or_complicated_UTI (binary)
1.0
0.8
0.6
0.4
0.2
0.0
True Positive Rate
0.0
0.2
0.4
0.6
0.8
1.0
False Positive Rate
AUC = 0.833

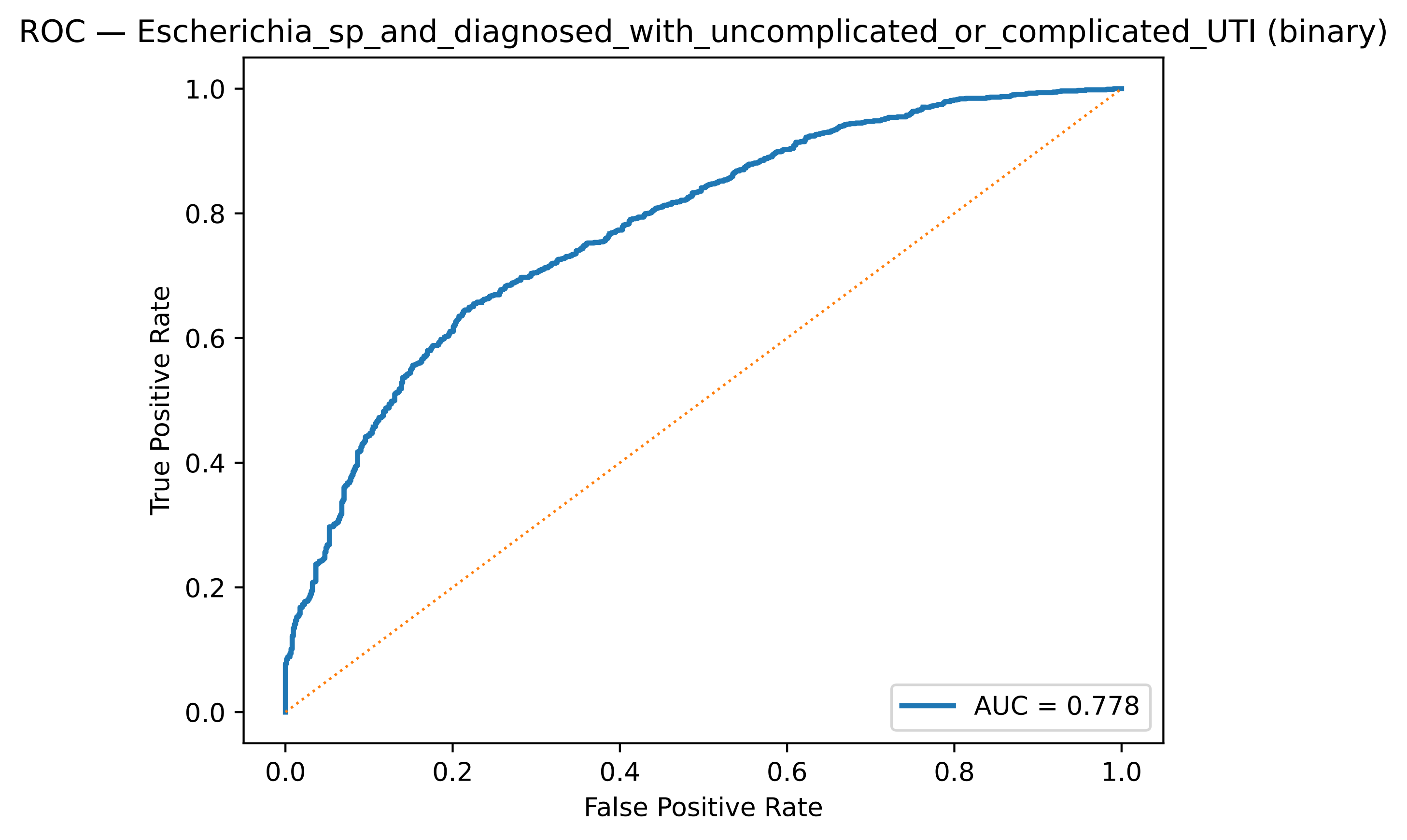

ROC — Escherichia_sp_and_diagnosed_with_uncomplicated_or_complicated_UTI (binary)
True Positive Rate
False Positive Rate
0.0
0.2
0.4
0.6
0.8
1.0
AUC = 0.778

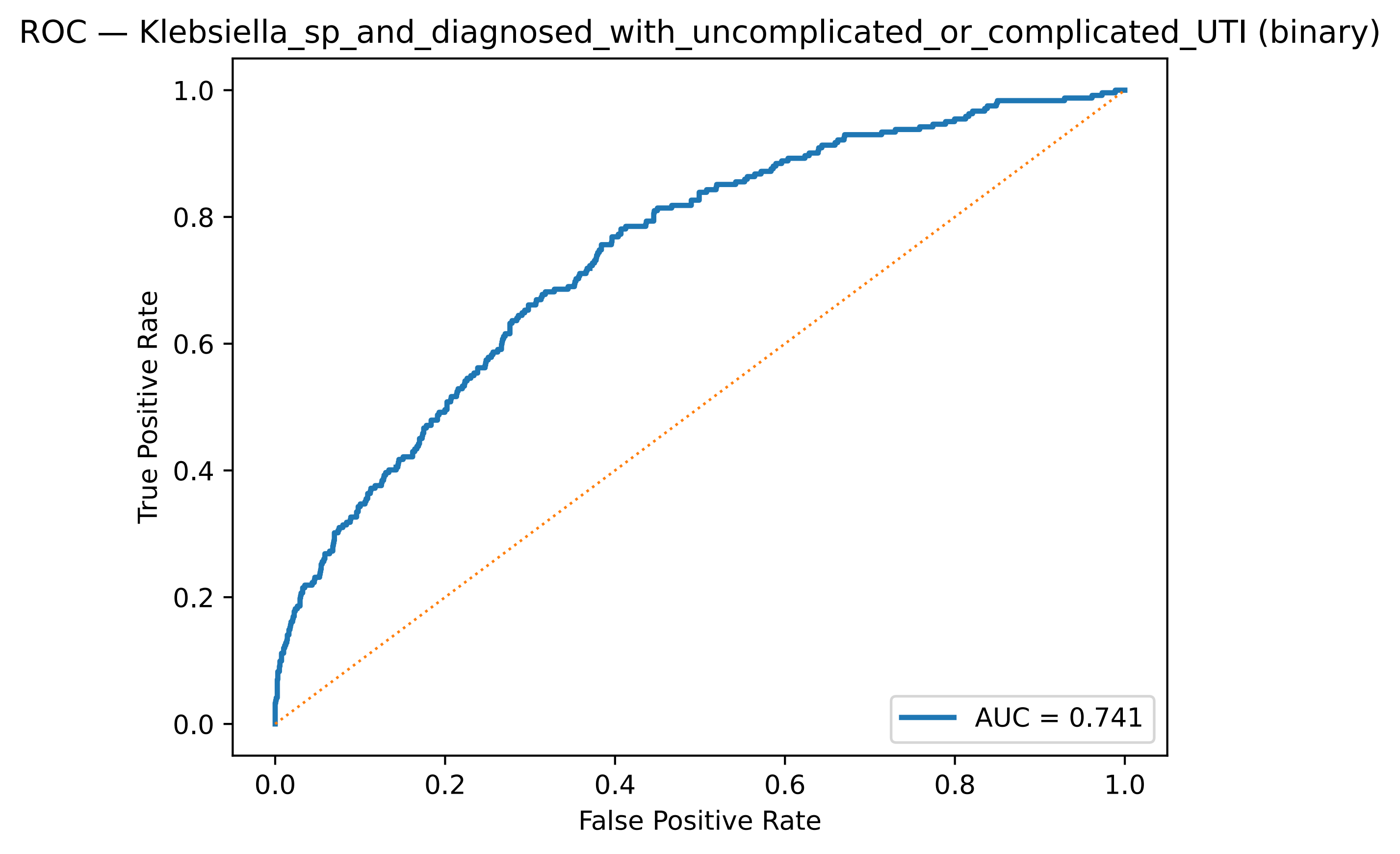
ROC — Klebsiella_sp_and_diagnosed_with_uncomplicated_or_complicated_UTI (binary)
True Positive Rate
False Positive Rate
AUC = 0.741
0.0
0.2
0.4
0.6
0.8
1.0

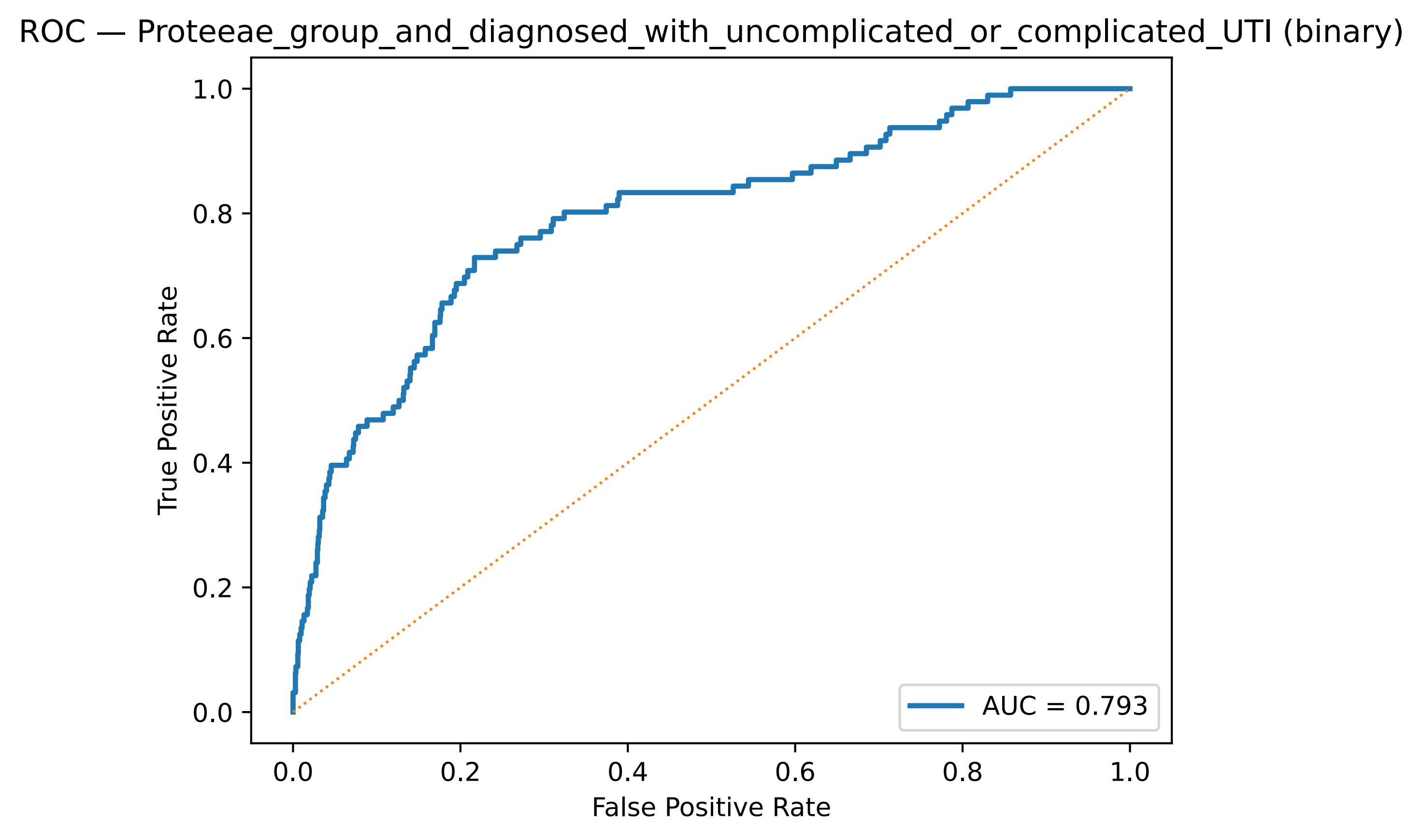
ROC — Proteeae_group_and_diagnosed_with_uncomplicated_or_complicated_UTI (binary)
True Positive Rate
False Positive Rate
0.0
0.2
0.4
0.6
0.8
1.0
AUC = 0.793

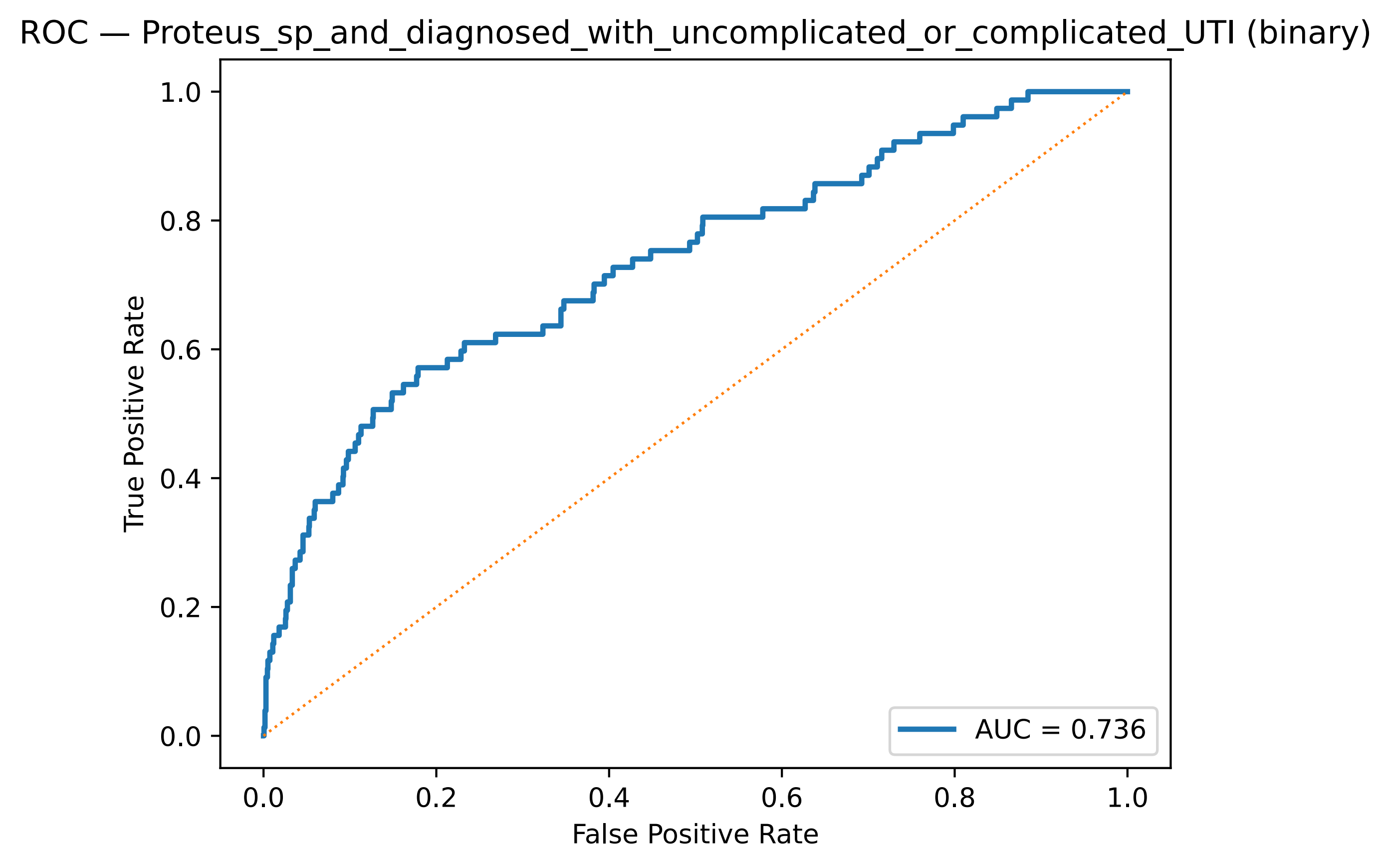
ROC — Proteus_sp_and_diagnosed_with_uncomplicated_or_complicated_UTI (binary)
True Positive Rate
False Positive Rate
0.0
0.2
0.4
0.6
0.8
1.0
AUC = 0.736

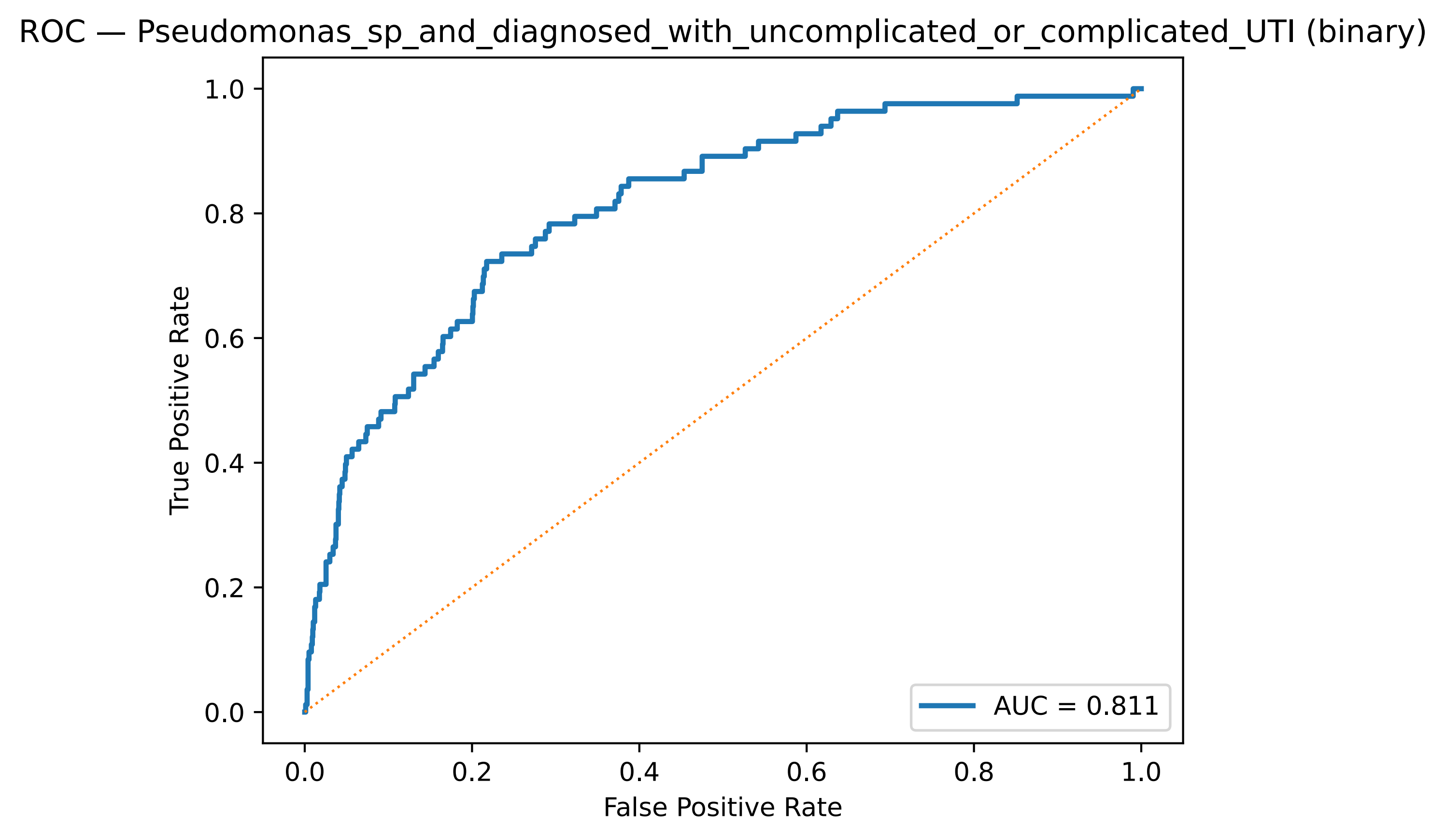

ROC — Pseudomonas_sp_and_diagnosed_with_uncomplicated_or_complicated_UTI (binary)
True Positive Rate
False Positive Rate
0.0
0.2
0.4
0.6
0.8
1.0
AUC = 0.811

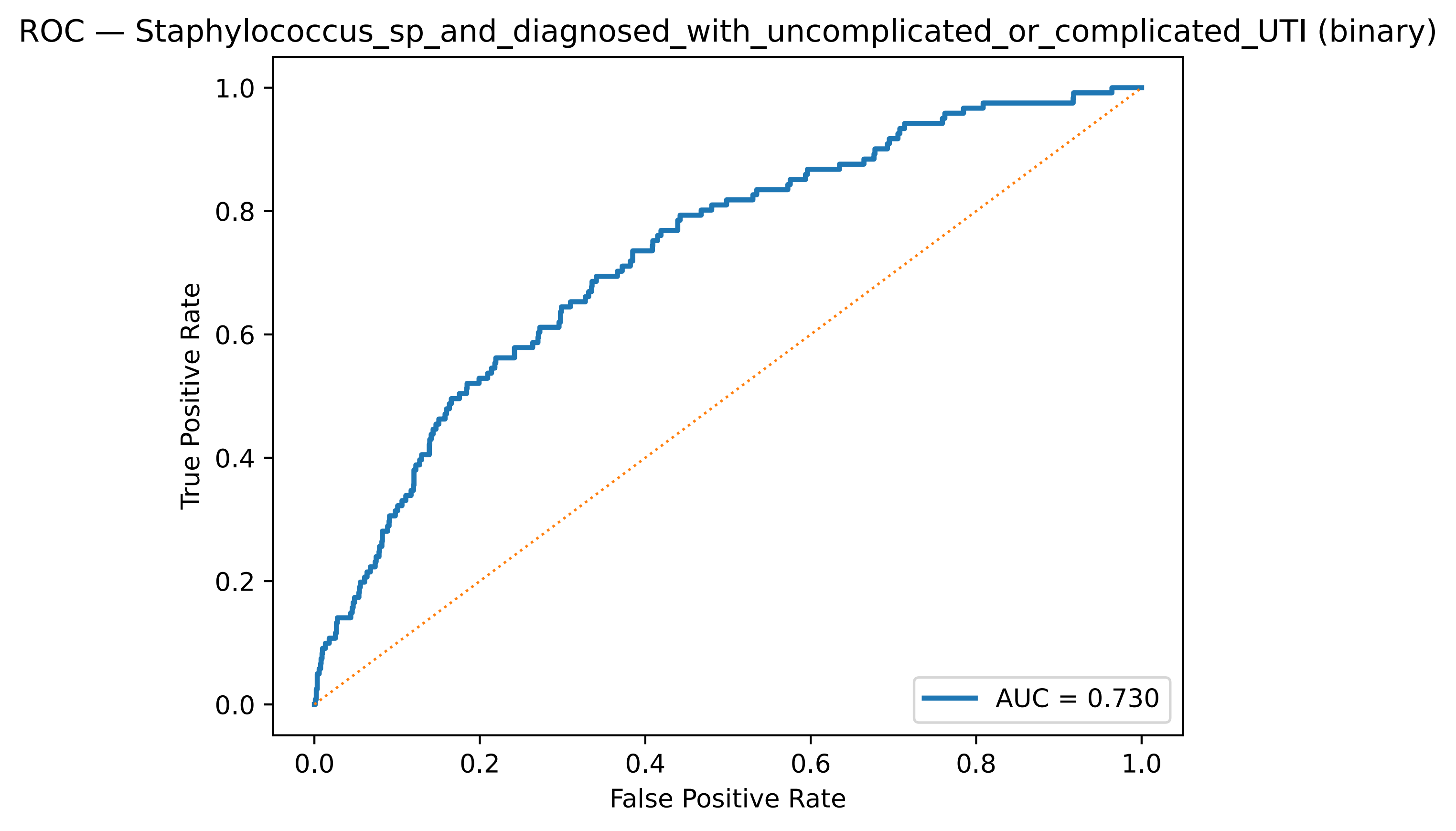
ROC — Staphylococcus_sp_and_diagnosed_with_uncomplicated_or_complicated_UTI (binary)
True Positive Rate
False Positive Rate
0.0
0.2
0.4
0.6
0.8
1.0
0.0
0.2
0.4
0.6
0.8
1.0
AUC = 0.730

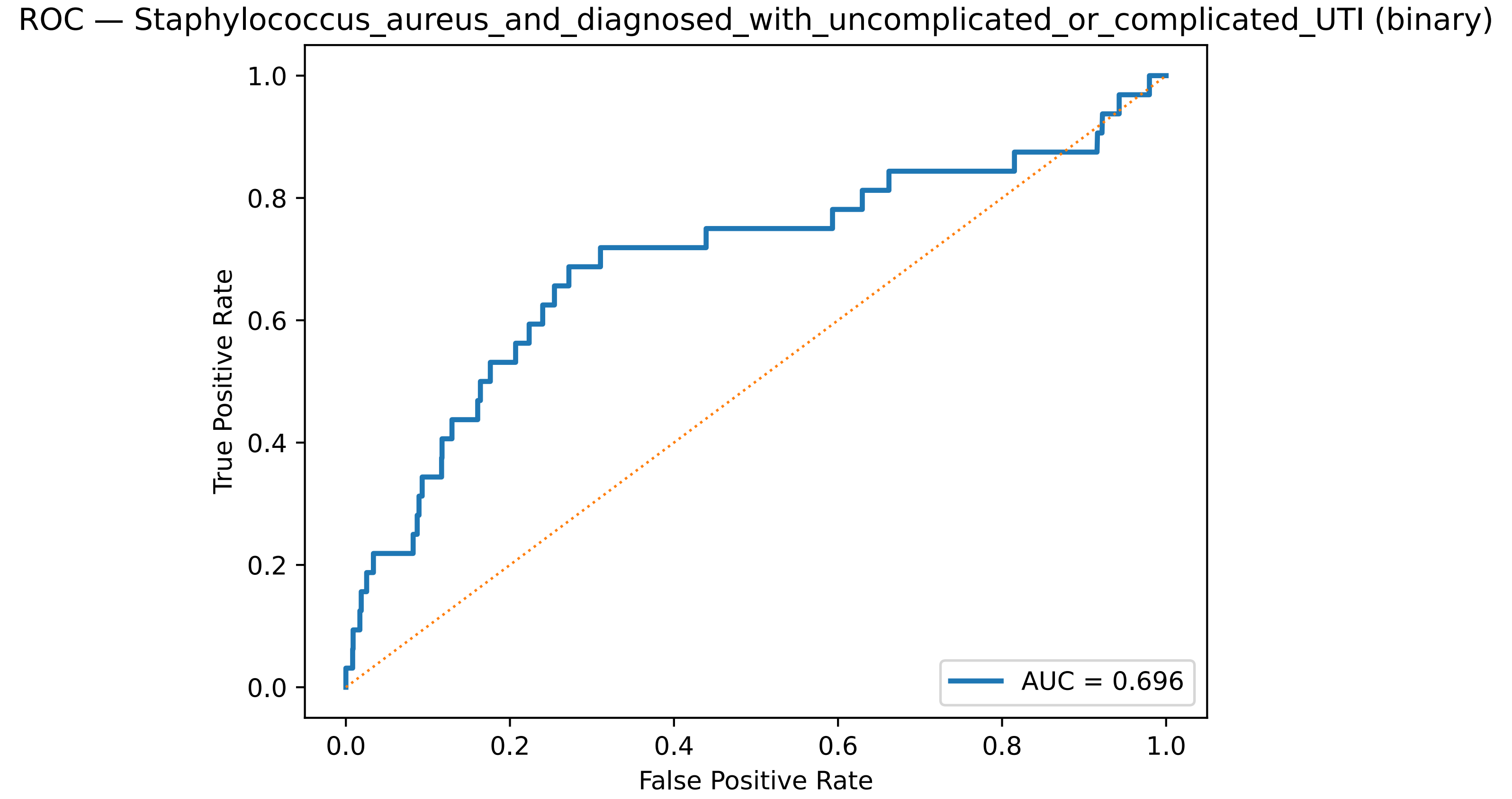
ROC — Staphylococcus_aureus_and_diagnosed_with_uncomplicated_or_complicated_UTI (binary)
True Positive Rate
False Positive Rate
0.0
0.2
0.4
0.6
0.8
1.0
AUC = 0.696

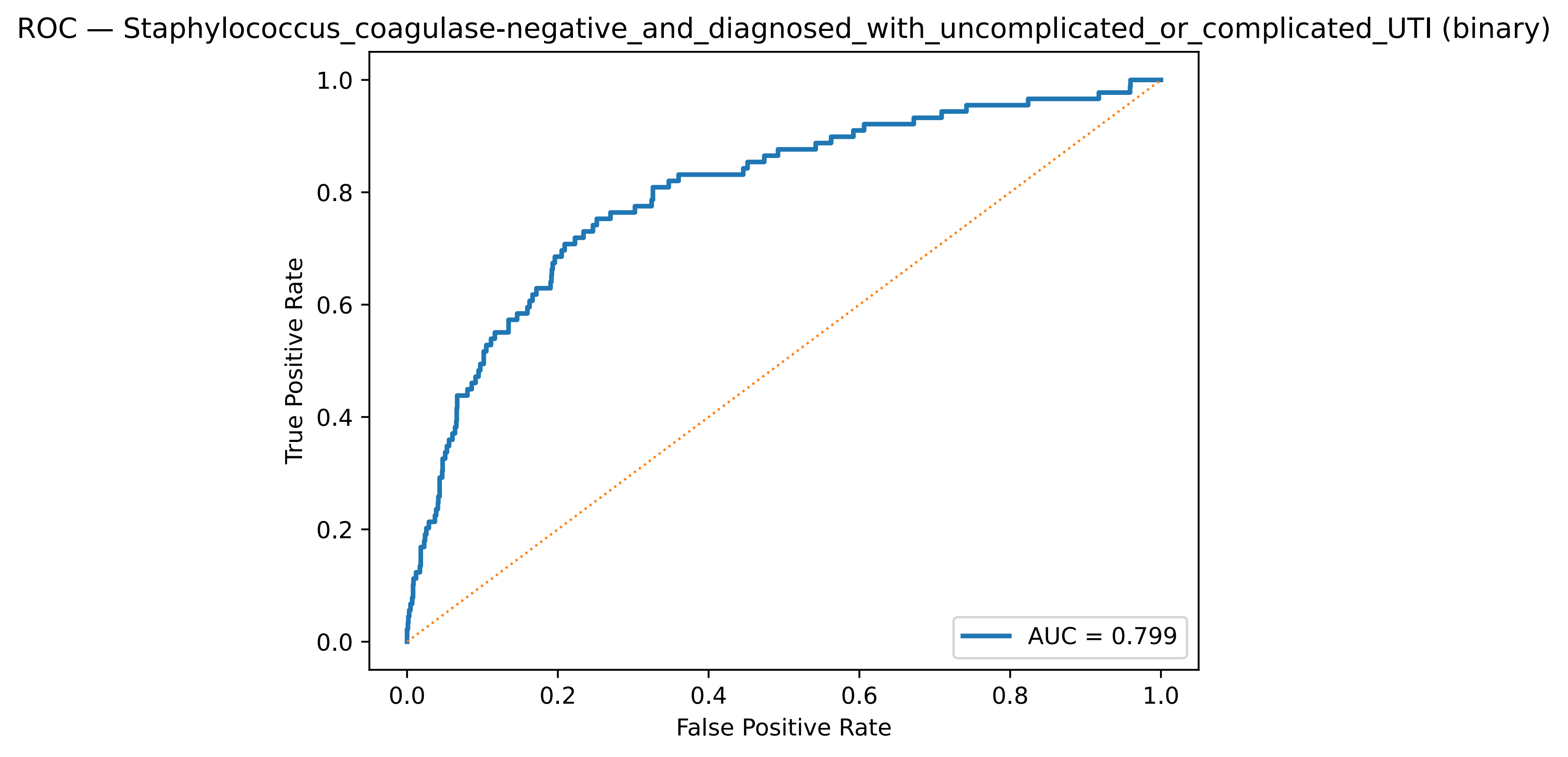

ROC — Staphylococcus_coagulase-negative_and_diagnosed_with_uncomplicated_or_complicated_UTI (binary)
True Positive Rate
False Positive Rate
0.0
0.2
0.4
0.6
0.8
1.0
AUC = 0.799

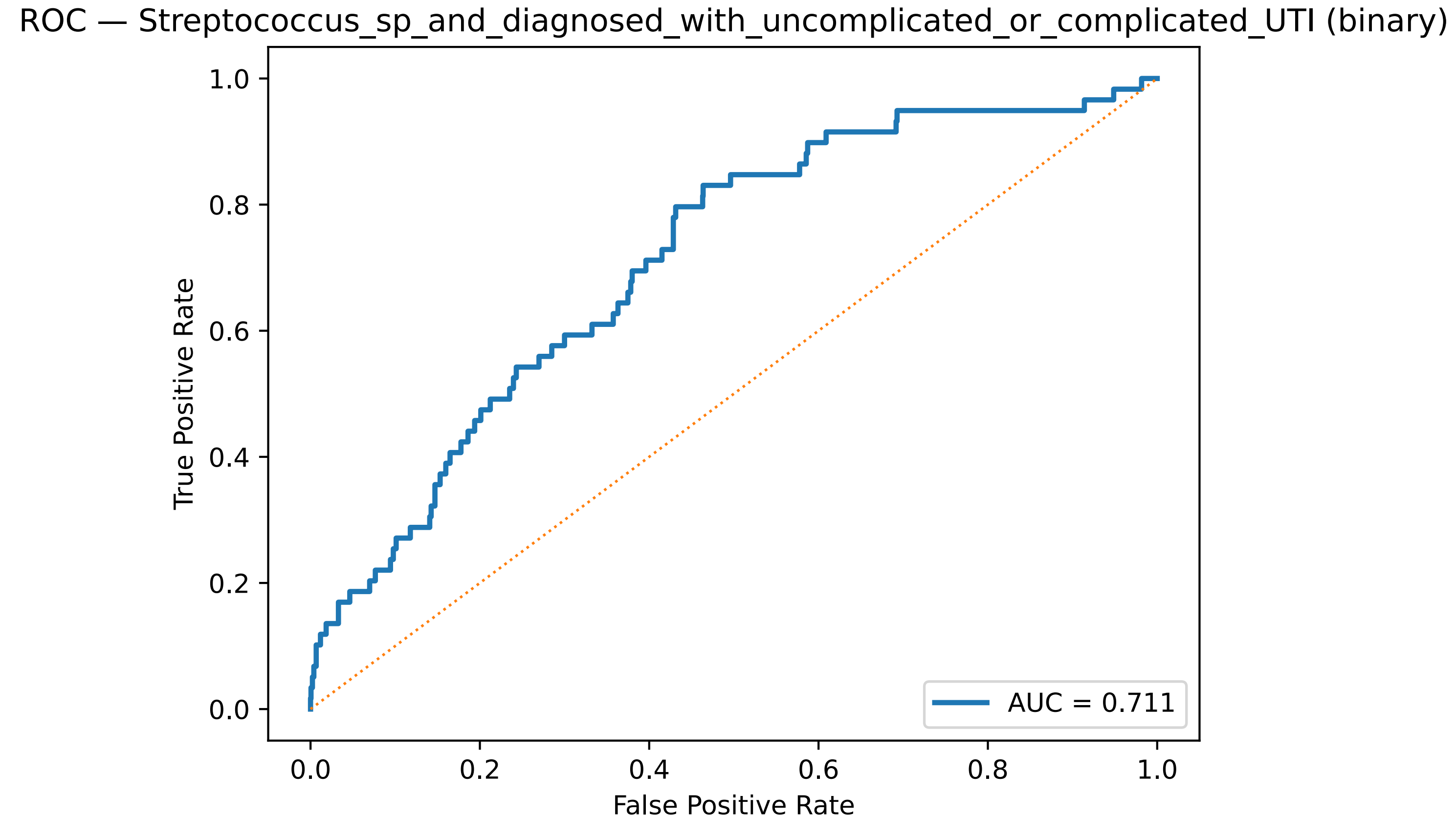
ROC — Streptococcus_sp_and_diagnosed_with_uncomplicated_or_complicated_UTI (binary)
True Positive Rate
False Positive Rate
0.0
0.2
0.4
0.6
0.8
1.0
AUC = 0.711

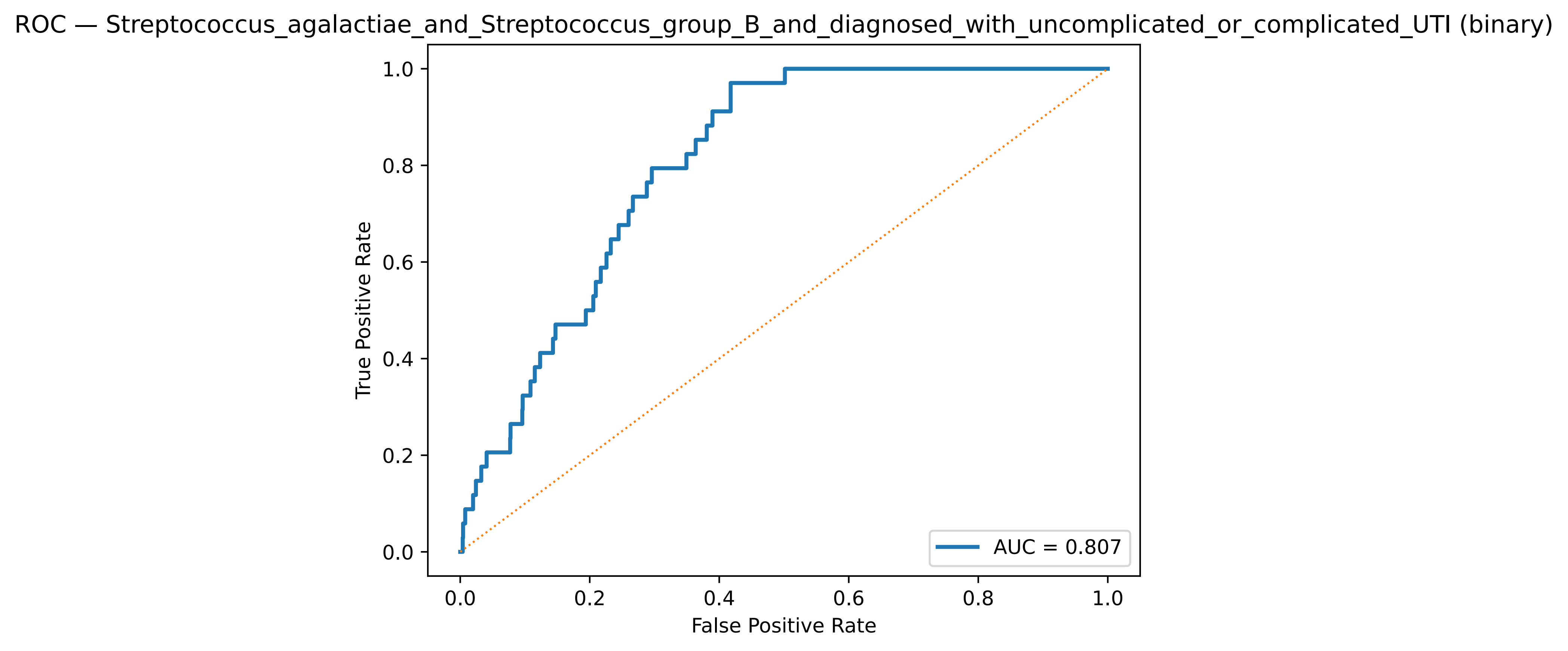

ROC — Streptococcus_agalactiae_and_Streptococcus_group_B_and_diagnosed_with_uncomplicated_or_complicated_UTI (binary)
True Positive Rate
False Positive Rate
0.0
0.2
0.4
0.6
0.8
1.0
AUC = 0.807

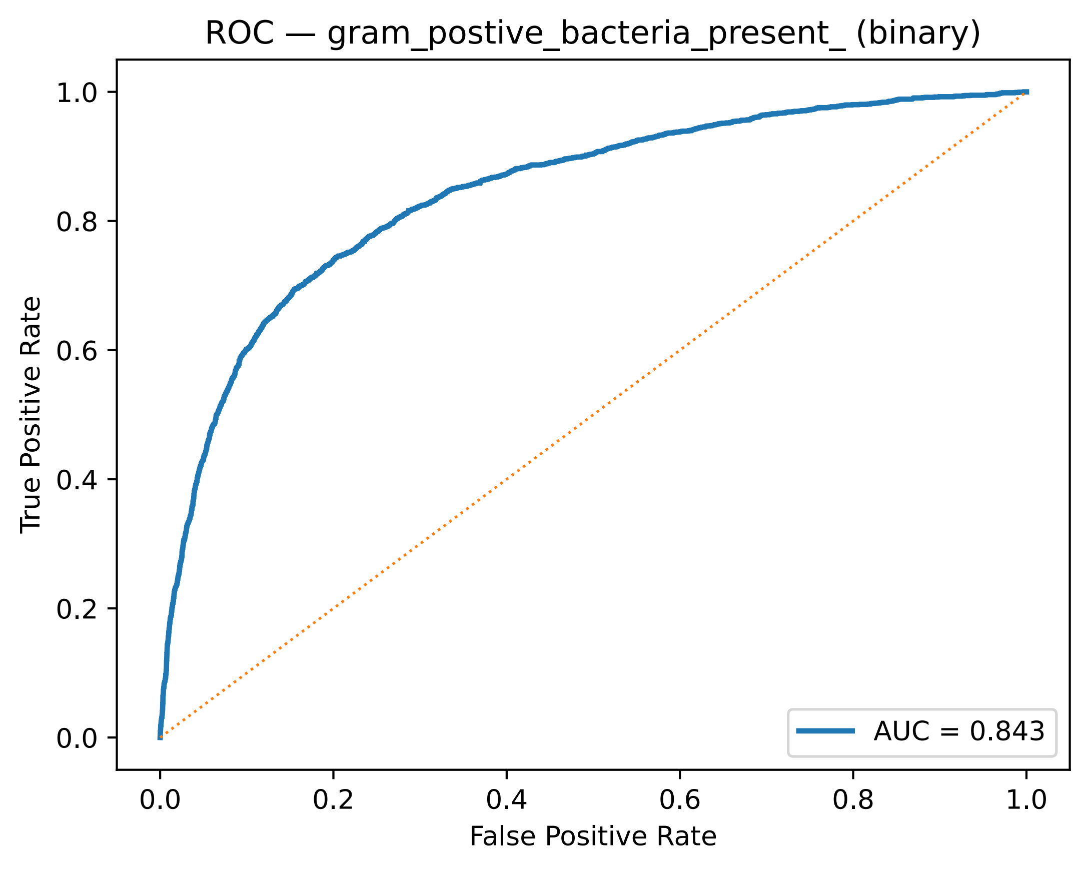
ROC — gram_postive_bacteria_present_ (binary)
True Positive Rate
False Positive Rate
0.0
0.2
0.4
0.6
0.8
1.0
AUC = 0.843

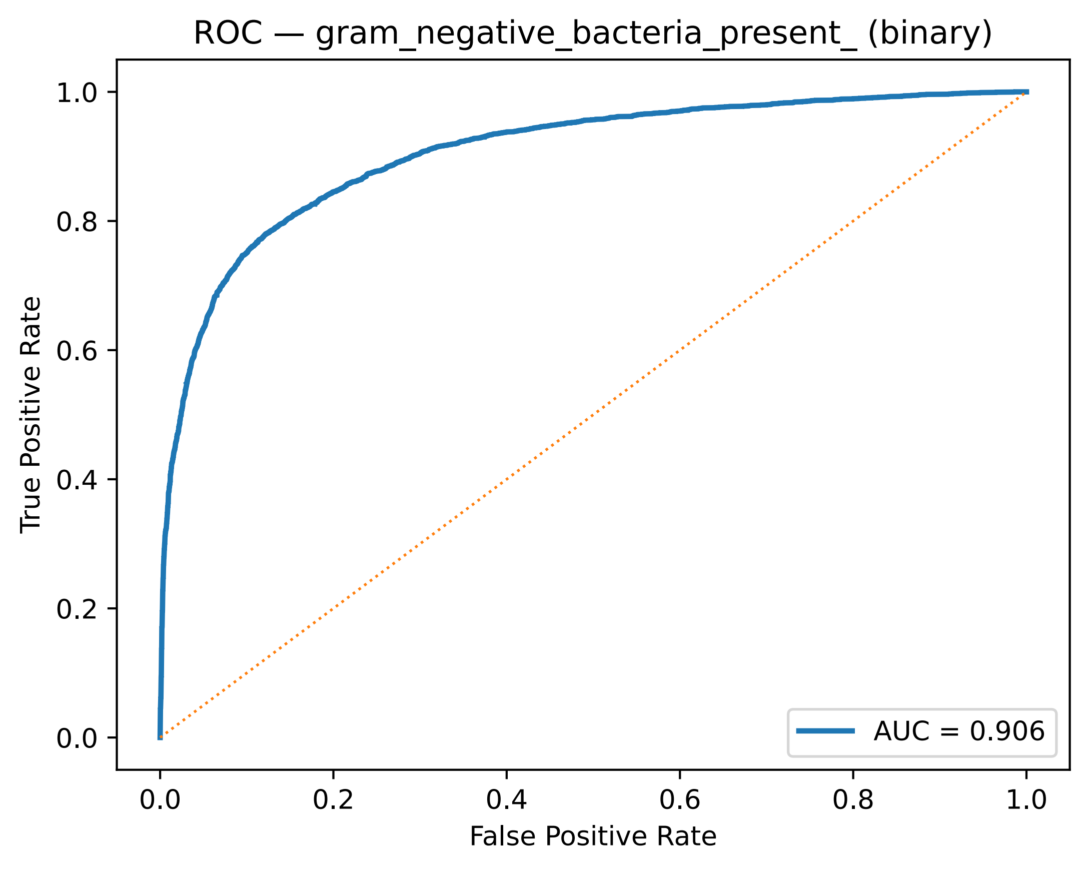
ROC — gram_negative_bacteria_present_ (binary)
True Positive Rate
False Positive Rate
0.0
0.2
0.4
0.6
0.8
1.0
0.0
0.2
0.4
0.6
0.8
1.0
AUC = 0.906

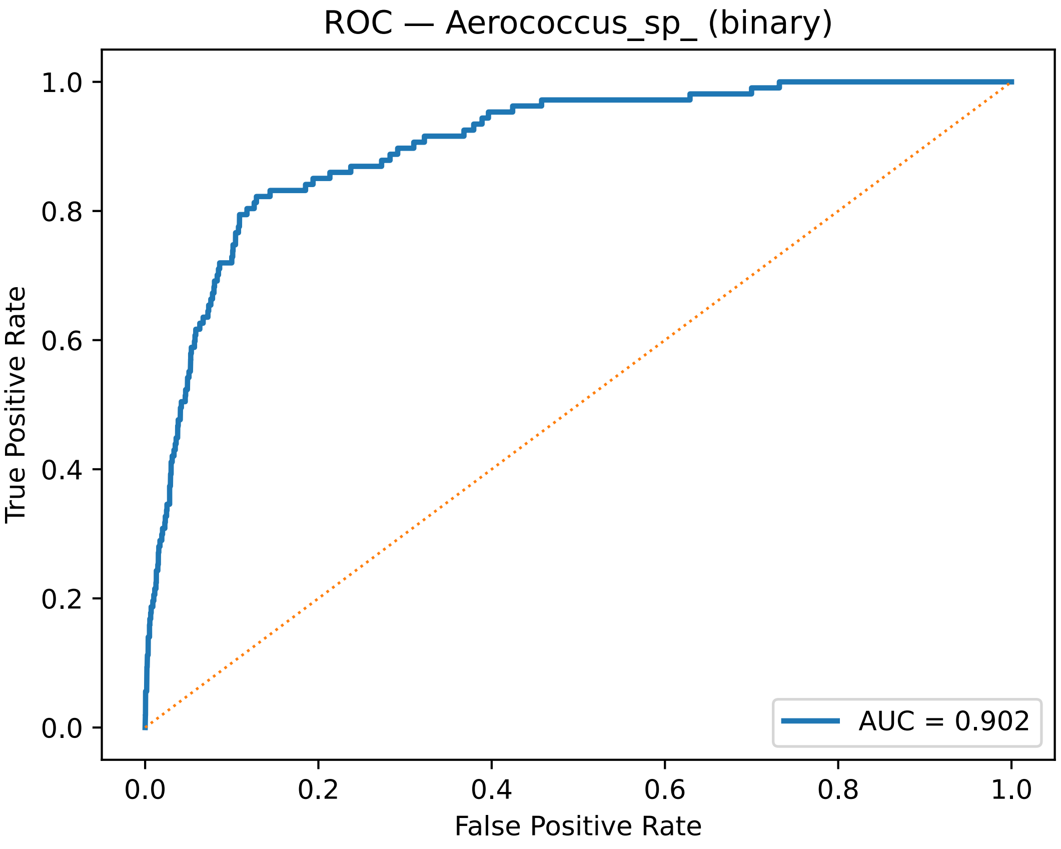
ROC — Aerococcus_sp_ (binary)
True Positive Rate
False Positive Rate
0.0
0.2
0.4
0.6
0.8
1.0
AUC = 0.902

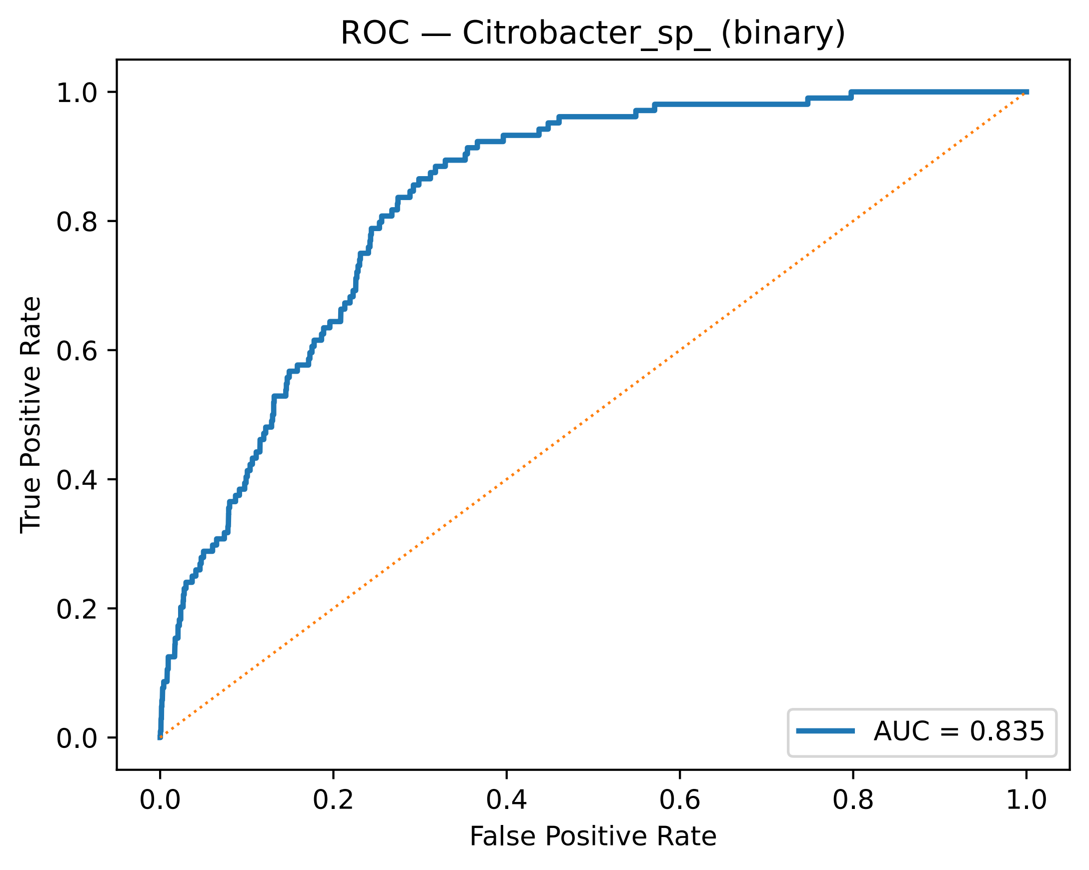
ROC — Citrobacter_sp_ (binary)
True Positive Rate
False Positive Rate
0.0
0.2
0.4
0.6
0.8
1.0
AUC = 0.835

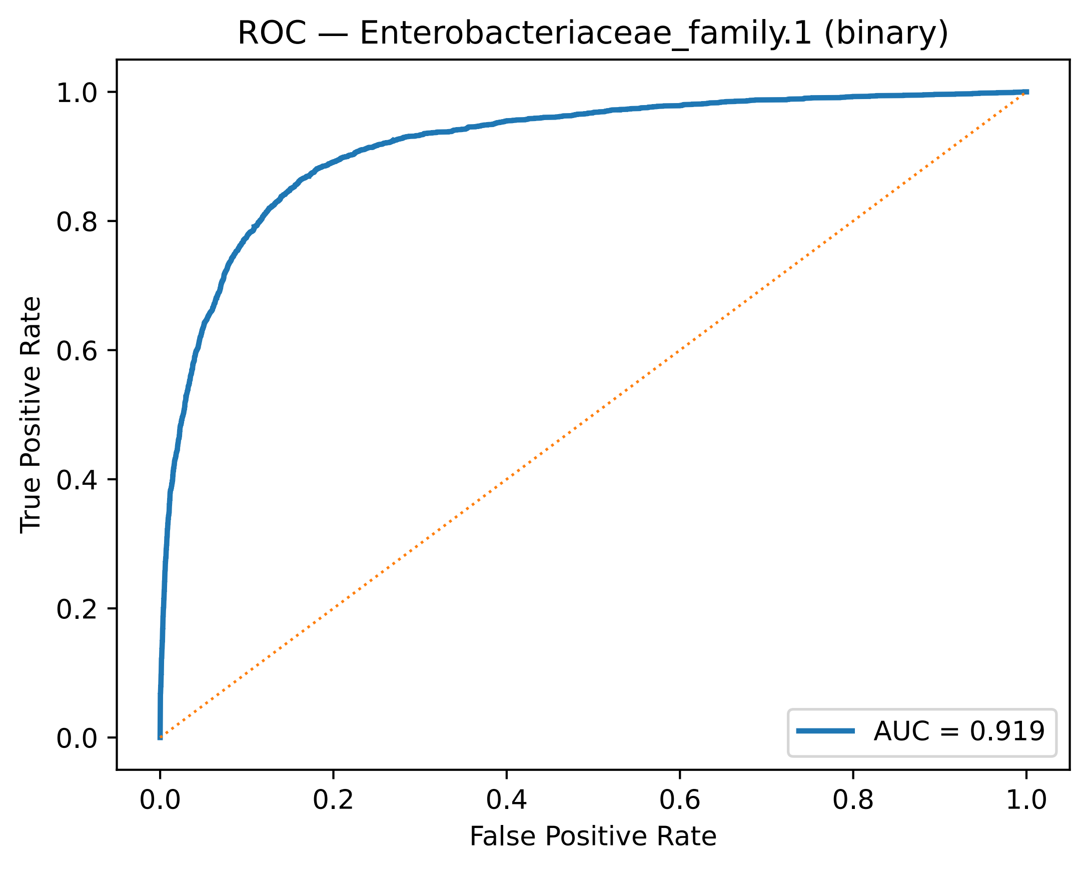
ROC — Enterobacteriaceae_family.1 (binary)
True Positive Rate
False Positive Rate
0.0
0.2
0.4
0.6
0.8
1.0
AUC = 0.919

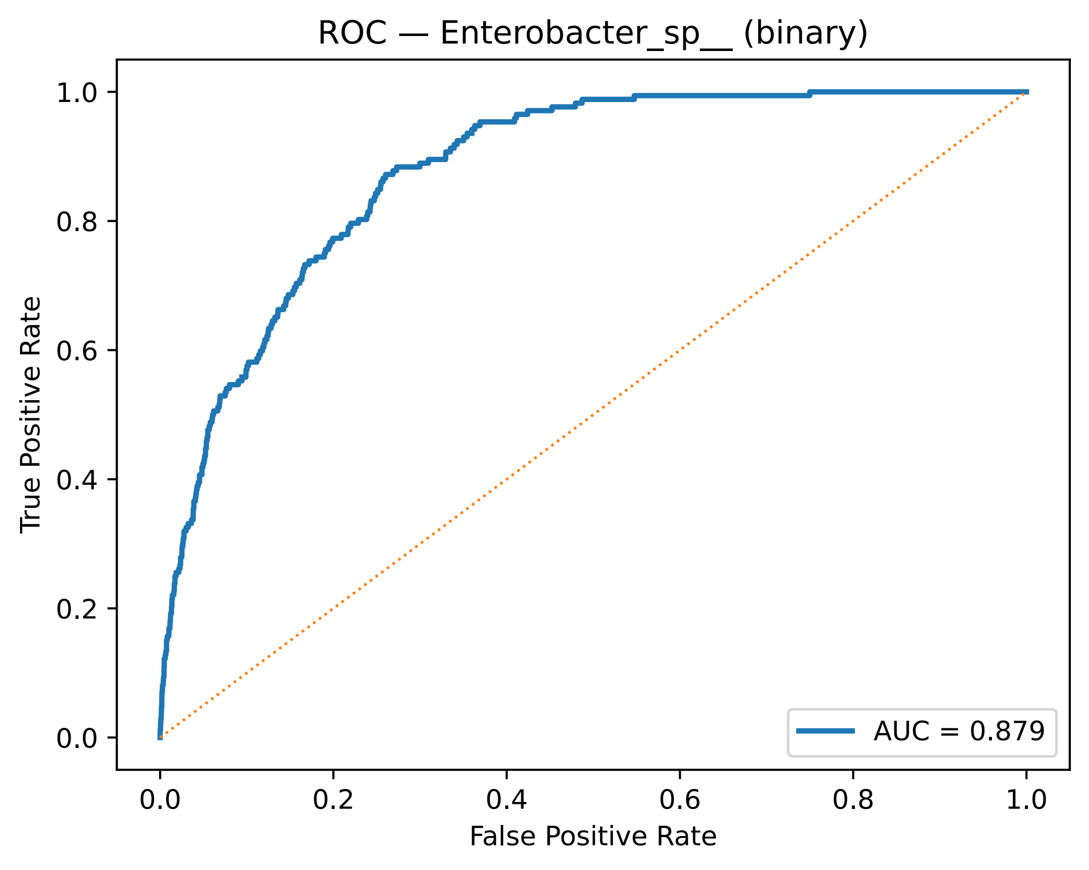
ROC — Enterobacter_sp__ (binary)
True Positive Rate
False Positive Rate
0.0
0.2
0.4
0.6
0.8
1.0
AUC = 0.879

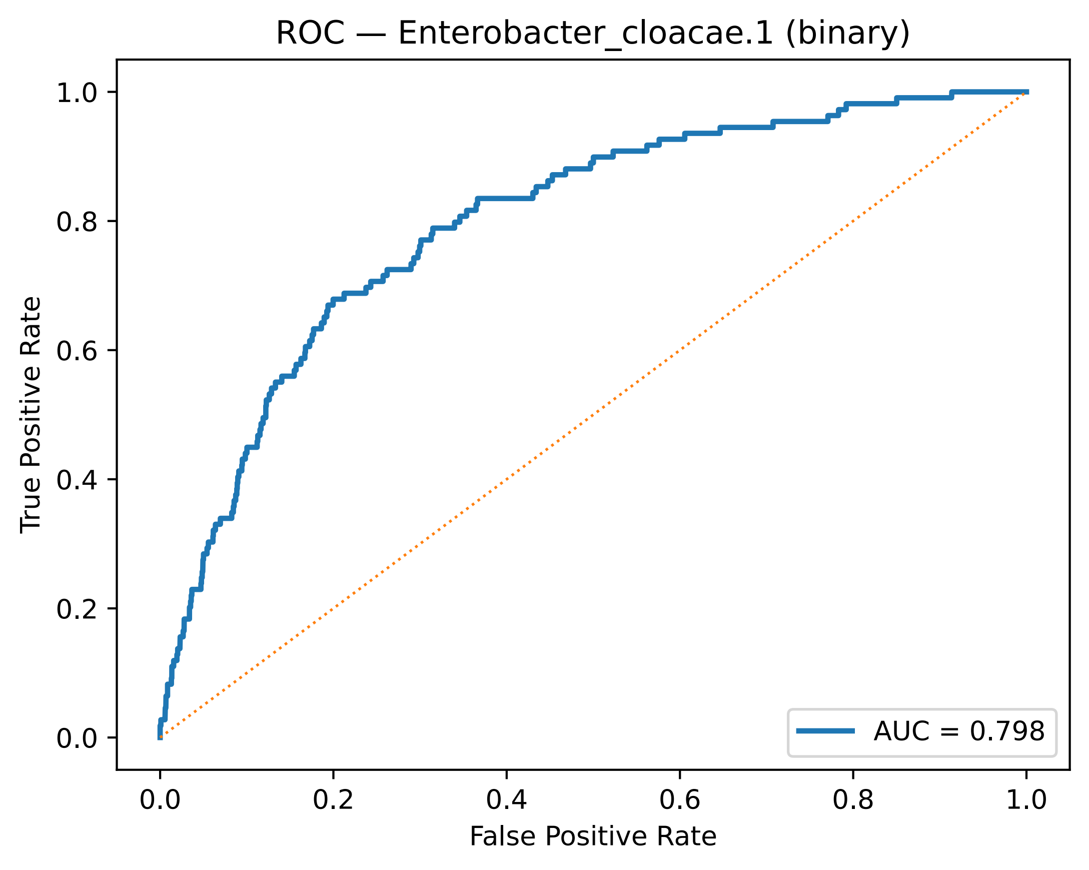
ROC — Enterobacter_cloacae.1 (binary)
True Positive Rate
False Positive Rate
0.0
0.2
0.4
0.6
0.8
1.0
AUC = 0.798

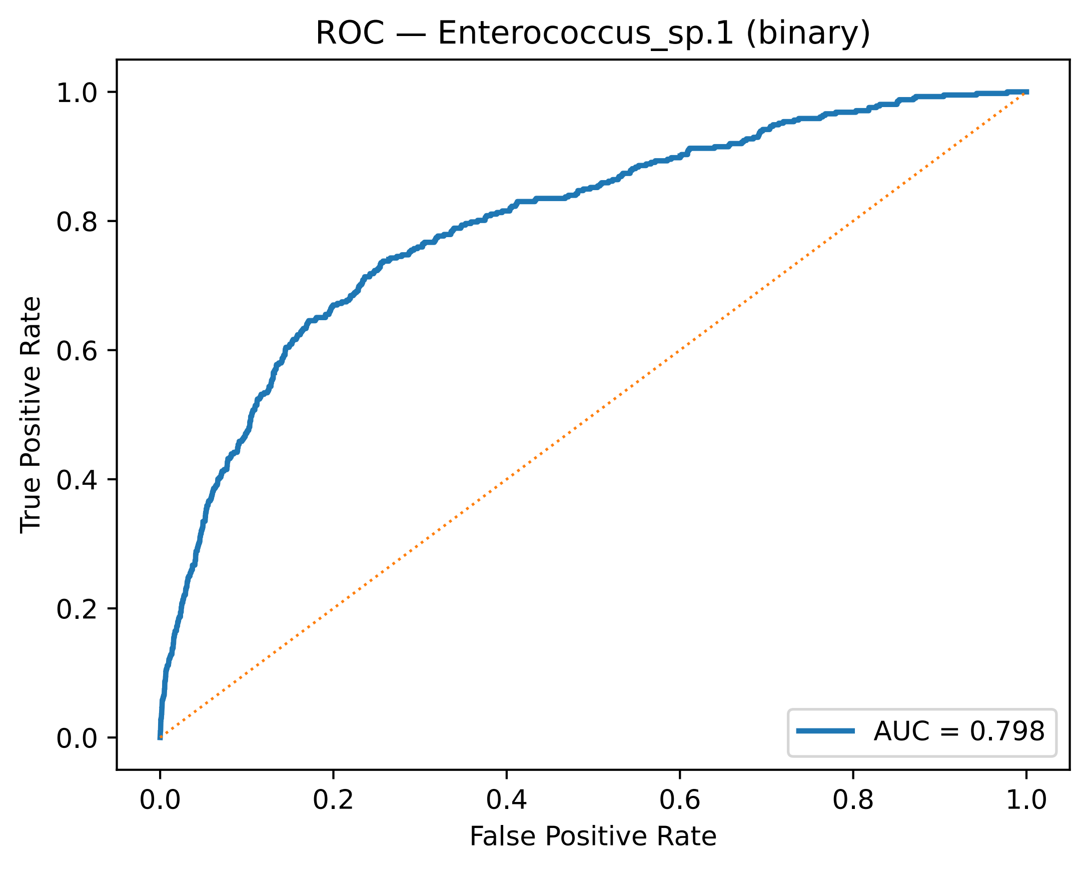

ROC — Enterococcus_sp.1 (binary)
True Positive Rate
False Positive Rate
0.0
0.2
0.4
0.6
0.8
1.0
AUC = 0.798

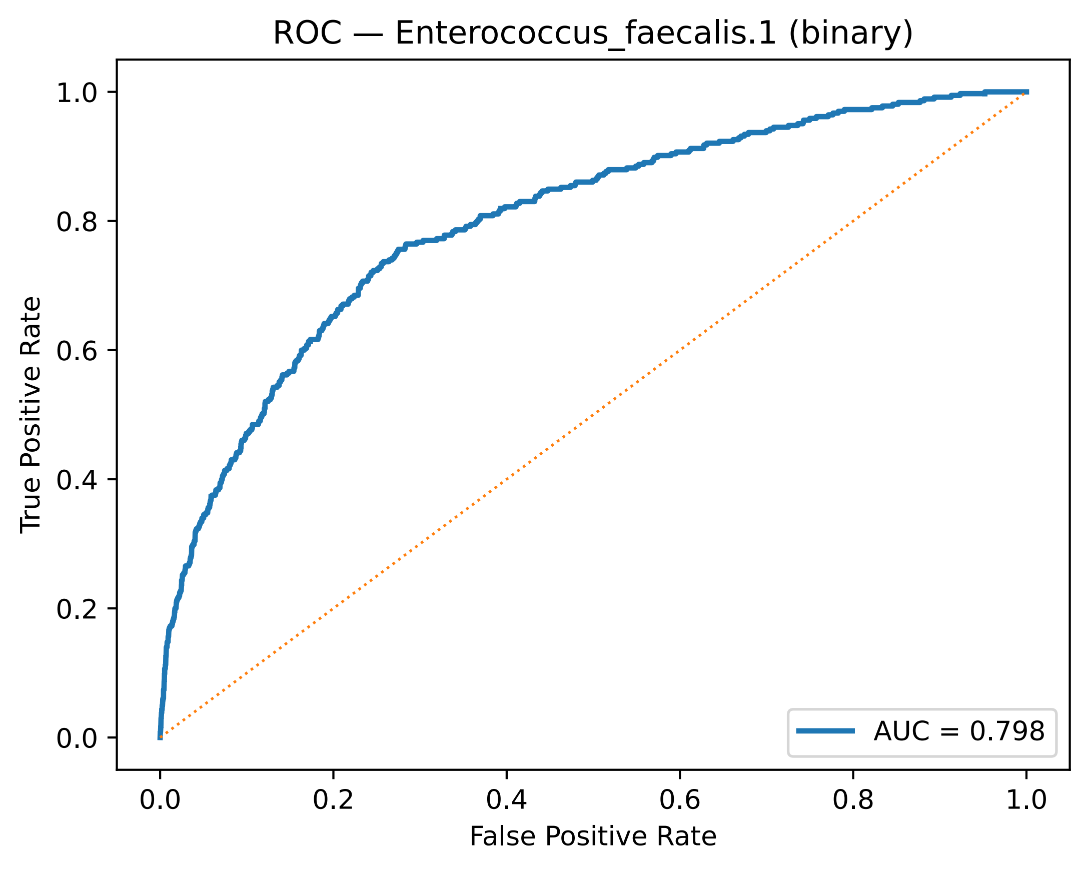
ROC — Enterococcus_faecalis.1 (binary)
True Positive Rate
False Positive Rate
0.0
0.2
0.4
0.6
0.8
1.0
AUC = 0.798

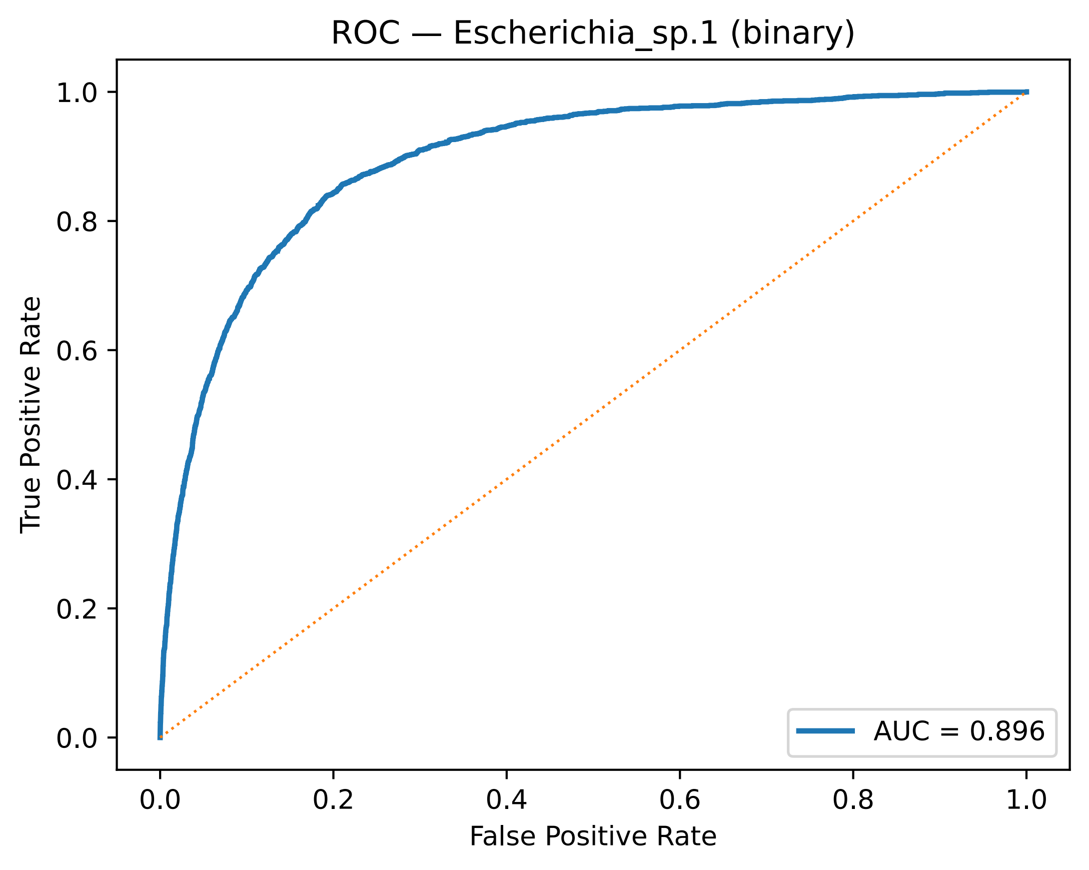
ROC — Escherichia_sp.1 (binary)
True Positive Rate
False Positive Rate
0.0
0.2
0.4
0.6
0.8
1.0
AUC = 0.896

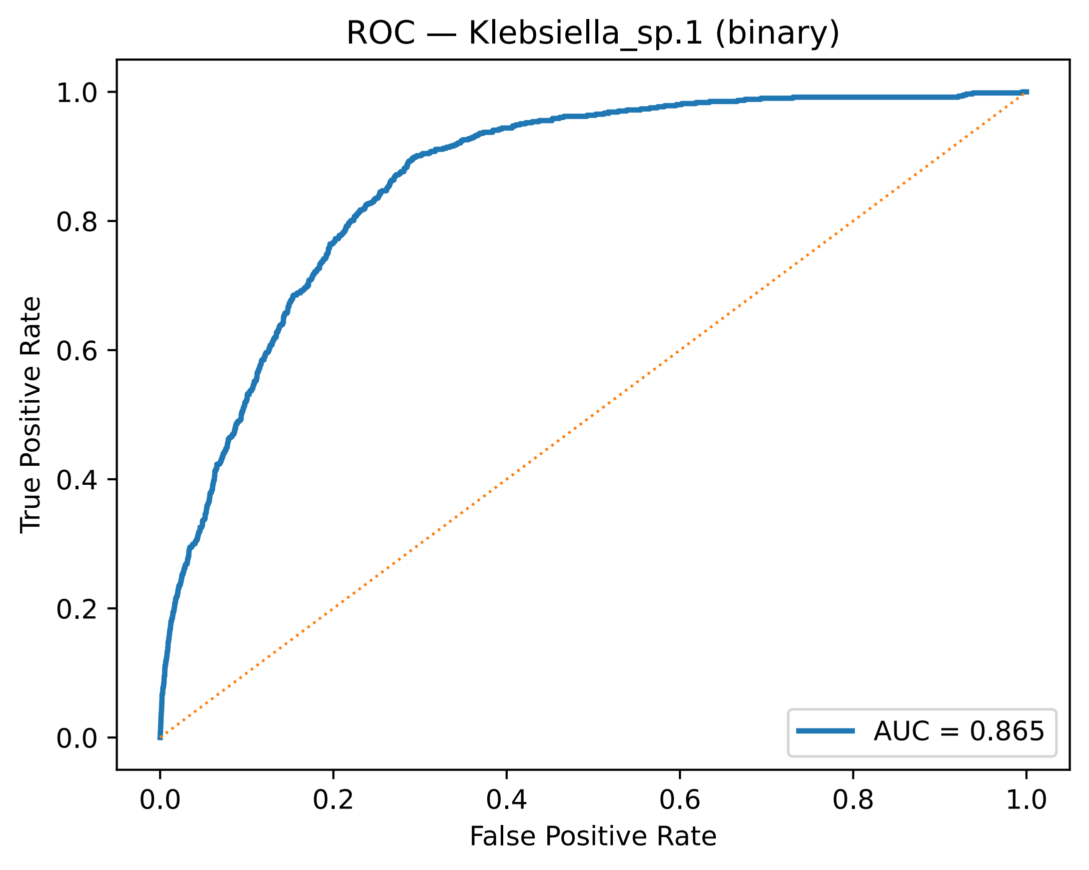
ROC — Klebsiella_sp.1 (binary)
True Positive Rate
False Positive Rate
0.0
0.2
0.4
0.6
0.8
1.0
AUC = 0.865

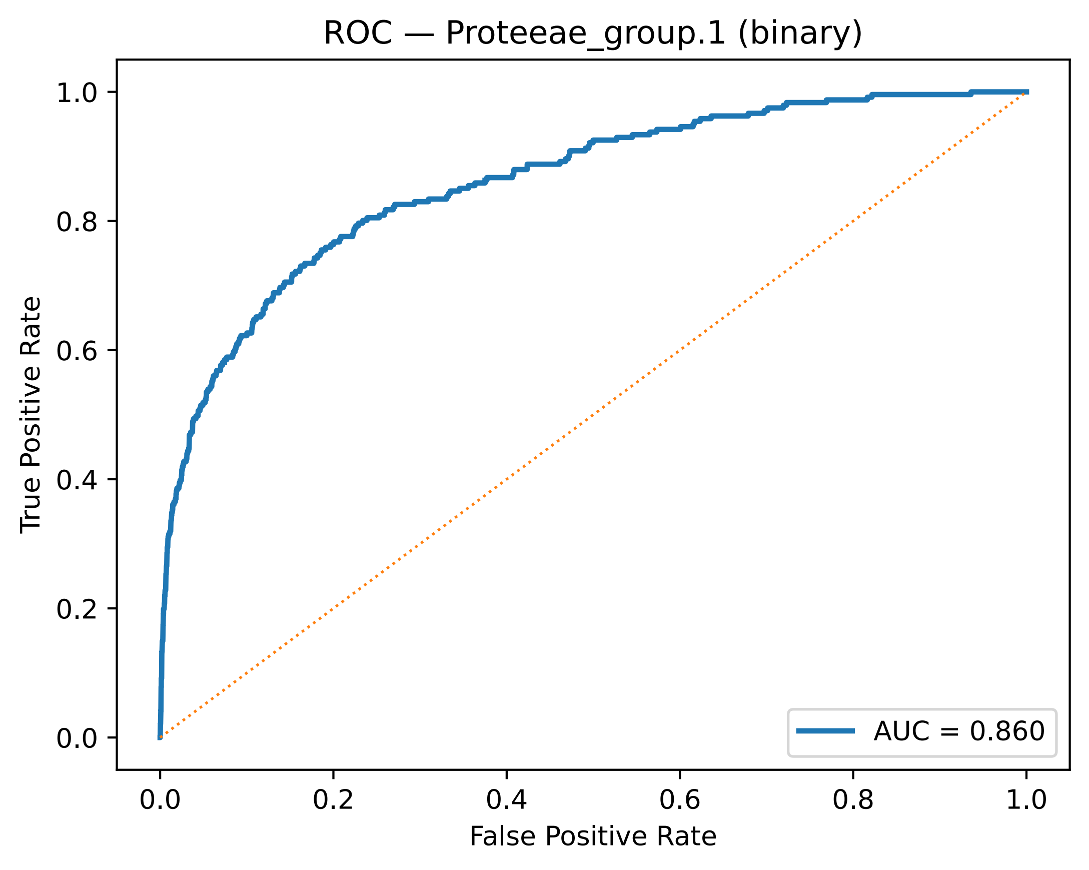
ROC — Proteeae_group.1 (binary)
1.0
0.8
0.6
0.4
0.2
0.0
True Positive Rate
0.0
0.2
0.4
0.6
0.8
1.0
False Positive Rate
AUC = 0.860

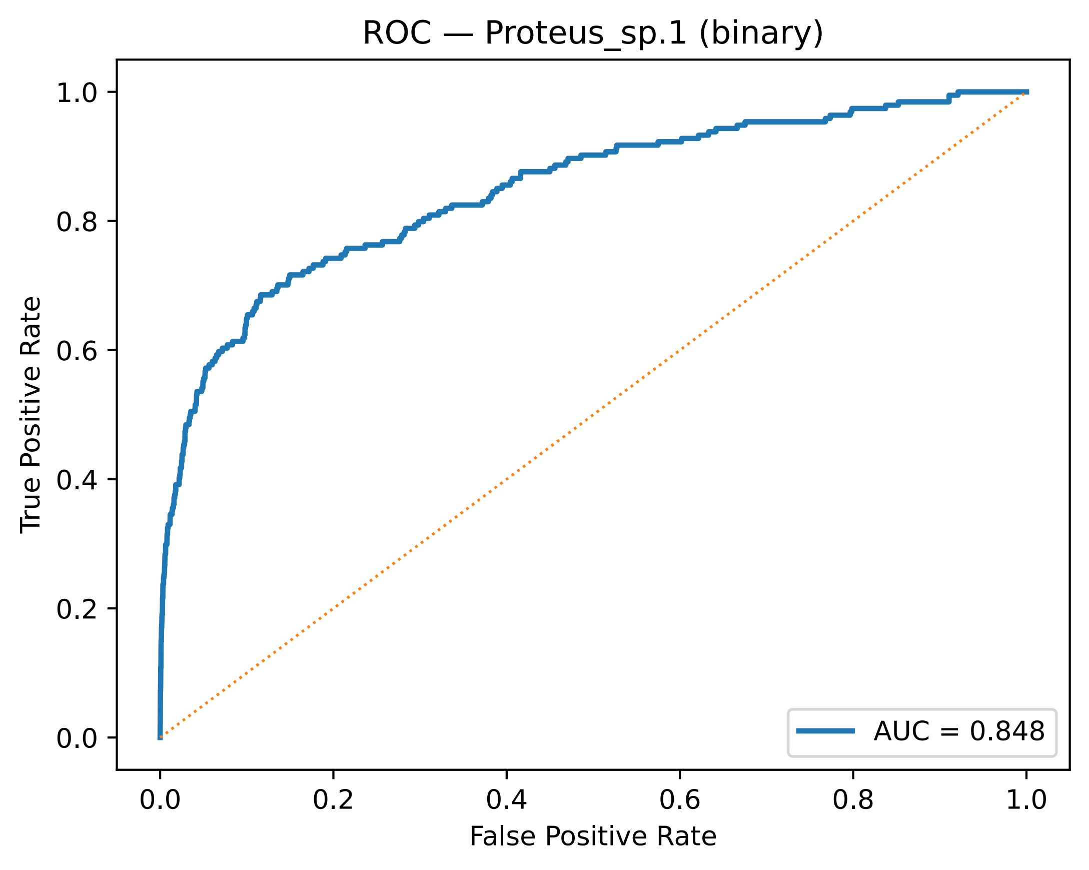
ROC — Proteus_sp.1 (binary)
True Positive Rate
False Positive Rate
0.0
0.2
0.4
0.6
0.8
1.0
AUC = 0.848

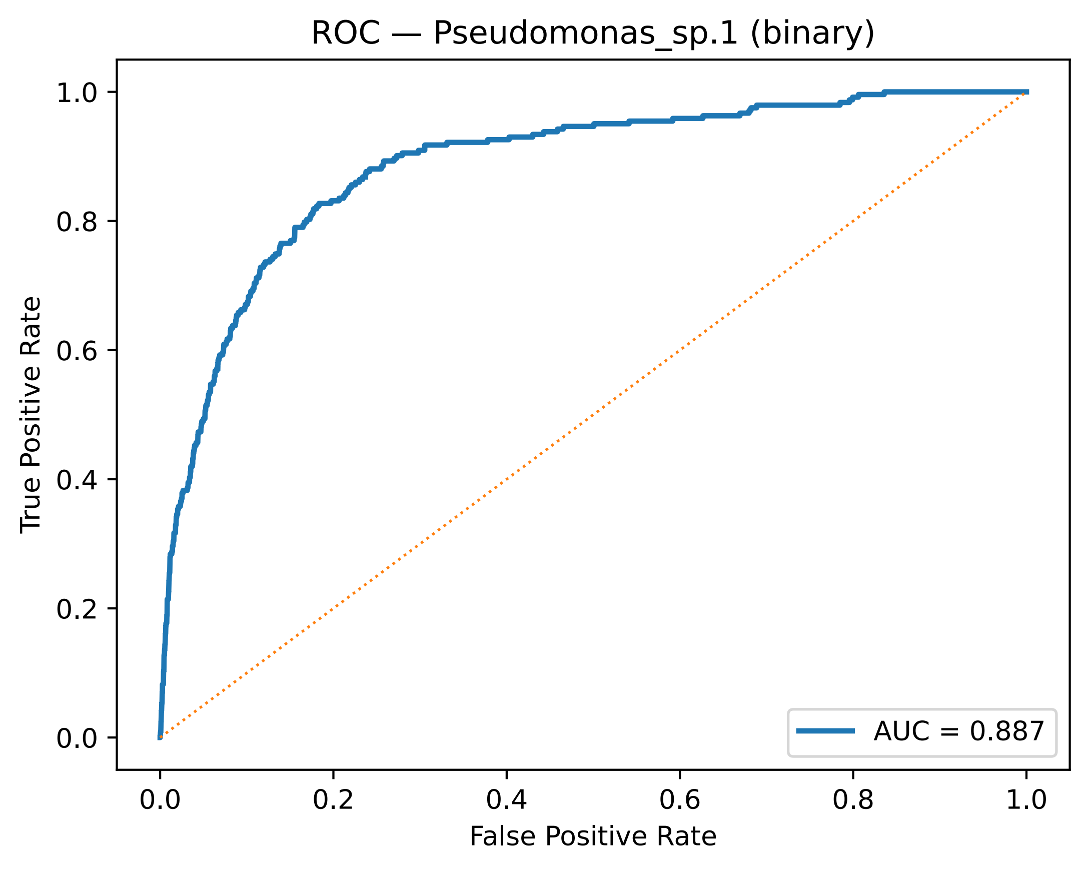

ROC — Pseudomonas_sp.1 (binary)
1.0
0.8
0.6
0.4
0.2
0.0
True Positive Rate
0.0
0.2
0.4
0.6
0.8
1.0
False Positive Rate
AUC = 0.887

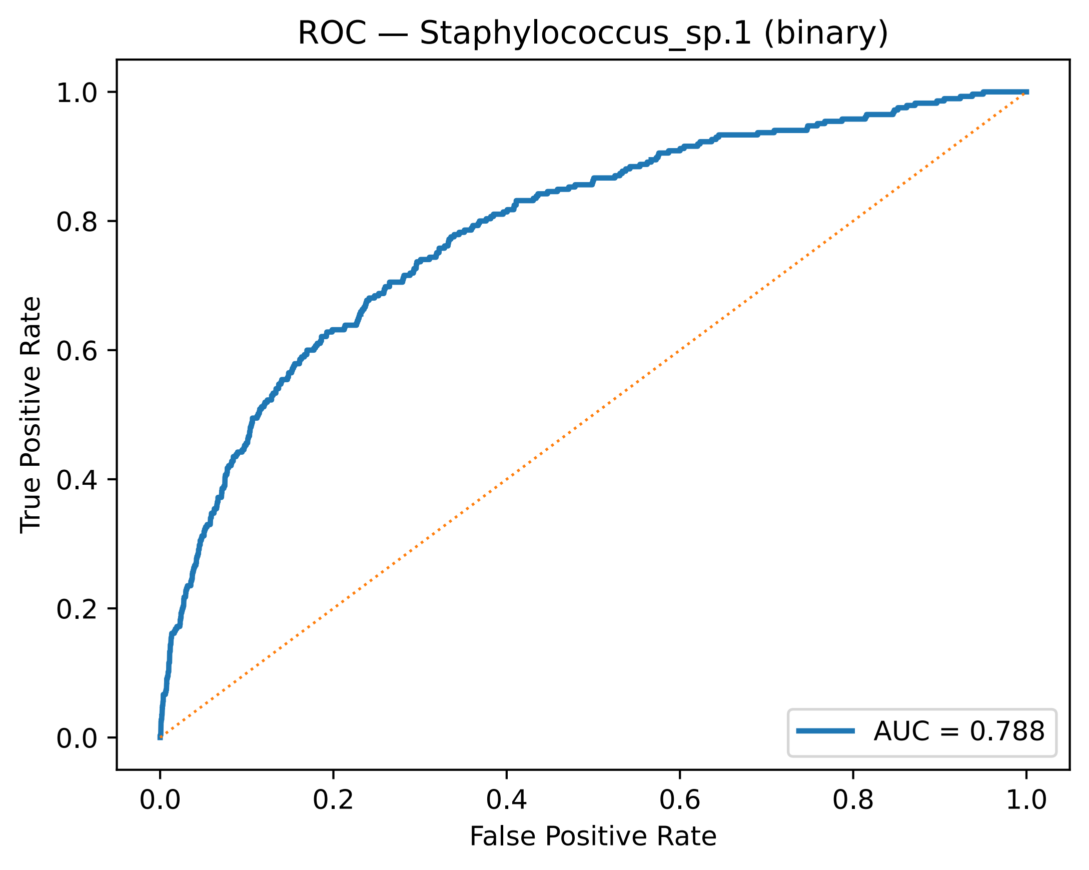
ROC — Staphylococcus_sp.1 (binary)
True Positive Rate
False Positive Rate
0.0
0.2
0.4
0.6
0.8
1.0
AUC = 0.788

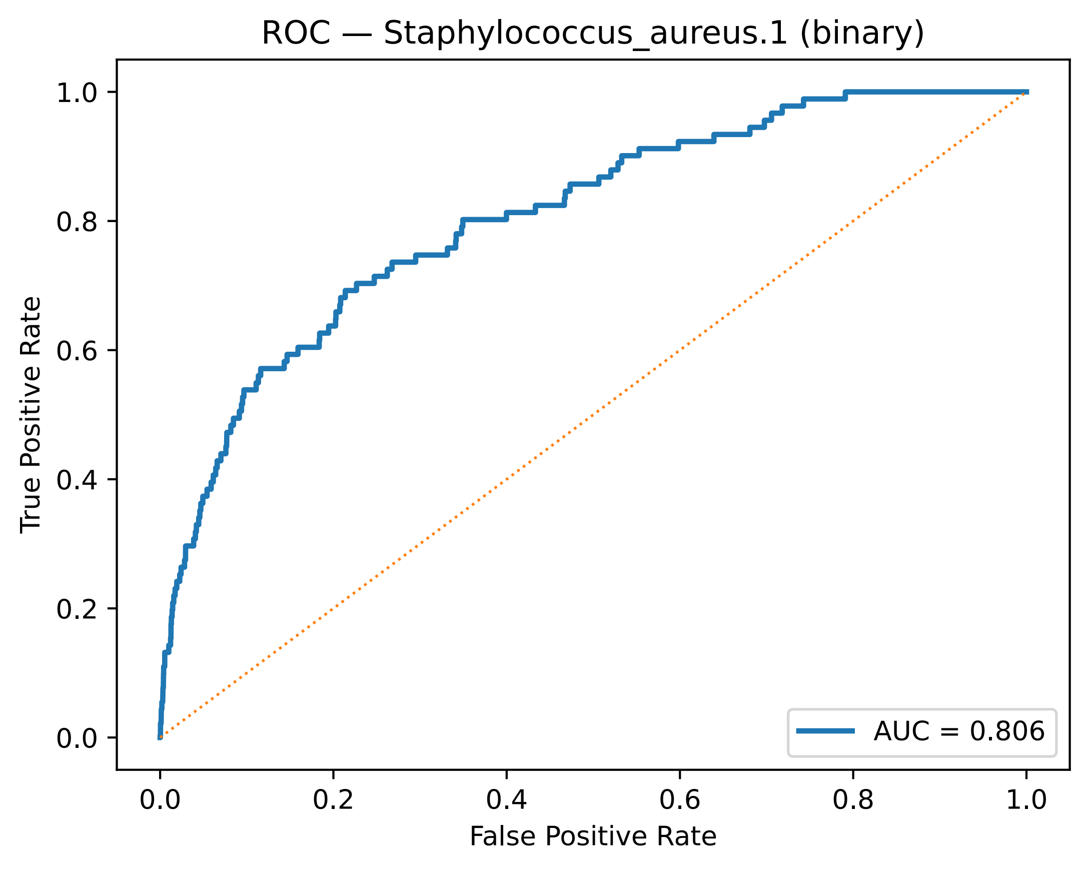
ROC — Staphylococcus_aureus.1 (binary)
True Positive Rate
False Positive Rate
0.0
0.2
0.4
0.6
0.8
1.0
AUC = 0.806

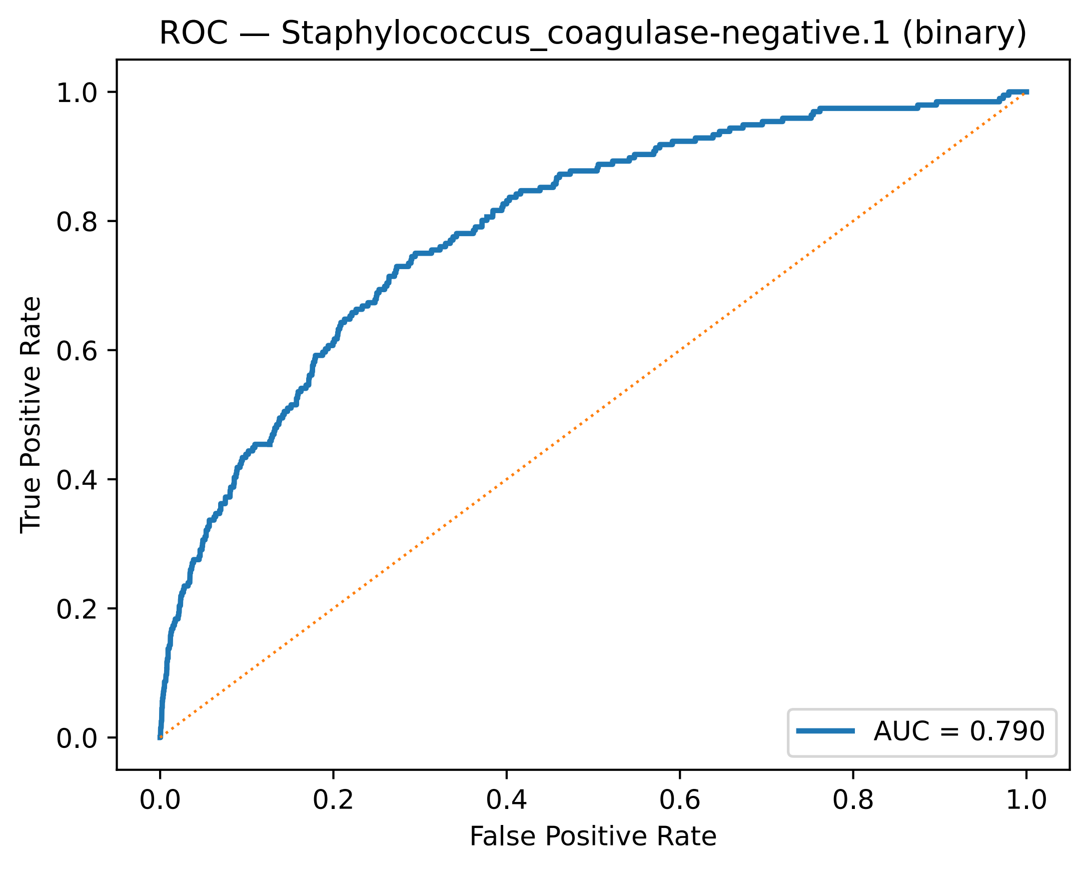

ROC — Staphylococcus_coagulase-negative.1 (binary)
True Positive Rate
False Positive Rate
0.0
0.2
0.4
0.6
0.8
1.0
AUC = 0.790

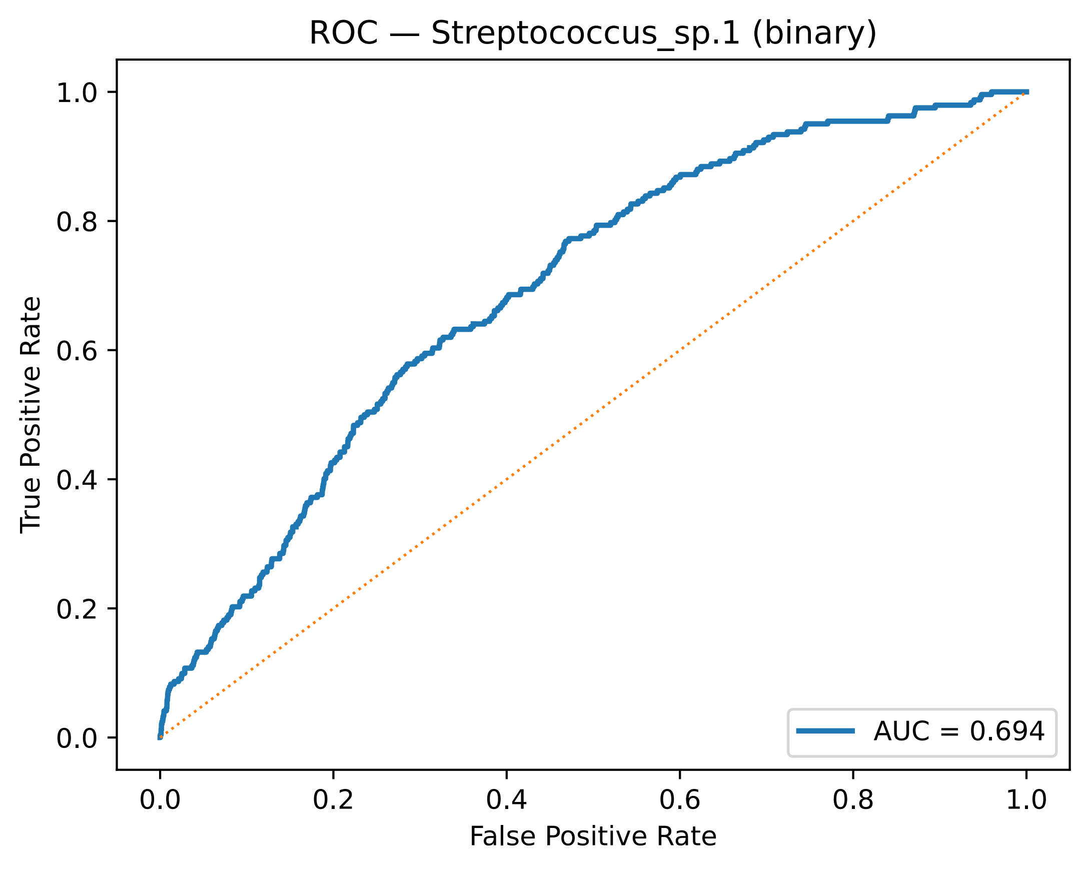
ROC — Streptococcus_sp.1 (binary)
True Positive Rate
False Positive Rate
0.0
0.2
0.4
0.6
0.8
1.0
AUC = 0.694

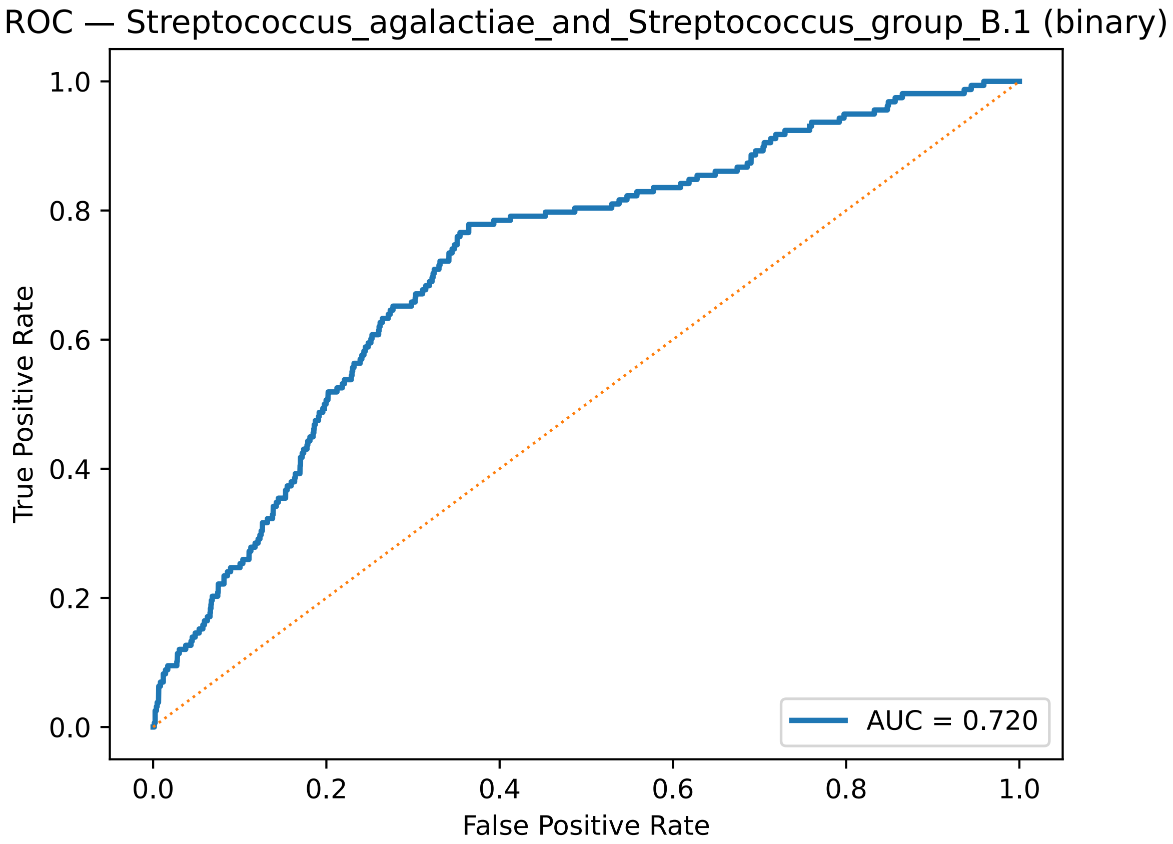
ROC — Streptococcus_agalactiae_and_Streptococcus_group_B.1 (binary)
True Positive Rate
False Positive Rate
0.0
0.2
0.4
0.6
0.8
1.0
AUC = 0.720

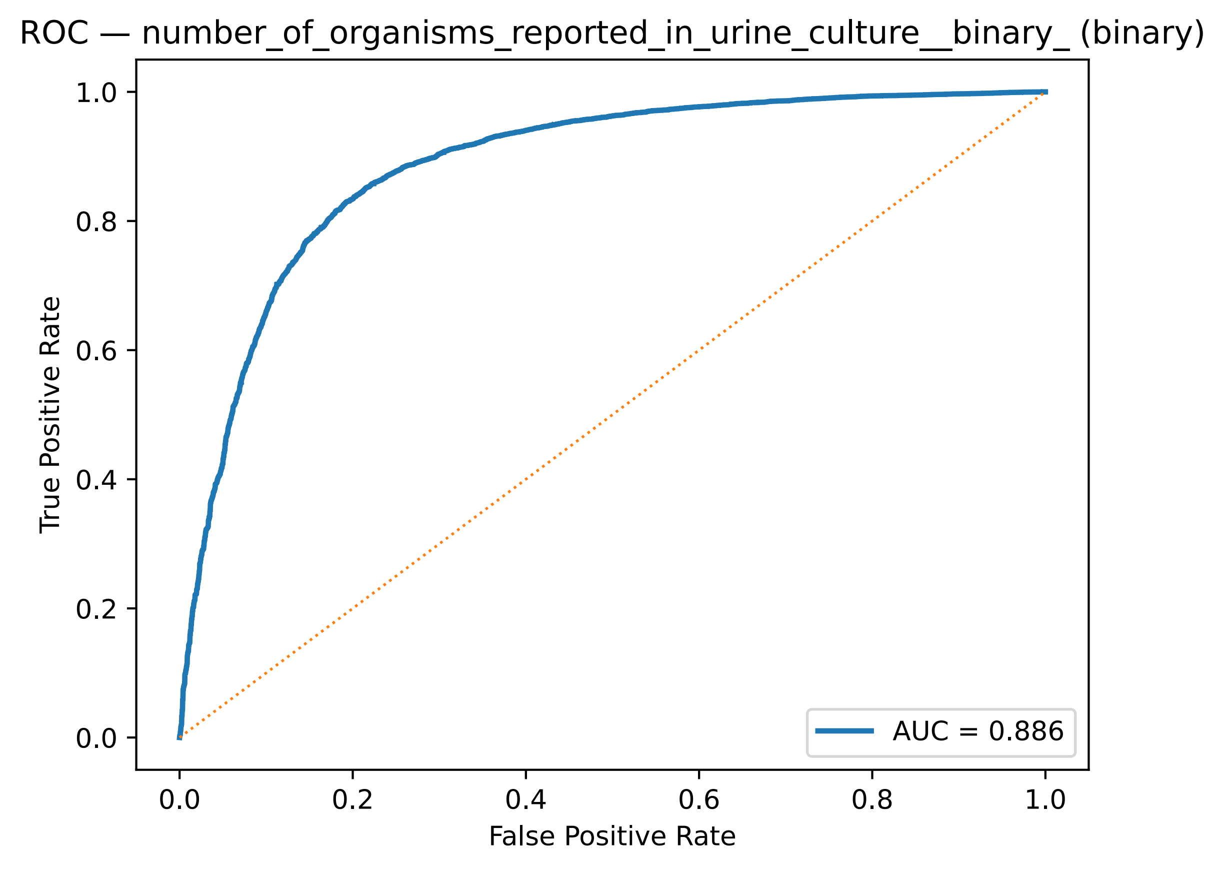
ROC — number_of_organisms_reported_in_urine_culture__binary_ (binary)
True Positive Rate
False Positive Rate
0.0
0.2
0.4
0.6
0.8
1.0
AUC = 0.886
